# Astrocytic ankyrin-2 enables memory persistence in the mouse hippocampus

**DOI:** 10.1038/s41467-026-75009-5

**Published:** 2026-07-07

**Authors:** Hayoung Kim, Jiwoon Lim, Jooyoung Kim, Erva Ozkan, Gyu Hyun Kim, HyoJin Park, Mingu Gordon Park, Bitna Joo, Sangkyu Lee, Kea Joo Lee, Bong-Kiun Kaang, C. Justin Lee, Wuhyun Koh

**Affiliations:** 1 Center for Memory and Glioscience, Institute for Basic Science (IBS), Daejeon, Republic of Korea; 2 IBS School, Korea University of Science and Technology (UST), Daejeon, Republic of Korea; 3 Department of Bioscience and Biotechnology, Chungnam National University, Daejeon, Republic of Korea; 4 Neural Circuits Research Group, Korea Brain Research Institute, Daegu, Republic of Korea; 5 School of Biological Sciences, Seoul National University, Seoul, Republic of Korea

**Keywords:** Cellular neuroscience, Astrocyte, Long-term memory

## Abstract

Memory persistence, the ability to retain information over time, is a fundamental feature of long-term memory. Although astrocytes contribute to synaptic plasticity, the molecular mechanisms by which they support memory persistence remain unclear. Here we show that astrocytic ankyrin-2 (Ank2) is required for memory persistence in adult mice. Astrocyte-specific deletion of Ank2 impaired remote memory without affecting recent memory and disrupted the maintenance of long-term potentiation. Loss of Ank2 reduced astrocyte contacts with engram neurons and impaired astrocyte morphogenesis driven by brain-derived neurotrophic factor (BDNF) signaling through the truncated tropomyosin receptor kinase B receptor (TrkB.T1) and inositol 1,4,5-trisphosphate receptor type 2 (IP3R2). Consistent with this mechanism, astrocytic Ank2 was required for the enhancement of memory persistence by hippocampal BDNF infusion. Furthermore, selective optogenetic activation of astrocytic TrkB.T1 signaling enhanced remote memory, demonstrating that astrocytic BDNF signaling is sufficient to promote memory persistence. These findings identify astrocytic Ank2 as a key regulator of long-term memory persistence.

## Abstract (web summary)

This study shows that astrocytic Ankyrin-2 is required for astrocyte morphogenesis and BDNF signaling that support remote memory in the hippocampus. Activating this pathway is sufficient to enhance memory persistence in mice.

## Introduction

Memory persistence, the ability to retain learned information over extended periods, is a defining feature of long-term memory and is essential for adaptive behavior. Astrocytes, among the most abundant cells in the brain, actively participate in synaptic transmission, plasticity, and memory^1–3^. Astrocytic leaflets (fine perisynaptic processes) form part of the tripartite synapse^4^ and undergo dynamic remodeling during and after learning^5–8^. Such structural changes can promote the stability of synaptic plasticity^8^ and support memory processing^9,10^, and may also contribute to memory persistence. However, the molecular mechanisms by which astrocytes regulate memory persistence remain largely unknown.

Ankyrin-2 (Ank2, also known as ankyrin-B or brain ankyrin) is an anchoring protein that organizes ion channels and transporters^11^, and its expression in the brain is comparable or higher in astrocytes than in neurons^12–15^. Ank2 has been implicated in intellectual disability^16–18^, epilepsy^16^, and autism spectrum disorder (ASD)^19^. Although previous work has focused almost exclusively on neuronal Ank2 and found no deficits in learning and memory when it is deleted in neurons^16,20,21^, the role of Ank2 in astrocytes remains unknown, particularly in regulating structures and signaling mechanisms that sustain memory persistence. Notably, astrocytes predominantly express the 220 kDa Ank2 isoform, which lacks the neuron-specific domain present in the 440 kDa isoform^15,22^ and shows a progressive increase throughout adulthood^23^. Given that Ank2 has been detected in astrocytic leaflets^24–27^, astrocytic Ank2 may contribute to astrocytic plasticity as well as synaptic plasticity that underlie long-term memory persistence.

Here, we identify astrocytic Ank2 as a regulator of astrocytic contributions to cognition, focusing on its role in memory persistence. We used astrocyte-specific Ank2 deletion to determine whether Ank2 is necessary for memory persistence. In addition, we developed Opto-T1, an optogenetic tool that selectively activates astrocytic TrkB.T1 signaling, to test whether such signaling alone is sufficient to enhance memory persistence. Using molecular, imaging, electrophysiological, and behavioral approaches, including astrocyte-eGRASP to visualize astrocytic process contacts with engram neurons^10^, we examined how Ank2-dependent astrocytic leaflets support engram neuron contact, maintain long-term potentiation, and enable the persistence of long-term memory.

## Results

### Astrocytic Ank2 deletion leads to reduced memory persistence

**Fig. 1:**
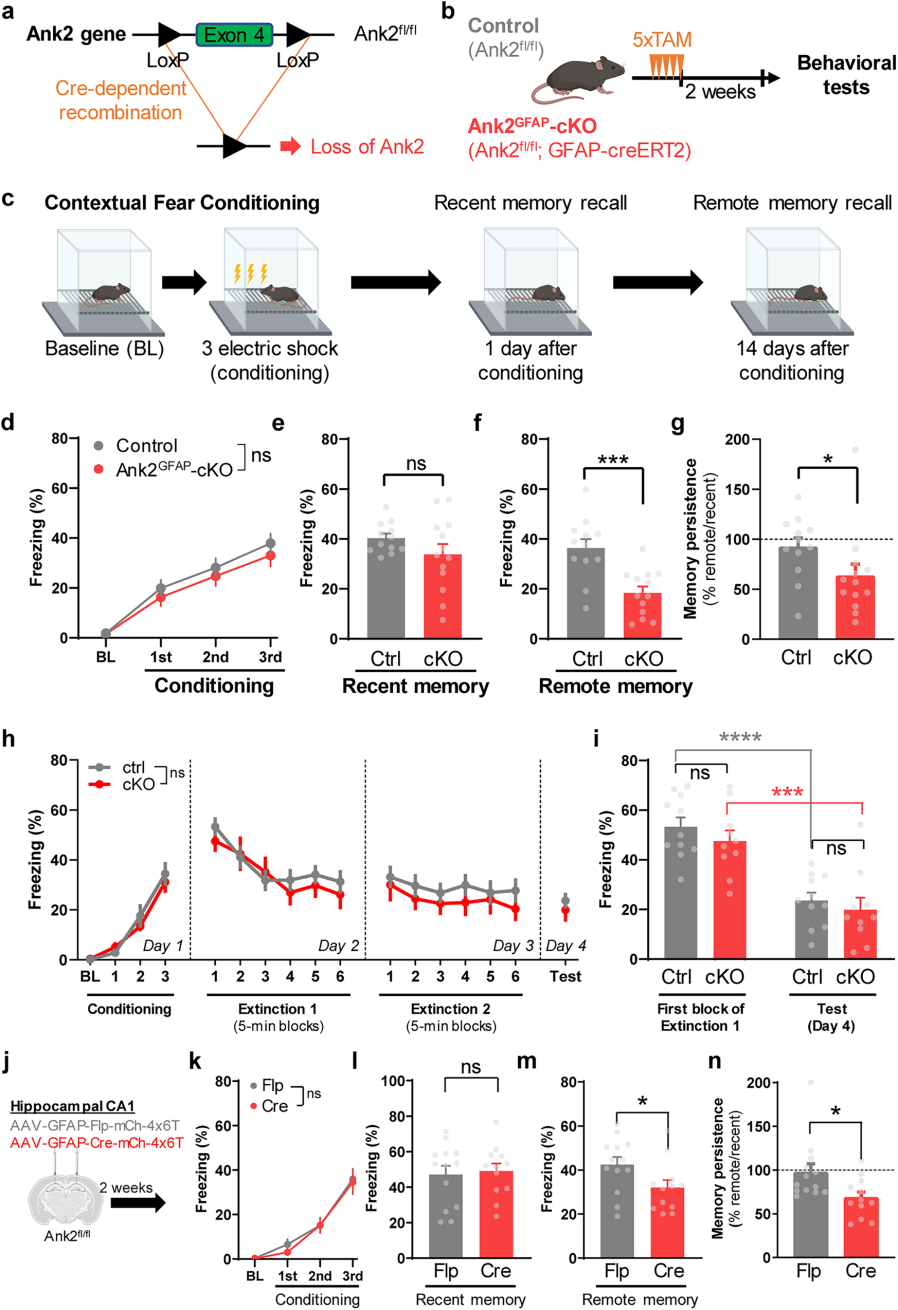
Astrocytic Ank2 deletion impairs memory persistence. **a** Cre-dependent deletion of Ank2 in Ank2 floxed (*Ank2*^*fl/fl*^) mice. **b** Generation of control and astrocyte-specific Ank2 conditional knockout (Ank2^GFAP^ cKO) mice. **c** Experimental design for contextual fear conditioning (three foot-shocks; 0.75 mA, 2 s each), assessing recent (Day 1) and remote (Day 14) memory. Freezing behavior in Control and Ank2^GFAP^ cKO mice during acquisition (**d**), recent memory test 1 day after acquisition (**e**), and remote memory test 14 days after acquisition (**f**; *P* = 0.0004). Acquisition data represent mean freezing across mice during the baseline (BL), 1st, 2nd, and 3rd shock periods in each group. Control, *n* = 12 mice; Ank2^GFAP^ cKO, *n* = 13 mice. **g** Memory persistence, calculated as the ratio of remote to recent freezing shown in (**e**, **f**). (*P* = 0.016). **h** Contextual fear extinction paradigm. Freezing measured during acquisition (Day 1), two extinction sessions (Days 2–3), and a probe test (Day 4) in Control and Ank2^GFAP^ cKO mice. Control, *n* = 11 mice; Ank2^GFAP^ cKO, *n* = 10 mice. **i** Comparison of freezing between the first 5-min block of extinction (Day 1) and the probe test (Day 4) from (**h**) (Control, *P* < 0.0001; Ank2^GFAP^ cKO, *P* = 0.0007). **j** Bilateral hippocampal astrocyte-specific Ank2 deletion. Freezing behavior during acquisition (**k**), recent memory test (**l**), and remote memory test (**m**; *P* = 0.0422). **n** Memory persistence in mice with hippocampal astrocytic Ank2 deletion (*P* = 0.0135). Flp group, *n* = 13 mice; Cre group, *n* = 12 mice. Only male mice were used for behavioral experiments. Data are mean ± SEM. *P < 0.05, ***P < 0.001, ****P < 0.0001; ns, not significant. Two-way repeated measures ANOVA (**d**, **h**, **k**), unpaired *t* test (**e**, **f**, **l**, **m**), Mann–Whitney test (**g**, **n**), and *t* tests for (**i**): paired within groups, unpaired between groups. All statistical tests were two-sided. Detailed statistics are provided in Supplementary Data 1. Panels **b**, **c**, **j** were created in BioRender. Koh, W. (2026) https://BioRender.com/4hltk5u. Source data are provided as a Source Data file.

To investigate the cognitive role of astrocytic Ank2, we generated transgenic mice with astrocyte-specific deletion of Ank2. We crossed Ank2 floxed mice^21^, which carry loxP sites flanking exon 4 of the *Ank2* gene and allow the deletion of both 440 and 220 kDa Ank2 isoforms, with astrocyte-specific Cre recombinase-expressing mice, producing *Ank2*^*fl/fl*^ and *Ank2*^*fl/fl*^;GFAP-CreER^T2^ mice (Fig. 1a). Given the broad expression of *Ank2* throughout the body, including in the heart, thymus, and other tissues^11,28^, we used the GFAP-CreER^T2^ line to induce tamoxifen-dependent Cre expression predominantly in brain astrocytes^29^, thereby reducing peripheral recombination compared to Aldh1l1-CreER^T2^ line^30^. Tamoxifen (100 mg/kg per day, 5 consecutive days) was administered to generate control (ctrl) and astrocyte-specific Ank2 conditional knockout (Ank2^GFAP^ cKO) mice (Fig. 1b).

We conducted a series of behavioral experiments to assess the effects of astrocytic Ank2 deletion on locomotion, anxiety, sociability, repetitive behaviors, and learning (Fig. 1, Supplementary Fig. 1, 2). In the open field test, there was no significant difference between control and Ank2^GFAP^ cKO mice in total distance traveled, time spent in the center zone, the number of entries into the center, or the number of fecal boli (Supplementary Fig. 1a–d), showing that basal locomotor activity and anxiety levels were unaffected. Assessments of ASD-related behaviors, including sociability, social novelty preference, and repetitive behaviors in the marble burying test, revealed no significant alterations in Ank2^GFAP^ cKO mice (Supplementary Fig. 1e–l). These findings indicate that astrocyte-specific deletion of Ank2 in adulthood does not impact basal locomotion, anxiety-like behaviors, sociability, or repetitive behaviors.

Next, we evaluated learning and memory using contextual fear conditioning (Fig. 1c–g). During the acquisition phase, both control and Ank2^GFAP^ cKO mice exhibited similar responses to three electric shocks (Fig. 1d), indicating normal shock perception and immediate fear response. One day post-conditioning, memory recall (recent memory) showed no significant difference between groups (Fig. 1e). However, when remote memory recall was assessed 14 days post-conditioning, Ank2^GFAP^ cKO mice exhibited significantly reduced freezing behavior compared to controls (Fig. 1f), suggesting impaired remote memory. Memory persistence, calculated as the ratio of freezing on day 14 to freezing on day 1, was also significantly reduced in Ank2^GFAP^ cKO mice (Fig. 1g).

Reduced remote memory recall in Ank2^GFAP^ cKO mice may result from accelerated extinction caused by context exposure during recent memory recall without shock. To test this possibility, we conducted an extinction experiment in which mice were re-exposed to the conditioning context for 30 min per day over two consecutive days without receiving any shocks, followed by a 5-min probe test on the fourth day (Fig. 1h). Both control and Ank2^GFAP^ cKO mice exhibited a gradual decrease in freezing behavior, indicative of normal extinction learning. Importantly, no significant differences were observed between the groups in the extinction test (Fig. 1i), indicating that accelerated extinction did not account for the remote memory impairment. We next considered whether circadian rhythm disruptions might affect memory consolidation, but monitoring of circadian activity and metabolic rhythms likewise revealed no significant differences between control and Ank2^GFAP^ cKO mice (Supplementary Fig. 2a–h). These findings indicate that the remote memory deficit is unlikely to result from circadian disturbances. Taken together, the results suggest that impaired remote memory in Ank2^GFAP^ cKO mice does not stem from accelerated extinction or circadian disruption, but rather reflects a specific deficit in the formation or retention of remote memory. To determine whether this impairment is specific to remote fear memory or extends to other types of long-term memory, we also performed an object location memory test (Supplementary Fig. 1m, n). Because object location memory is typically retained for up to two days^31^, we assessed memory at this interval. Unlike control mice, Ank2^GFAP^ cKO mice failed to discriminate the location change of the object, showing that astrocytic Ank2 contributes not only to remote fear memory but also to other forms of remote memory. Nevertheless, to investigate long-term memory more reliably, we continued to use contextual fear conditioning, which is more robustly retained over extended periods. Collectively, these results highlight a broad role for astrocytic Ank2 in supporting remote memory persistence.

The hippocampus is essential for contextual fear memory^32^, while also contributing to object location memory^33^. To test whether the remote memory deficit in Ank2^GFAP^ cKO mice results specifically from the loss of astrocytic Ank2 in the hippocampus, we bilaterally injected an adeno-associated virus (AAV) carrying Cre recombinase under the GFAP promoter (AAV-GfaABC1D-NLS-Cre-mCh-4x6T)^34^ into the CA1 region of *Ank2*^*fl/fl*^ mice. This construct contains a 4x6T miRNA targeting cassette that markedly enhances astrocyte specificity^34^, which was reproduced (Supplementary Fig. 3a, b). As a control, we used an AAV expressing Flp recombinase (AAV-GfaABC1D-NLS-Flp-mCh-4x6T). Consistent with our earlier findings, mice with hippocampal astrocyte-specific Ank2 deletion displayed intact recent memory but impaired remote memory (Fig. 1j–n). These results demonstrate that Ank2 in hippocampal astrocytes is required for remote memory formation, highlighting its crucial role in memory persistence.

### Astrocytic Ank2 deletion leads to simplified morphology of hippocampal astrocytes

**Fig. 2:**
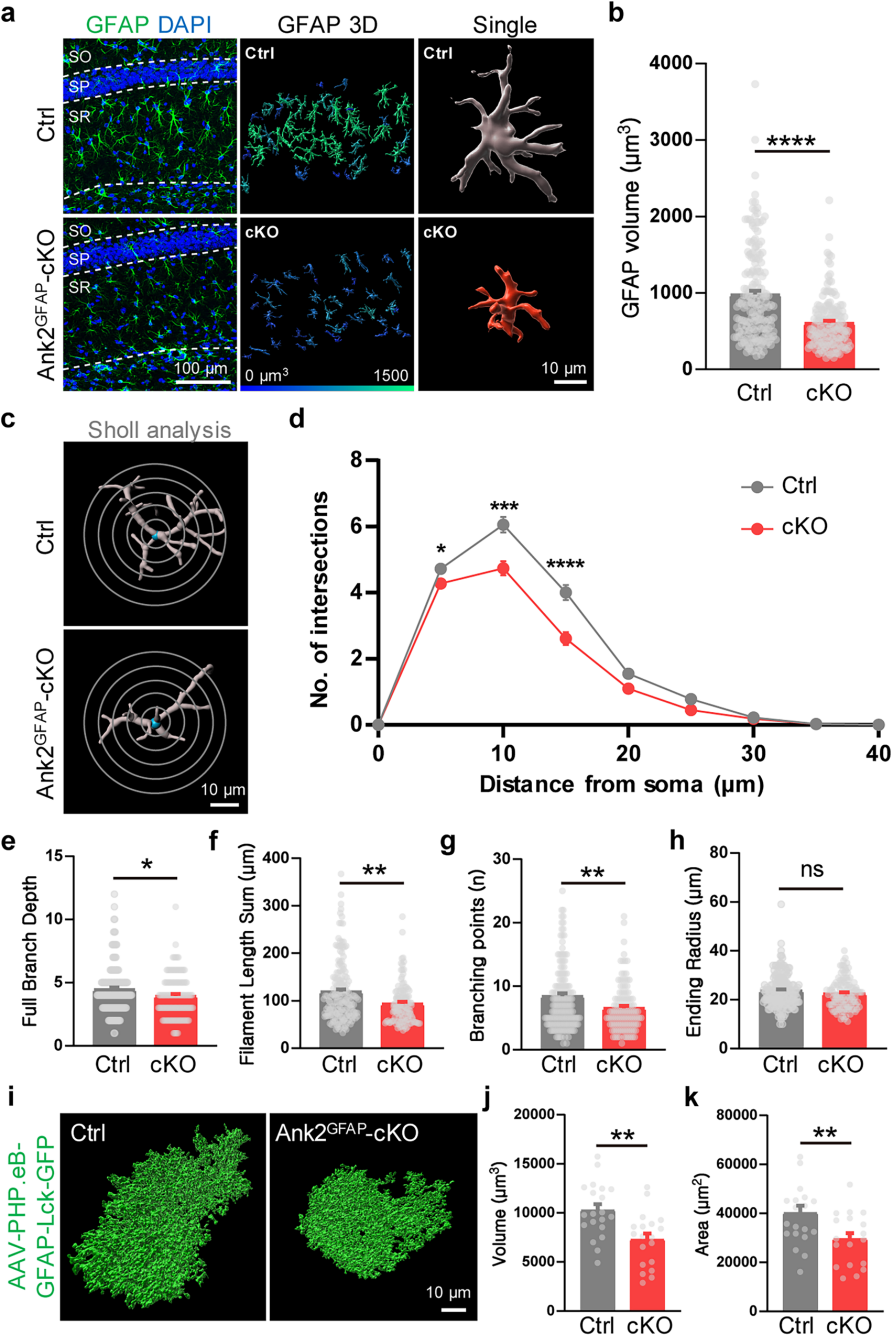
Deletion of Ank2 in astrocytes reduces astrocyte volume and morphological complexity. **a** Measurement of hippocampal astrocyte volume using GFAP staining and 3D reconstruction. **b** Comparison of GFAP-stained astrocyte volume between control and Ank2^GFAP^ cKO mice (*P* < 0.0001). Control group, *n* = 172 astrocytes from 2 mice; Ank2^GFAP^ cKO group, *n* = 147 astrocytes from 3 mice. **c** Sholl analysis of astrocytic structural complexity. Quantification of intersections (**d**; 5 μm, *P* = 0.0372; 10 μm, *P* = 0.0003; 15 μm, *P* < 0.0001), full branch depth (**e**; *P* = 0.0337), total filament length (**f**; *P* = 0.0013), number of branching points (**g**; *P* = 0.0106), and ending radius (**h**). Control group, *n* = 162 astrocytes; Ank2^GFAP^ cKO group, *n* = 113 astrocytes. **i** Visualization of whole astrocyte morphology with Lck-GFP expression in control and Ank2^GFAP^ cKO mice. **j** Quantification of astrocyte volume (*P* = 0.0019). Control group, *n* = 20 astrocytes from 4 mice; Ank2^GFAP^ cKO group, *n* = 19 astrocytes from 3 mice. **k** Surface area of hippocampal astrocytes (*P* = 0.0096). Control group, *n* = 20 astrocytes; Ank2^GFAP^ cKO group, *n* = 19 astrocytes. Data are mean ± SEM. *P < 0.05, **P < 0.01, ***P < 0.001, ****P < 0.0001; ns not significant. Mann–Whitney test (**b**, **d**–**h**), unpaired *t* test (**j**, **k**). All statistical tests were two-sided. Detailed statistics are provided in Supplementary Data 1. Source data are provided as a Source Data file.

Following our findings on the role of Ank2 in hippocampal astrocytes on remote memory formation, we extended our investigation to examine the corresponding changes in hippocampal astrocytes. In Ank2^GFAP^ cKO mice, hippocampal Ank2 expression was reduced by about 50% (Supplementary Fig. 3c, d), reflecting loss in astrocytes, while the remaining signal likely represents neuronal Ank2, as previously reported in *Ank2*^*fl/fl*^;*Emx1-Cre* mice^21^. Given that Ank2 has been implicated in cellular morphology^11,28^, we tested whether its deletion alters astrocyte structure (Fig. 2). GFAP staining revealed a significant reduction in astrocytic volume (Fig. 2a, b). Sholl analysis further showed fewer intersections, shorter total branch length, and fewer branching points, with a mild reduction in ending radius (Fig. 2c–h). Together, these data indicate reduced structural complexity of hippocampal astrocytes.

GFAP labels only main branches and accounts for about 15% of total astrocytic volume^35^. To visualize full morphology of astrocytes, we expressed membrane-bound GFP in sparse astrocytes via retro-orbital injection of AAV-PHP.eB-GFAP-Lck-GFP^36^ (Fig. 2i–k). In Ank2^GFAP^ cKO mice, both astrocytic volume and surface area were reduced, together with a loss of GFAP-positive branches. Ezrin, a marker of perisynaptic processes, was also slightly decreased (Supplementary Fig. 3e, f), while the number of astrocytes was unchanged (Supplementary Fig. 3g, h). These results show that Ank2 is required to maintain astrocytic morphology and suggest that its loss may compromise learning and memory, particularly remote memory.

### Ank2-deleted astrocytes maintain normal passive conductance and form astrocyte syncytium

While Ank2 is a structural protein, it also regulates the expression and membrane localization of ion channels and transporters^11^. Astrocytes express numerous ion channels and transporters that contribute to their electrophysiological properties^37^, including passive conductance, and enable the formation of astrocyte syncytium through gap junctions. We tested whether astrocytic Ank2 deletion alters these properties.

**Fig. 3:**
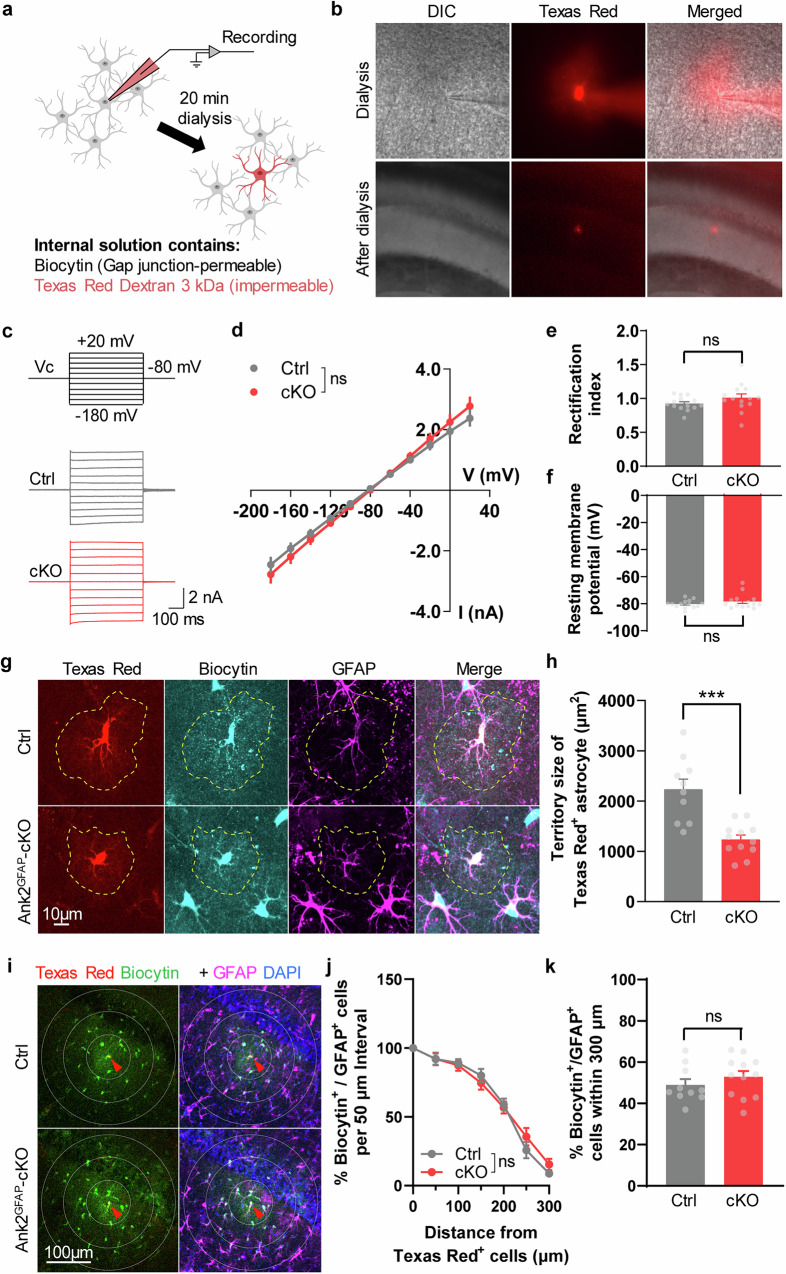
Ank2-deleted astrocytes maintain normal passive conductance and form an astrocyte syncytium. **a** Whole-cell patch-clamp configuration with Texas Red Dextran (3 kDa, gap junction-impermeable) and biocytin (gap junction-permeable) to visualize individual astrocytes and syncytium coupling. **b** Example image of a single Texas Red-labeled astrocyte. Passive conductance recorded from hippocampal astrocytes, shown as representative traces (**c**) and I-V curves (**d**), rectification index (**e**), and resting membrane potential (**f**). I-V curves, rectification index, and resting membrane potential were obtained from the same recorded astrocytes. Control, n = 14 astrocytes from 4 mice; Ank2^GFAP^ cKO, *n* = 14 astrocytes from 3 mice. **g** Validation of dye diffusion by immunohistochemistry for biocytin and GFAP. **h** Astrocyte territory size measured from Texas Red^+^ cells. Control group, *n* = 10 astrocytes from 3 mice; Ank2^GFAP^ cKO group, *n* = 12 astrocytes from 3 mice. **i** Representative images of biocytin diffusion through gap junctions. Quantification of biocytin spread: percentage of biocytin^+^ astrocytes at 50 μm intervals (**j**) and within 300 μm radius (**k**). Control group, *n* = 10 slices; Ank2^GFAP^ cKO group, *n* = 12 slices. Data are mean ± SEM. ***P < 0.001; ns, not significant. Unpaired *t* test (**d**, **e**, **h**, **j**, **k**) and Mann–Whitney test (**d**, **f**, **j**). All statistical tests were two-sided. Detailed statistics are provided in Supplementary Data 1. (a) was created in BioRender. Koh, W. (2026) https://BioRender.com/4hltk5u. Source data are provided as a Source Data file.

Whole-cell patch-clamp recordings were performed in hippocampal astrocytes using pipettes loaded with Texas Red Dextran 3 kDa (gap junction-impermeable) and biocytin (gap junction-permeable) (Fig. 3a, b). Passive conductance, rectification index, and resting membrane potential did not differ between control and Ank2^GFAP^ cKO astrocytes (Fig. 3c–f). After 20 min of dye diffusion, Texas Red remained confined to the patched cell, allowing analysis of astrocytic territory, which was reduced in Ank2^GFAP^ cKO mice (Fig. 3g, h). Biocytin spread through gap junctions, however, was similar between groups (Fig. 3i–k). Thus, Ank2 deletion does not affect ion channels or gap junctions underlying passive conductance, resting potential, or syncytium formation.

We next examined whether astrocytic Ank2 deletion influences CA1 pyramidal neurons (Supplementary Fig. 4). Whole-cell recordings revealed no changes in intrinsic excitability or resting potential (Supplementary Fig. 4a–c). Spontaneous EPSCs (at -70 mV) and IPSCs (at 0 mV) also showed no differences in amplitude or frequency (Supplementary Fig. 4d–i). Field EPSP (fEPSP) recordings further revealed normal input-output relationships and paired-pulse ratios (Supplementary Fig. 5a, b). These findings indicate that reduced astrocyte volume and complexity in Ank2^GFAP^ cKO mice do not significantly alter basal neuronal excitability or synaptic transmission.

### Astrocytic Ank2 deletion impairs LTP maintenance and reduces learning-dependent astrocytic contacts with engram neurons

**Fig. 4:**
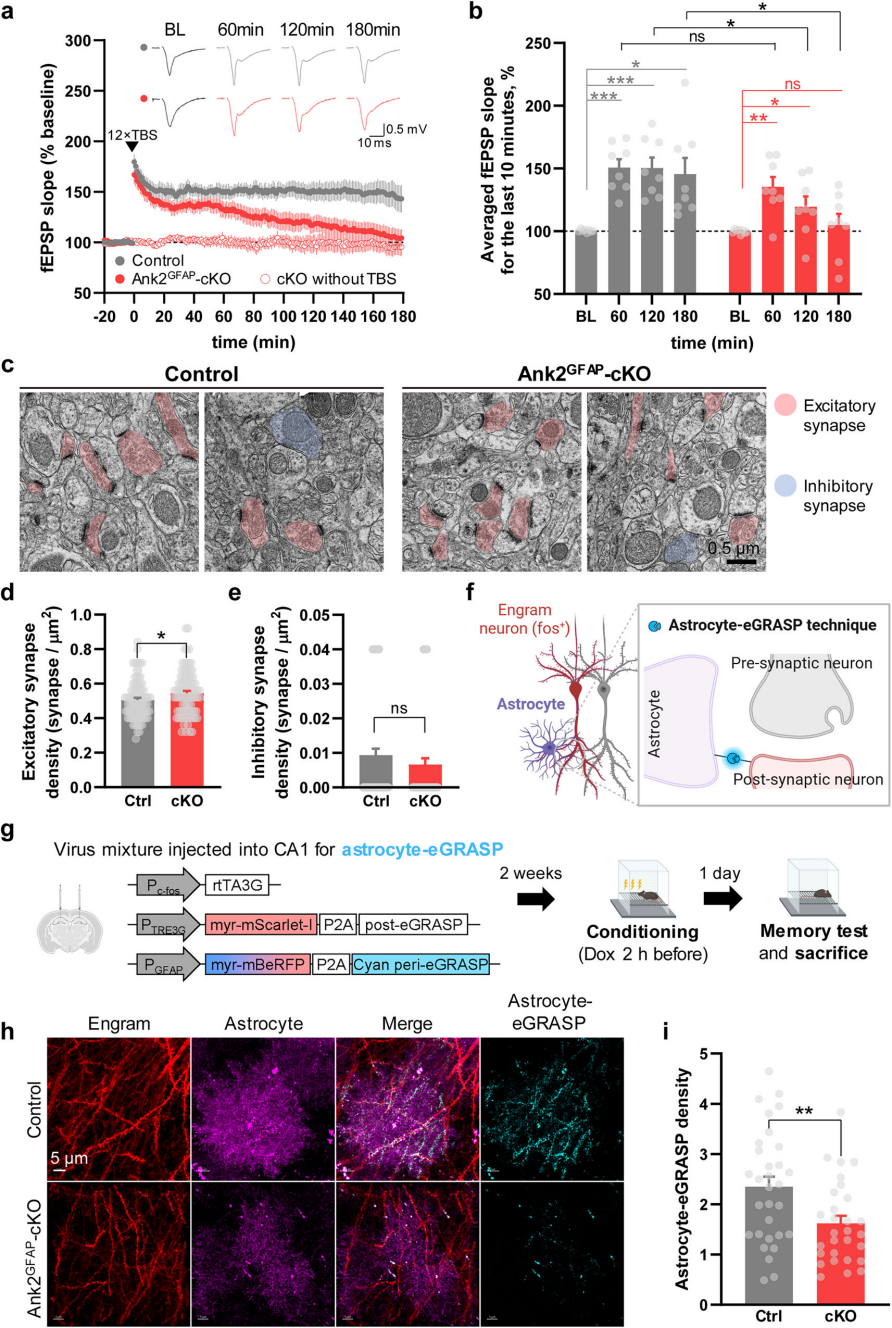
Astrocytic Ank2 deletion impairs LTP maintenance and reduces learning-dependent astrocytic contacts with engram neurons in the hippocampus. **a** LTP induced by 12-theta burst stimulation (12×TBS) in hippocampal slices, shown as mean normalized fEPSP slopes recorded for 180 min after stimulation. **b** Averaged fEPSP slope changes at 60, 120, and 180 min after 12×TBS from (**a**) (Control: BL vs 60 min, *P* = 0.0001; BL vs 120 min, *P* = 0.0006; BL vs 180 min, *P* = 0.0101. Ank2^GFAP^ cKO: BL vs 60 min, *P* = 0.0022; BL vs 120 min, *P* = 0.0421. Control vs Ank2^GFAP^ cKO: 120 min, *P* = 0.0194; 180 min, *P* = 0.0211). Control group, *n* = 8 slices from 3 mice; Ank2^GFAP^ cKO group, *n* = 8 slices from 3 mice; Ank2^GFAP^ cKO without TBS group, *n* = 11 slices from 2 mice. **c** Representative TEM images showing excitatory and inhibitory synapses in hippocampal CA1. Quantification of excitatory (**d**; *P* = 0.0336) and inhibitory (**e**) synapse density. Control group, *n* = 90 images from 3 mice; Ank2^GFAP^ cKO group, *n* = 90 images from 3 mice. **f** Astrocyte-eGRASP techniques. **g** Viral components used to visualize astrocytic contacts on engram neurons and behavioral scheme for engram neuron labeling. **h** Representative astrocyte-eGRASP images in hippocampal CA1 from control and Ank2^GFAP^ cKO mice. **i** Quantification of astrocyte-eGRASP signal density (*P* = 0.0056). Control group, *n* = 31 astrocytes from 5 mice; Ank2^GFAP^ cKO group, *n* = 29 astrocytes from 5 mice. Data are mean ± SEM. *P < 0.05, **P < 0.01, ***P < 0.001; ns, not significant. Paired *t* test and unpaired *t* test (**b**), unpaired *t* test (**d**, **e**, **i**). All statistical tests were two-sided. Detailed statistics are provided in Supplementary Data 1. Panels **f**, **g** were created in BioRender. Koh, W. (2026) https://BioRender.com/4hltk5u. Source data are provided as a Source Data file.

Given that Ank2 has been reported to be expressed in astrocytic leaflets and its deletion reduces astrocyte volume, we investigated whether this morphological change affects synaptic plasticity, particularly in the maintenance of long-term potentiation (LTP), a key cellular mechanism for memory stabilization. To explore this, we performed fEPSP recordings to examine both early-phase LTP (E-LTP, ~1 h) and late-phase LTP (L-LTP, up to 3 h) in the hippocampal Schaffer collateral pathway, delivering 12 theta-burst stimulations (12×TBS) to induce BDNF-dependent L-LTP^38^. In control mice, LTP was successfully induced and maintained for 3 h (Fig. 4a, b). In Ank2^GFAP^ cKO mice, E-LTP was intact, but potentiation gradually decayed, indicating impaired L-LTP (Fig. 4a, b). Baseline fEPSPs remained stable without TBS (Fig. 4a, unfilled red circle), thereby excluding slice deterioration as a contributing factor. These results suggest that astrocytic Ank2 is required for BDNF-dependent maintenance of synaptic potentiation, a mechanism critical for long-term memory.

Astrocytic leaflets enwrap active synapses, supporting their stabilization and maintenance^8^, which may contribute to remote memory consolidation. We wondered whether astrocytic Ank2 loss alters astrocytic contacts on engram neurons after memory acquisition^9,10^. First, using transmission electron microscopy (TEM), we quantified the density of excitatory and inhibitory synapses in hippocampal CA1. Inhibitory synapse density was unchanged, whereas excitatory synapse density was modestly but significantly increased in Ank2^GFAP^ cKO mice compared to controls (Fig. 4c–e). Given that basal synaptic transmission was unaltered in Ank2^GFAP^ cKO slices, this increase likely reflects a compensatory adjustment to preserve synaptic transmission despite reduced astrocytic volume and complexity. We then investigated learning-dependent interactions using astrocyte-eGRASP^10^ (Fig. 4f), which enables direct visualization of astrocytic contacts with engram neurons. Astrocytes were labeled with AAV-GfaABC1D-myr-mBeRFP-P2A-Cyan peri-eGRASP, and engram neurons were labeled with AAV-c-fos-rtTA3G and AAV-TRE3G-myr-mScarlet-I-P2A-post-eGRASP, induced by intraperitoneal doxycycline injection 2 h before contextual fear conditioning (Fig. 4g–i). Although recent memory did not differ between groups (Fig. 1 and supplementary Fig. 5c), astrocyte-eGRASP puncta density was significantly reduced in Ank2^GFAP^ cKO mice (Fig. 4h, i). These results indicate that Ank2 deletion diminishes learning-dependent astrocytic contacts with engram neurons, potentially compromising synaptic stability and memory persistence.

### Ank2 binds to IP3R2 that regulates astrocytic Ca^2+^ signaling and BDNF-dependent morphogenesis

**Fig. 5:**
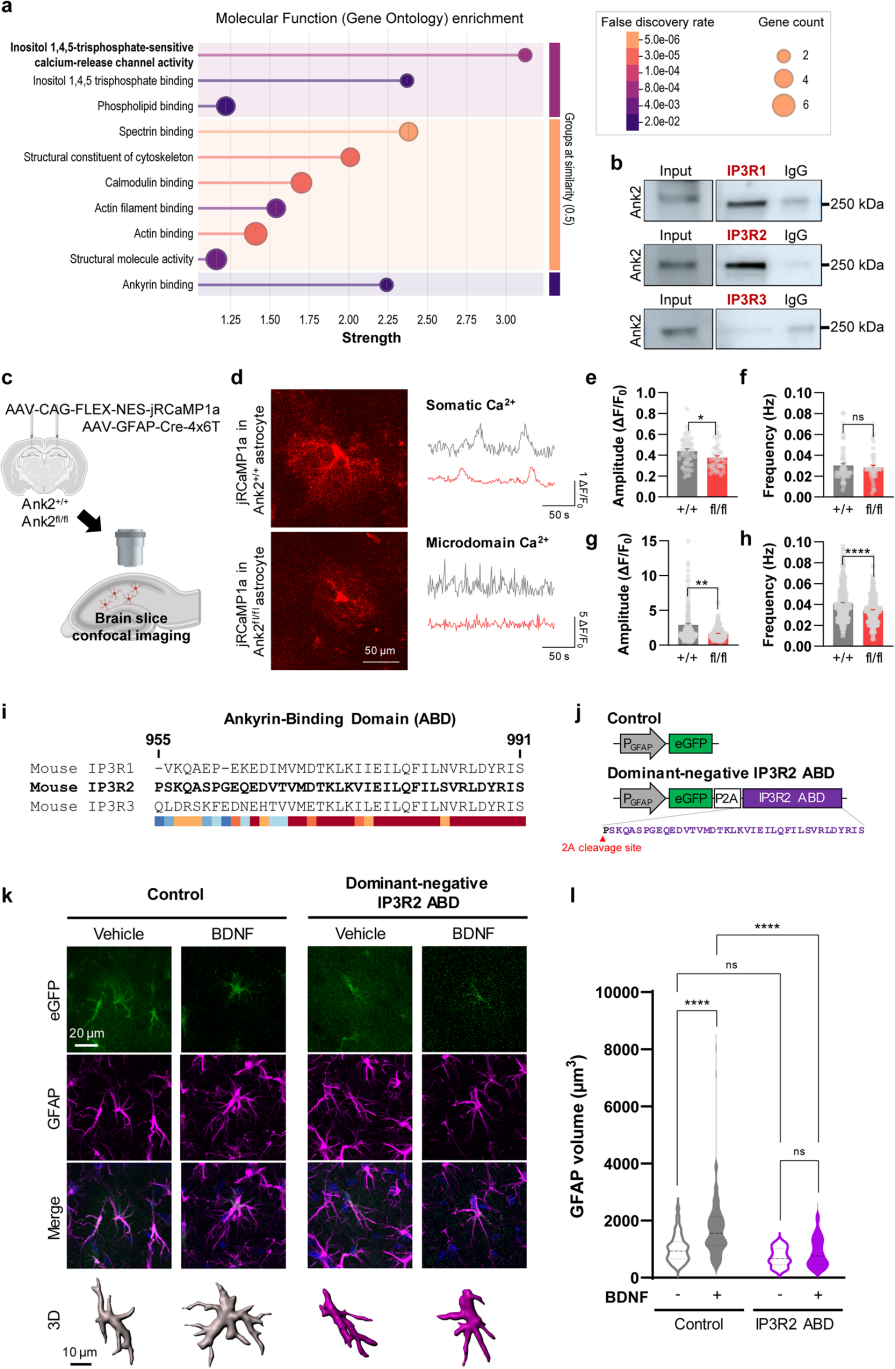
Ank2 interacts with IP3R2 and regulates astrocytic Ca2+ signaling and BDNF-dependent morphogenesis. **a** STRING analysis of Ank2 molecular function enrichment showing associations with inositol 1,4,5-triphosphate-sensitive calcium-release channel activity and cytoskeletal proteins. **b** Co-immunoprecipitation (Co-IP) of Ank2 with IP3R1, IP3R2, and IP3R3 from mouse brain lysates. Results were reproduced in three independent experiments. **c** Experimental scheme for the expression of jRCaMP1a (Ca^2+^ indicator) with Cre-dependent deletion of Ank2. **d** Representative images and traces corresponding to the quantifications shown in (**e**–**h**). **d**, left Representative images of jRCaMP1a-expressing hippocampal astrocytes from *Ank2*^+/+^ (top) and *Ank2*^*fl/fl*^ (bottom) mice. **d**, right Representative traces of jRCaMP1a fluorescence from soma (top) and microdomains (bottom) in *Ank2*^+/+^ (gray) and *Ank2*^*fl/fl*^ (red) astrocytes. Quantification of ΔF/F_0_ jRCaMP1a signals in soma (**e**, *P* = 0.0361; **f**) and microdomains (**g**, *P* = 0.0037; **h**, *P* < 0.0001) of *Ank2*^+/+^ and *Ank2*^*fl/fl*^ astrocytes. Control group, *n* = 51 soma and *n* = 433 microdomains from 3 mice; Ank2^GFAP^ cKO group, *n* = 40 soma and *n* = 289 microdomains from 2 mice. **i** Alignment of ankyrin-binding domains (ABD, residues 955-991) conserved across IP3R isoforms. **j** Schematic of AAV constructs for expression of control (GFAP-eGFP) and dominant-negative IP3R2 ABD (GFAP-eGFP-P2A-IP3R2 ABD). **k** Representative images of GFAP immunostaining and eGFP signals in hippocampal tissue following vehicle or BDNF infusion (100 ng/0.4 μl). **l** Quantification of GFAP volume in astrocytes expressing control or IP3R2 ABD constructs following vehicle or BDNF infusion (construct × treatment interaction, *P* = 0.036; control+vehicle vs. control+BDNF, *P* < 0.0001; control+BDNF vs. IP3R2 ABD+vehicle, *P* < 0.0001; control+BDNF vs. IP3R2 ABD + BDNF, *P* < 0.0001). Control+vehicle: *n* = 93 astrocytes from 3 mice; control+BDNF: *n* = 200 astrocytes from 6 mice; IP3R2 ABD+vehicle: *n* = 13 astrocytes from 3 mice; IP3R2 ABD + BDNF: *n* = 97 astrocytes from 6 mice. Data are mean ± SEM. *P < 0.05, **P < 0.01, ****P < 0.0001; ns, not significant. Mann–Whitney test (**e**–**h**) and two-way ANOVA with Tukey’s multiple comparisons test (**l**). All statistical tests were two-sided. Detailed statistics are provided in Supplementary Data 1. Panel **c** was created in BioRender. Koh, W. (2026) https://BioRender.com/4hltk5u. Source data are provided as a Source Data file.

We next investigated the molecular mechanisms underlying reduced astrocytic volume, impaired BDNF-dependent L-LTP, and diminished astrocytic contacts with engram neurons in Ank2^GFAP^ cKO mice. BDNF is essential for synaptic plasticity and memory^39^ and also promotes astrocyte morphogenesis through the truncated TrkB.T1 receptor^40,41^. In astrocytes, TrkB.T1 activation triggers endoplasmic reticulum (ER) Ca^2+^ release through inositol 1,4,5-triphosphate receptors (IP3Rs)^42^. Given that IP3R2 is the predominant isoform in astrocytes, we hypothesized that Ank2 supports IP3R2 function in mediating BDNF signaling. STRING analysis of Ank2 molecular function revealed strong enrichment for inositol 1,4,5-triphosphate-sensitive calcium-release channel activity and high association scores with cytoskeletal proteins (Fig. 5a), suggesting a functional link to IP3Rs. Co-immunoprecipitation from brain lysates confirmed Ank2 binding to IP3R1, as previously reported^43^, and additionally to IP3R2, with minimal binding to IP3R3 (Fig. 5b). IP3R2 protein levels were significantly reduced in hippocampal astrocytes of Ank2 cKO mice, indicating that Ank2 contributes to the expression of IP3R2 (Supplementary Fig. 6a, b).

Given the importance of IP3R2 for astrocytic Ca^2+^ signaling^44^, we next monitored spontaneous Ca^2+^ activity in Ank2-deleted astrocytes. AAV-GfaABC1D-Cre-4x6T was injected into the hippocampal CA1 of *Ank2*^+/+^ or *Ank2*^*fl/fl*^ mice to delete Ank2 in astrocytes, together with AAV-CAG-FLEX-NES-jRCaMP1a to measure Ca^2+^ signals^45^ (Fig. 5c). Ank2 deletion significantly reduced somatic Ca^2+^ amplitude as well as the amplitude and frequency of Ca^2+^ microdomains (Fig. 5c–h). These phenotypes closely resembled those in IP3R2 knockout mice, indicating that Ank2 supports IP3R2 functions in astrocytes.

We then tested whether IP3R2 contributes to BDNF-dependent astrocyte morphogenesis. To this end, IP3R2 was knocked down with shRNA (AAV-GFAP-mCherry-shITPR2), and acute hippocampal slices were treated with 50 ng/ml BDNF. IP3R2 knockdown significantly reduced BDNF-induced GFAP intensity compared with control shRNA (AAV-GFAP-mCherry-shLuci) (Supplementary Fig. 6c–e). This finding suggests that IP3R2 contributes to BDNF-dependent astrocyte morphogenesis, yet it does not delineate the specific contribution of Ank2-IP3R2 interactions.

To specifically interrogate the functional relevance of the Ank2-IP3R2 interaction, we targeted the ankyrin-binding domain (ABD) located in residues 955-991, which is conserved among IP3Rs^46^. We expressed the IP3R2 ABD as a dominant-negative construct (AAV-GFAP-eGFP-P2A-IP3R2 ABD) in astrocytes (Fig. 5i–l, Supplementary Fig. 7a, b). Expression of IP3R2 ABD alone did not alter GFAP intensity (Supplementary Fig. 7a, b). We next asked whether expression of IP3R2 ABD alters BDNF-dependent astrocyte morphogenesis in vivo. Hippocampal infusion of BDNF (100 ng/0.4 μl) increased GFAP volume relative to vehicle (ACSF)-treated controls (Supplementary Fig. 7c–e). Consistent with this control (AAV-GFAP-eGFP) astrocytes exhibited a significant BDNF-dependent increase in GFAP volume, whereas this increase was not observed in astrocytes expressing IP3R2 ABD (Fig. 5k, l; two-way ANOVA, construct × BDNF effect interaction, *P* = 0.036).

Together, these results demonstrate that Ank2 supports IP3R2 function to mediate BDNF-dependent astrocyte morphogenesis. Given the established role of IP3R2 in L-LTP and remote memory^2^, Ank2 emerges as a key regulator of astrocytic structural plasticity that underlies memory persistence.

### Ank2 is essential for BDNF-dependent astrocyte morphogenesis

**Fig. 6:**
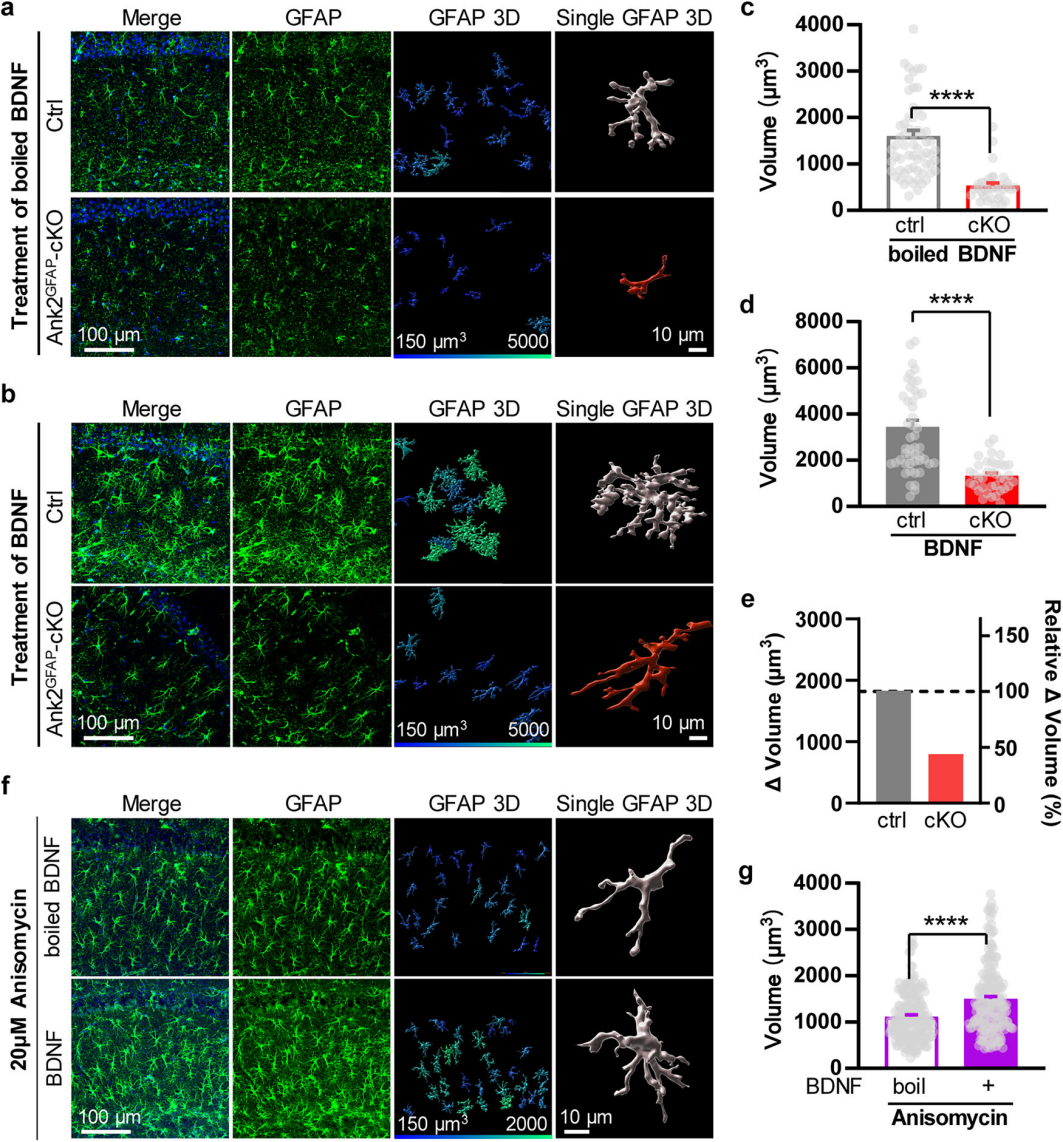
Ank2-deleted astrocytes exhibit impaired BDNF-dependent astrocyte morphogenesis. Representative images of (**a**) boiled (inactivated by denaturation) BDNF or **b** active BDNF-induced astrocyte morphogenesis in control and Ank2^GFAP^ cKO mice, visualized using GFAP staining and volume reconstruction. Quantification of GFAP volume in control and Ank2^GFAP^ cKO mice following treatment with either (**c**) boiled (*P* < 0.0001) or **d** active BDNF (*P* < 0.0001) for 30 min, followed by an additional 30-min incubation in aCSF. Control+boiled BDNF group, *n* = 65 astrocytes from 2 mice; cKO+boiled BDNF group, *n* = 34 astrocytes from 2 mice. Control+BDNF group, *n* = 50 astrocytes from 2 mice; cKO+BDNF group, *n* = 37 astrocytes from 2 mice (two-way ANOVA, genotype × BDNF effect, *P* = 0.0084). **e** BDNF-induced astrocyte volume change (Δ Volume), calculated as the difference between astrocyte volume after BDNF treatment and after boiled BDNF treatment (left axis), and relative Δ Volume normalized to the control group (right axis) in control and Ank2^GFAP^ cKO astrocytes. **f** Representative images of BDNF-induced astrocyte morphogenesis in the presence of 20 μM anisomycin. **g** Quantification of GFAP volume following boiled BDNF or active BDNF treatment for 30 min, followed by an additional 30-min incubation in aCSF, in the presence of 20 μM anisomycin (*P* < 0.0001). Boiled BDNF group, *n* = 187 astrocytes from 2 mice; BDNF group, *n* = 188 astrocytes from 2 mice. Data are mean ± SEM. ****P < 0.0001. Mann–Whitney test (**c**, **d**, **g**). All statistical tests were two-sided. Detailed statistics are provided in Supplementary Data 1. Source data are provided as a Source Data file.

Having established the requirement of IP3R2 in BDNF-dependent astrocytic morphogenesis, we next asked whether Ank2 itself is necessary for this process. Acute hippocampal slices were treated with BDNF (50 ng/ml, 30 min) or boiled, denatured BDNF as a control, followed by a 30-min incubation in aCSF to allow morphogenesis (Fig. 6). We then performed IHC to visualize GFAP and assess astrocyte morphological responses to BDNF. In control mice, BDNF treatment significantly increased the GFAP volume of hippocampal astrocytes (from 1602 to 3435 µm^3^), while in Ank2^GFAP^ cKO mice, BDNF produced a smaller increase in GFAP volume (from 531 to 1331 µm^3^) (Fig. 6a–d, two-way ANOVA, genotype × BDNF effect interaction, *P* = 0.0084). Consequently, the GFAP volume in Ank2^GFAP^ cKO mice was reduced by 61.3% compared to controls following BDNF treatment (Fig. 6e), demonstrating that Ank2 is critical for BDNF-dependent astrocyte morphogenesis.

In addition, we examined whether astrocyte morphogenesis could occur via cytoskeletal rearrangement in the absence of de novo protein synthesis. Acute brain slices from naïve mice were treated with BDNF in the presence of 20 μM anisomycin, a protein synthesis inhibitor. Remarkably, BDNF-induced morphogenesis was still observed despite inhibition of protein synthesis (Fig. 6f, g), showing that cytoskeletal rearrangement alone is sufficient to drive BDNF-dependent astrocyte morphogenesis.

Collectively, these results demonstrate that Ank2 is essential for BDNF-IP3R2-dependent astrocyte morphogenesis. This process can proceed independently of new protein synthesis, likely via cytoskeletal mechanisms such as protein kinase C-dependent remodeling^47^. Such remodeling may support the formation of astrocytic contacts with engram neurons for synaptic stabilization. Notably, hippocampal BDNF infusion enhances memory persistence even under protein synthesis inhibition^39^, suggesting that astrocytic morphogenesis may contribute to this effect in a protein synthesis-independent manner.

### Astrocytic Ank2 is necessary for BDNF-dependent memory persistence

**Fig. 7:**
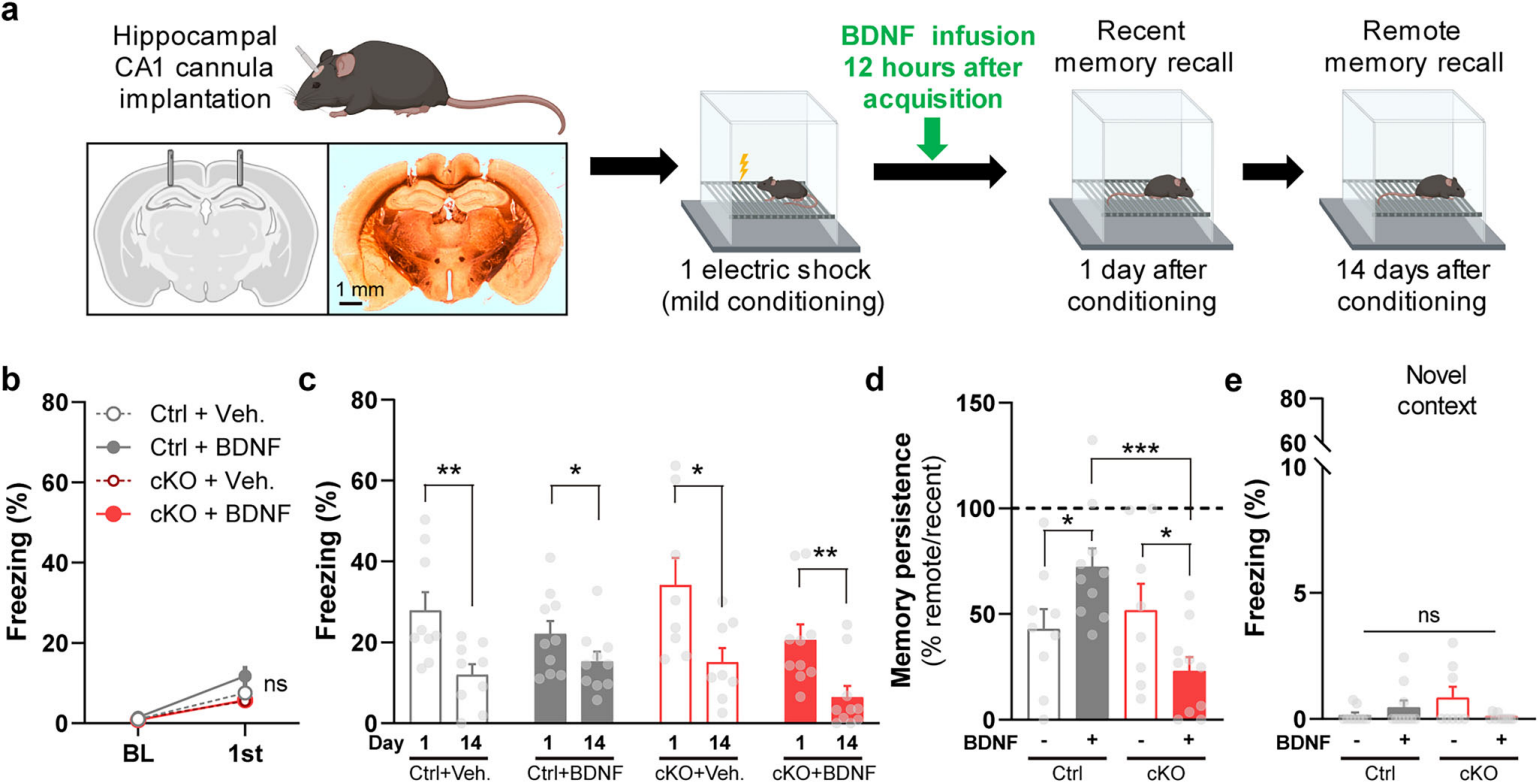
BDNF-induced memory persistence is reduced in astrocyte-specific Ank2-deleted mice. **a** Schematic diagram of contextual fear conditioning with BDNF infusion into the hippocampal CA1. Mild conditioning (one electric shock instead of three, 0.75 mA, 2 s) was used to assess BDNF-induced memory persistence. **b**–**e** Freezing behavior measured across different phases: **b** during acquisition, **c** in the recent and remote memory test (control+vehicle, *P* = 0.0022; control+BDNF, *P* = 0.0189; Ank2^GFAP^ cKO+vehicle, *P* = 0.0313; Ank2^GFAP^ cKO+BDNF, *P* = 0.0020). **d** Memory persistence in control mice with vehicle (aCSF) or BDNF (100 ng/0.4 μl/side) infusion, and in Ank2^GFAP^ cKO mice with BDNF infusion into hippocampal CA1 12 h after memory acquisition (two-way ANOVA, genotype × BDNF effect, *P* = 0.0036; control+BDNF vs. Ank2^GFAP^ cKO+BDNF, *P* = 0.0004; Ank2^GFAP^ cKO+vehicle vs. Ank2^GFAP^ cKO+BDNF, *P* = 0.0386). **e** Freezing behavior in the novel context test. Control+vehicle group, *n* = 9 mice; Control+BDNF group, *n* = 10 mice; Ank2^GFAP^ cKO+vehicle group, *n* = 8 mice; Ank2^GFAP^ cKO+BDNF group, *n* = 10 mice. Only male mice were used for behavioral experiments. Data are mean ± SEM. *P < 0.05, **P < 0.01, ***P < 0.001. ns, not significant. Two-way repeated measures ANOVA (**b**), paired *t* test (**c**, control+vehicle and control+BDNF), Wilcoxon test (**c**, Ank2^GFAP^ cKO+BDNF), two-way ANOVA with post hoc multiple comparisons (**d**), and Dunn’s multiple comparisons test (**e**). All statistical tests were two-sided. Detailed statistics are provided in Supplementary Data 1. Panel **a** was created in BioRender. Koh, W. (2026) https://BioRender.com/4hltk5u. Source data are provided as a Source Data file.

BDNF is crucial for both recent and remote memory formation, with a particularly important role in supporting memory persistence through its activity around 12 h after learning^39,48^. This critical time window prompted us to examine whether BDNF-dependent enhancement of memory persistence is impaired in Ank2^GFAP^ cKO mice (Fig. 7), which exhibit deficits in BDNF-dependent L-LTP and astrocyte morphogenesis.

To specifically assess whether BDNF-dependent astrocyte morphogenesis contributes to memory persistence, independent of endogenous BDNF synthesis^2^, we employed a previously established mild conditioning paradigm that induces minimal BDNF expression 12 h post-learning^39,48^ (Fig. 7a). This paradigm allowed us to specifically test whether extracellular BDNF, acting through astrocyte morphogenesis, enhances memory persistence. Following memory acquisition using this mild paradigm, we infused BDNF (100 ng/0.4 µL per side) into the hippocampus 12 h post-learning in both control and Ank2^GFAP^ cKO mice, and subsequently assessed recent and remote memory performance (Fig. 7b, c). In control mice, BDNF infusion significantly enhanced memory persistence, whereas Ank2^GFAP^ cKO mice failed to show this enhancement and instead exhibited reduced memory persistence despite receiving the same treatment (Fig. 7d; two-way ANOVA, genotype × BDNF effect interaction, *P* = 0.0036). To determine whether the enhanced freezing response was due to memory persistence rather than context-generalized fear, we exposed the mice to a novel context the following day and measured freezing behavior (Fig. 7e). The results confirmed that BDNF infusion did not induce generalized fear responses. These findings demonstrate that astrocytic Ank2 is necessary for BDNF-dependent enhancement of memory persistence. We next asked whether astrocytic BDNF signaling itself is sufficient to produce this effect.

### Activation of astrocytic TrkB.T1 signaling is sufficient to enhance memory persistence

Given the requirement of astrocytes for BDNF-induced enhancement of memory persistence 12 h after learning, we asked whether astrocytic TrkB.T1 signaling alone is sufficient to produce this effect. However, the lack of tools to precisely control local BDNF signaling has hindered such investigation. To overcome this limitation, we developed an optogenetic approach to selectively stimulate astrocytic TrkB.T1 signaling and assess its contribution to remote memory formation.

**Fig. 8:**
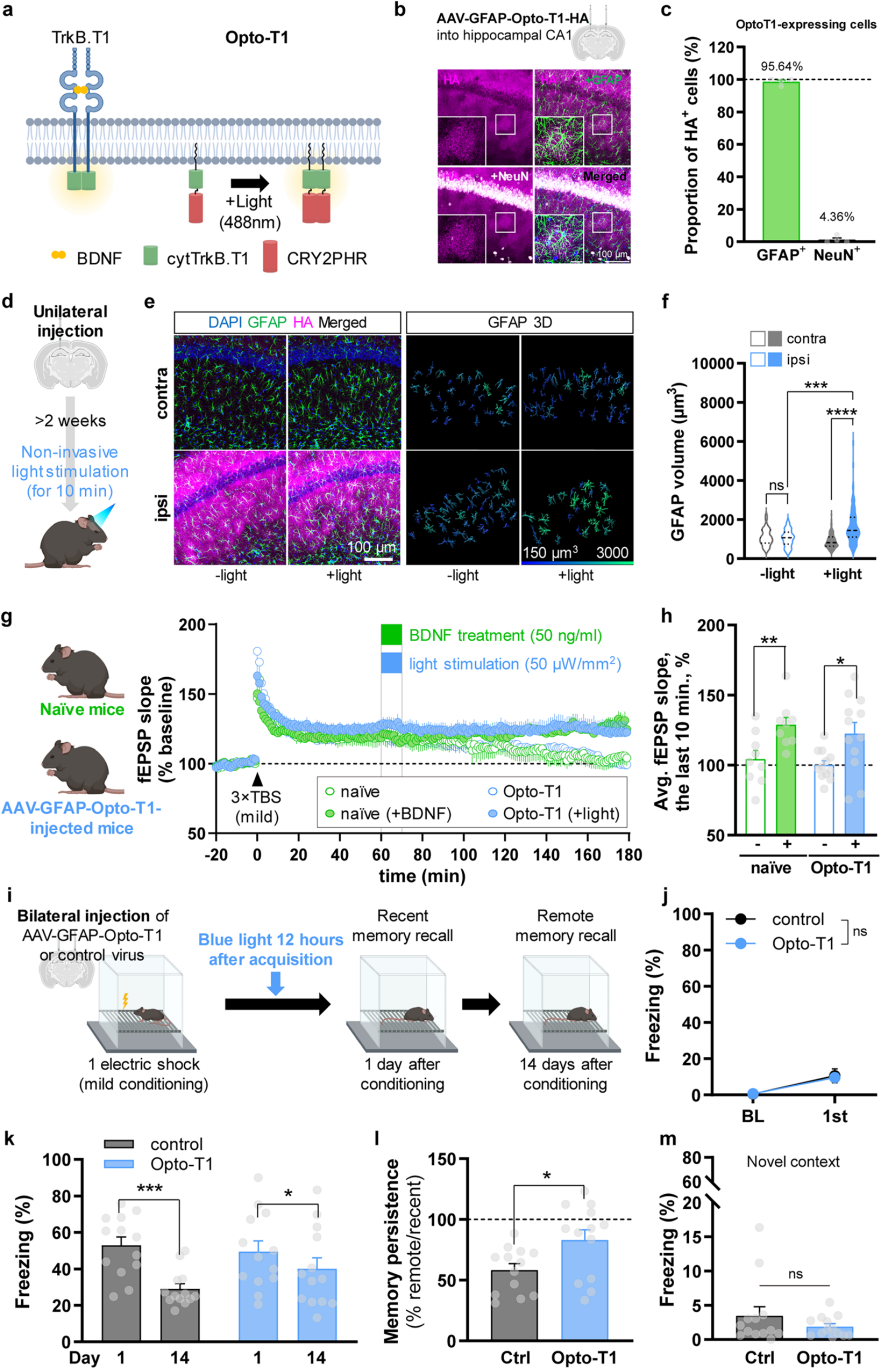
Astrocyte-specific BDNF signaling sufficiently induces memory persistence. **a** Design of Opto-cytTrkB.T1 (Opto-T1), containing a light-sensitive CRY2PHR^E281A^ domain fused to the cytosolic domain of the TrkB.T1 receptor. **b** Representative image showing astrocyte-specific expression of AAV-GFAP-Opto-T1-HA. **c** Quantification of astrocytic specificity of Opto-T1 expression. Each dot indicates one slice. A total of 311 HA^+^ cells were analyzed from 4 slices across 3 mice, and specificity was quantified for each slice and averaged across slices (mean ± SEM). **d** Experimental scheme for unilateral virus injection and non-invasive continuous blue light stimulation (0.5 mW/mm^2^ in the home cage). **e** Representative GFAP and HA immunostaining of contralateral (non-injected) and ipsilateral (Opto-T1-injected) hippocampal CA1. **f** Comparison of GFAP volume without (–light, *n* = 3 mice; Contra, 32 astrocytes; Ipsi, 123 astrocytes) or with (+light, *n* = 3 mice; Contra, 49 astrocytes; Ipsi, 88 astrocytes) light stimulation (contra(-light) vs. ipsi(+light), *P* = 0.0144; contra(+light) vs. ipsi(+light), *P* < 0.0001; ipsi(-light) vs. ipsi(+light), *P* = 0.0004). **g **fEPSP slope changes by three theta-burst stimulations (3×TBS) with or without BDNF (50 ng/ml, 10 min) or Opto-T1 activation (50 µW/mm^2^, continuous for 10 min). naïve, *n* = 9 slices from 5 mice; naïve+BDNF, *n* = 9 from 5 mice; Opto-T1, *n* = 12 slices from 5 mice; Opto-T1+light, *n* = 12 slices from 5 mice. **h** Summary graph of late-phase LTP (L-LTP) (naïve(-BDNF) vs. naïve(+BDNF), *P* = 0.0083; Opto-T1(-light) vs. Opto-T1(+light), *P* = 0.0145). **i** Schematic diagram of contextual fear conditioning with continuous light stimulation (0.5 mW/mm^2^ in the home cage). **j**–**m** Freezing behavior measured across different phases. **j** Acquisition. Acquisition data represent mean freezing across mice during BL and post-shock period in each group. **k** Recent and remote memory test (control, *P* = 0.0002; Opto-T1, *P* = 0.0415). **l** Memory persistence in mice injected with control virus or Opto-T1 virus, with blue light stimulation 12 h after memory acquisition (control vs. Opto-T1, *P* = 0.019). **m** Novel context test. Control, *n* = 13 mice; Opto-T1, *n* = 13 mice. Only male mice were used for behavioral experiments. Data are mean ± SEM. *P < 0.05, **P < 0.01, ***P < 0.001, ****P < 0.0001. ns, not significant. Dunn’s multiple comparisons test (**f**), unpaired *t* test (**h**, **l**), two-way repeated measures ANOVA (**j**), Wilcoxon test (**k**, control), paired *t* test (k, Opto-T1), and Mann–Whitney test (m). All statistical tests were two-sided. Detailed statistics are provided in Supplementary Data 1. Panels **a**, **b**, **d**, **g**, **i** were created in BioRender. Koh, W. (2026) https://BioRender.com/4hltk5u. Source data are provided as a Source Data file.

We first engineered an actuator based on the previously reported Opto-cytTrkB^49^, which contains the CRY2PHR^E281A^ domain fused to the cytosolic domain of full-length TrkB, enabling blue-light activation while preventing activation by endogenous ligands. Because astrocytes express the truncated TrkB.T1 receptor^40–42^, we replaced the cytoplasmic module with the TrkB.T1 intracellular domain (Fig. 8a), creating a tool applicable to astrocytes. This construct, termed Opto-T1 (Opto-cytTrkB.T1), was expressed under the astrocyte-specific GfaABC1D promoter (AAV-GFAP-Opto-T1-HA) to ensure selective activation in astrocytes (Fig. 8b). HA immunostaining revealed bushy astrocyte morphology predominantly co-labeled with GFAP (Fig. 8b, c), confirming astrocyte-specific expression.

Next, we unilaterally injected AAV-GFAP-Opto-T1-HA into hippocampal CA1 to allow within-animal comparison. For non-invasive activation of Opto-T1, we placed a customized LED lid over the home cage, which delivered continuous blue light stimulation (~0.5 mW/mm^2^, 10 min) to freely moving mice (Supplementary Fig. 8a). This stimulation induced robust astrocytic morphogenesis on the injected side compared to the contralateral side (Fig. 8d–f), recapitulating BDNF-dependent changes in vivo (Supplementary Fig. 7c–e). Additional measurements indicated that, despite strong attenuation through the overlying scalp-skull-cortex tissues, transcranial blue light remains sufficient to activate Opto-T1 in hippocampal astrocytes in vivo (Supplementary Fig. 8b–h).

We next asked whether activation of astrocytic TrkB.T1 signaling by Opto-T1 could reproduce the well-established contribution of BDNF to maintaining L-LTP^50^. Unlike 12×TBS (Figs. 4a), 3×TBS in naïve hippocampal slices induced only E-LTP (Fig. 8g, h), consistent with the previous report^50^. Application of BDNF (50 ng/ml, 10 min) 1 h after 3×TBS allowed the maintenance of LTP for at least 3 h. To test whether astrocytic TrkB.T1 activation could reproduce this effect, we performed LTP recordings in mice injected in hippocampal CA1 with AAV-GFAP-Opto-T1-HA (co-injected with AAV-EF1α-mCherry to verify the injection site). In hippocampal slices from Opto-T1-expressing mice, continuous blue light stimulation (50 μW/mm^2^, 10 min, 1 h post-3×TBS) likewise maintained LTP for at least 3 h (Fig. 8g, h), indicating that astrocytic TrkB.T1 activation is sufficient to sustain LTP maintenance.

Finally, we examined whether astrocytic TrkB.T1 activation by Opto-T1 could enhance memory persistence. Mice were injected in hippocampal CA1 with either AAV-GFAP-Opto-T1 or a light-insensitive mutant (Opto-T1^D387A^) and subsequently subjected to mild contextual fear conditioning. Non-invasive continuous blue light stimulation (0.5 mW/mm^2^, 10 min) 12 h after training significantly enhanced remote memory and persistence without altering acquisition or recent recall (Fig. 8i–l). Freezing levels in a novel context did not differ between groups (Fig. 8m), indicating that the enhancement was specific to the conditioned context and did not reflect generalized fear.

These findings demonstrate that astrocytic TrkB.T1 activation alone is sufficient to mimic BDNF-dependent astrocyte morphogenesis, LTP maintenance, and memory persistence when engaged 12 h after learning. Together with our earlier results, this complements the conclusion that astrocytic Ank2 is necessary, whereas BDNF-TrkB.T1 signaling is sufficient, for these processes.

## Discussion

In this study, we demonstrate that astrocytic Ank2 plays a crucial role in astrocyte morphogenesis, remote memory formation, and memory persistence. While Ank2 expression in astrocytes has been reported, its function remained unexplored, as most studies focused on neuronal Ank2^20,21^. Our findings reveal that astrocytic Ank2 is essential for maintaining astrocyte-neuron interactions at engram synapses, particularly in remote memory stabilization (Supplementary Fig. 9).

Astrocytic leaflets, where Ank2 is found to be expressed^24–27^, dynamically retract and extend during learning^5^. Their initial withdrawal after LTP^7^ or learning^6^ has been proposed to facilitate synaptic modifications. However, whether this withdrawal occurs specifically at engram synapses remains unclear, as it may also occur at inactive synapses to promote heterosynaptic plasticity^51^. Eventually, astrocytic leaflets extend and increase synapse coverage, particularly around enlarged spines, thereby enhancing spine stability^8^. Supporting this, recent studies have shown that learning-associated astrocytes exhibit increased eGRASP signals following learning^9^. More specifically, astrocyte-eGRASP imaging revealed memory-state-dependent astrocyte contacts, with increased contact after learning and reduced contact following memory extinction^10^. Interestingly, we found that the number of astrocyte contacts with engram neurons was already decreased in Ank2^GFAP^ cKO mice, despite no difference in recent memory recall. This finding extends previous reports on astrocyte contacts and recent memory recall^9,10^, underscoring the importance of astrocyte contacts in memory persistence. This raises the possibility that such memory-supporting astrocytic processes may constitute part of an “astrocytic engram,” a structural and functional trace within astrocytes^9,52,53^ that parallels neuronal engrams in sustaining remote memory. In this context, our findings suggest that Ank2 is a crucial element of the astrocytic engram, enabling structural changes in astrocytes that support the long-term stability of memory traces. Further validating astrocyte-eGRASP at remote time points will be important to determine how astrocytic contacts on engram neurons are maintained during long-term memory consolidation.

Our findings strengthen the evidence for the pivotal role of astrocytes in remote memory processing. Recent studies have shown that chemogenetic Gq activation in hippocampal astrocytes enhances recent memory, whereas Gi activation suppresses remote memory^54,55^. However, most astrocytic manipulations to date have focused on the timing of memory acquisition^56^. Given that expression of BDNF 12 h after learning is a critical factor for memory persistence^39,48^, we investigated the involvement of astrocytic Ank2 in BDNF-mediated memory persistence at this time point. In this study, we showed that astrocytic Ank2 is essential for BDNF-dependent enhancement of memory persistence 12 h after mild learning. Furthermore, optogenetic stimulation of astrocytic TrkB.T1 signaling at the same time point after mild learning selectively enhanced remote memory without affecting recent memory. This finding highlights a temporally sensitive window during which astrocytic signaling influences memory persistence, providing a novel target for astrocyte-specific memory modulation. It should be noted that strong non-invasive blue light itself may influence baseline freezing levels^57^ or retinal damage^58^, and therefore comparisons should be made between groups exposed to identical light conditions. Further investigations are needed to determine whether different methods of astrocytic activation during this period elicit distinct effects on memory process. Additionally, it would be valuable to explore whether astrocytic Gi activation during learning^54,55^ modulates astrocytic responses to BDNF over time. Notably, although BDNF infusion enhanced memory persistence in control animals, this effect was reversed in astrocytic Ank2 cKO mice. This may reflect that, while BDNF broadly promotes synaptic plasticity, astrocytic Ank2-dependent morphogenesis contributes to circuit specificity by stabilizing astrocyte-synapse interactions. Loss of Ank2 may weaken this selective stabilization, allowing BDNF-driven remodeling to become less specific and thereby destabilize circuits supporting memory persistence. TrkB.T1 plays an essential role in astrocytic morphogenesis. Deletion of TrkB.T1 impairs astrocytic morphogenesis^40,41^, and here we demonstrate that astrocyte-specific activation of TrkB.T1 is sufficient not only to drive morphogenesis but also to enhance memory persistence. However, the downstream signaling mechanisms remain to be fully elucidated. Although the present work does not directly define these pathways, the Opto-T1 approach provides a framework for future studies to dissect the molecular events linking astrocytic TrkB.T1 activation to structural plasticity. Especially, as Opto-T1 enables spatiotemporal activation, which is not feasible with conventional BDNF treatment, it offers a unique advantage for establishing more precise mechanistic insights into astrocytic morphogenesis and structural plasticity.

Interestingly, ezrin expression was reduced in Ank2 cKO astrocytes, which is unexpected because previous studies reported an inverse relationship between ezrin and GFAP in certain pathological conditions^59^. This correlation is not universal, as ezrin deletion in astrocytes has also been reported to show a trend toward decreased GFAP expression^6^. Ezrin has also been identified as a binding partner of Ank2 by affinity purification mass spectrometry^60^, suggesting that Ank2 may influence ezrin localization or stability, although this remains untested in astrocytes. As a structural scaffold, Ank2 shapes astrocytic morphology and interacts with many other proteins, making it difficult to separate direct from indirect effects in this study.

It should be acknowledged that the present study focused primarily on the BDNF-IP3R2 pathway, yet other mechanisms could also contribute to the observed phenotypes. In this context, chemogenetic astrocytic activation (e.g., DREADDs) may offer a complementary approach to test whether memory persistence can be rescued in Ank2^GFAP^ cKO mice. Exploring these alternative possibilities will be necessary to fully understand the roles of Ank2 in astrocytic Ca^2+^ signaling and neuron-astrocyte interactions. For example, the reduced Ca^2+^ activity in Ank2 cKO astrocytes may reflect not only impaired IP3R2-dependent signaling, as seen in IP3R2 knockout mice, but also consequences of astrocytic atrophy and reduced neuron-astrocyte communication. Downstream of BDNF-IP3R signaling, store-operated Ca^2+^ channels have been proposed as an additional source of Ca^2+,^ which could further amplify the effects of BDNF. Interestingly, in cardiomyocytes, Ank2 has been reported to interact with Na^+^-K^+^ ATPase and Na^+^-Ca^2+^ exchanger^61^, both of which are important Ca^2+^ sources in astrocytes^62^. The potential role of Ank2-interacting proteins in the ER and plasma membrane (PM) in assembling complexes^43^ that may facilitate ER-PM junction formation and modulate Ca^2+^ signaling in astrocytes warrants further study.

Finally, while ex vivo recordings revealed impaired LTP maintenance in Ank2 cKO mice, this finding should be interpreted with caution. Although LTP has long been regarded as a cellular correlate of memory storage, actual learning and memory formation involve multiple levels of consolidation, from synaptic to systems-level processes. Therefore, impairment of L-LTP over 3 h cannot be directly equated to a 3-h limit in memory retention. Nevertheless, the data indicate a reduced ability to maintain synaptic potentiation, and it is possible that diminished astrocytic metabolic support in Ank2 cKO mice limits the capacity to sustain potentiated synapses. Interestingly, we did not detect significant changes in the ending radius of GFAP-positive branches, which may suggest that a minimal level of metabolic support and astrocytic syncytium is still preserved. Given that astrocytic contacts on engram neurons may serve as hubs for such metabolic support to maintain enlarged spines, future work exploring this possibility could provide important mechanistic insight.

While hippocampal CA1-restricted deletion of astrocytic Ank2 was sufficient to impair memory persistence, the behavioral deficit was more modest than that observed following astrocyte-specific Ank2 deletion using GFAP-CreER^T2^. This difference suggests that astrocytic Ank2 in additional brain regions may also contribute to memory persistence, particularly in cortical areas such as the medial prefrontal cortex and anterior cingulate cortex, which are known to support remote memory storage. Beyond its role in memory persistence, Ank2 may have broader functions in astrocyte biology, including the regulation of astrocyte maturation. While GFAP-CreER^T2^ was selected in this study to reduce peripheral recombination, Aldh1l1-CreER^T2^ exhibits pan-astrocytic expression and targets a broader range of astrocyte subtypes, which could reveal additional roles of Ank2 in astrocyte populations not efficiently targeted by GFAP-based approaches. Notably, astrocyte-specific ARL13B deletion using Aldh1l1-CreER^T2^, which disrupts primary cilia, leads to reduced Ank2 expression and a decrease in astrocytic surface area^63^. Given the link between astrocytic structural integrity and neurodevelopmental disorders such as ASD, further investigation is warranted to determine how Ank2 contributes to astrocyte maturation and whether it influences brain development. Whether Ank2 primarily regulates astrocytic morphology and signaling or also contributes to broader neurodevelopmental processes remains an open question, with potential implications for ASD and intellectual disability.

## Methods

### Animals

All experimental procedures were approved by the Institutional Animal Care and Use Committee (IACUC) of the Institute for Basic Science (IBS) and conducted in accordance with the Guide for the Care and Use of Laboratory Animals. Mice were housed under a 12:12-h light-dark cycle (lights on at 8:00 AM) with ad libitum access to food and water. Both male and female C57BL/6 J wild-type mice (8-25 weeks old) were utilized in the study, with only males being used for behavioral tests. All experiments used age-matched littermate controls, and all mice were maintained on a C57BL/6 J background. Astrocyte-specific Ank2 conditional knockout (Ank2^GFAP^ cKO) mice were generated by crossing *Ank2*^*fl/fl*^ mice^21^ with GFAP-CreER^T2^ mice^29^, yielding *Ank2*^*fl/fl*^; GFAP-CreER^T2^ and *Ank2*^*fl/fl*^ littermates as controls. To induce Ank2 deletion in adult astrocytes, tamoxifen was administered intraperitoneally (100 mg/kg per day, dissolved in sunflower oil/ethanol 9:1) for five consecutive days. Behavioral and physiological experiments were performed at least two weeks after the final injection.

### Behavioral tests

The mice were handled for one or two days prior to the behavioral experiments to minimize stress. On the day of the experiment, the mice were acclimated to the testing room for one hour before starting the behavioral tests. Between each experiment, the apparatus was thoroughly cleaned with 70% ethanol to eliminate any residual olfactory cues.

#### Open field test

Mice were placed individually in the center of an open field apparatus (40 × 40 cm arena, with 40 cm high walls) and allowed to explore freely for 10 min. The center zone was defined as a 20 × 20 cm area in the middle of the arena. The locomotor activity, including the distance traveled and time spent in each area (center and periphery), was recorded and analyzed using an automated video tracking system (EthoVision). After each trial, fecal boli were manually counted once the mouse was returned to its home cage.

#### Contextual fear conditioning

For both the recent and remote memory tests, a hexahedral conditioning chamber (18 cm wide × 18 cm long × 30 cm height; H10-11M-TC; Coulbourn Instruments) was used. The chamber consisted of a metal grid floor, aluminum sidewalls, and a clear plexiglass front door and back wall. In the conditioning protocol, after an acclimation period of 208 s, mice were exposed to a strong unconditioned stimulus consisting of three foot-shocks (0.75 mA, 2 s each) delivered at 30-s intervals. Following the final foot-shock, the mice remained in the conditioning chamber for an additional 30 s before being returned to their home cages. For the recent memory test, conducted 24 h post-conditioning, the mice were placed in the conditioned context chamber for 5 min, during which freezing behavior was measured. For the remote memory test, conducted 14 days post-conditioning, the same procedure was repeated. Freezing behavior was analyzed using Freezeframe software, and memory persistence was assessed by calculating the ratio of freezing time on day 14 to day 1.

For the extinction test, the protocol consisted of 4 days. On the first day, after an acclimation period of 208 s, mice were exposed to three foot-shocks (0.75 mA, 2 s each) delivered at 30-s intervals. On the following day, mice were placed back into the conditioned context chamber for 30 min. Freezing behavior was measured every 5 min, resulting in 6 blocks. The same procedure was repeated on the third day. On the final day, freezing behavior was measured for 5 min and compared to the first block on the second day to calculate memory extinction.

For remote memory enhancement with BDNF infusion or Opto-T1, we used a mild conditioning protocol (single foot-shock, 0.75 mA, 2 s) following a 208-s acclimation period, as previously described^48^. Mice remained in the chamber for 30 s after the shock before returning to their home cages. Twelve hours after acquisition, either BDNF (100 ng in 0.4 µl per side, delivered bilaterally at 0.2 µl/min) or optogenetic stimulation was applied. Detailed surgical procedures for BDNF infusion are described in Cannula implantation and infusion of BDNF in the hippocampus. Non-invasive blue light stimulation (~0.5 mW/mm^2^ for 10 min) was delivered using a custom blue-light LED cage lid (Scitech Korea), with minor modifications from a previously described design^49^. Recent and remote memory were assessed 24 h and 14 days after conditioning, respectively, by measuring freezing for 5 min in the conditioned context. To test context specificity, mice were exposed to a novel cylindrical arena (20 cm diameter, 25 cm height) with bedding on the floor for 5 min one day after the remote memory test.

#### Three-chamber test

The three-chamber test was conducted in three 10-min sessions within a three-chambered open arena (40 cm length × 20 cm width × 20 cm height for each chamber). In the habituation session, empty metal cups were placed in both side chambers, and the mouse was placed in the central chamber. After opening the partition, the mouse freely explored the apparatus for 10 min. At the end, the mouse was gently guided back to the central chamber, and the partition was closed. In the sociability session, a stranger mouse (stranger mouse 1, S1) was placed in one of the metal cups, and the partition was opened again, allowing the mouse to freely explore for 10 min. Afterward, the partition was closed. In the social novelty session, a new stranger mouse (stranger mouse 2, S2) was placed in the empty metal cup, and the mouse was allowed to explore freely for 10 min with the familiar and new mice. The duration of exploration/sniffing of each cup was manually analyzed by a researcher blinded to the experimental conditions.

#### Marble burying test

In the marble burying test, 20 glass marbles (1.5 cm in diameter) were arranged in a 4 by 5 grid on top of 3 cm of clean bedding. Mice were placed in the center of the cage and allowed to freely explore for 30 min. After the session, the number of marbles buried (defined as having at least two-thirds of the marble covered by bedding) was counted in a blind manner.

#### Object location memory test

To assess object location memory, mice were first habituated to an open-field arena (40 × 40 cm with 40 cm high walls) for 10 min. On the following day, two identical objects were placed in the first and second quadrants of the arena, and mice were allowed to freely explore for 10 min. Two days later, during the test phase, one of the objects was relocated to the opposite, previously empty quadrant. Mice were given 5 min to explore the arena, and their exploration behavior was recorded with a video camera and analyzed offline by an experimenter blinded to the genotypes.

#### Circadian activity and metabolic rhythm measurement

Circadian activity and metabolic rhythms were assessed using the Phenomaster metabolic cage system (TSE, Bad Homburg, Germany). Mice were provided with free access to water and standard diet pellets. Locomotion and energy expenditure were monitored for two days following a three-day acclimation period in the recording cages. According to the manufacturer’s instructions, the following parameters were measured and calculated: activity (beam break counts), food consumption (g), water consumption (ml), O_2_ consumption (VO_2_, ml/kg/hr), CO_2_ production (VCO_2_, ml/kg/hr), and the respiratory exchange ratio (RER).

### Construction of viral vectors expressing short hairpin RNA (shRNA)

To generate viral vectors expressing shRNA, the targeting sequences were cloned into miR30-based vectors.

Specifically, AAV-GfaABC1D-mCherry-shLuci vector was used for cloning^64^, with a renilla luciferase-targeting sequence (shLuci; the sense sequence 5’-CAGGAATTATAATGCTTATCTA-3’) serving as a control in the present study. The sense sequence for ITPR2 was 5’-GCATCTCAATCTGTTCCTAAC-3’^64^. Based on this targeting sequence, the following viral vector was generated: AAV-GfaABC1D-mCherry-shITPR2.

### Stereotaxic surgeries

Viruses were produced from Institute for Basic Science (IBS) virus facility (Daejeon, Republic of Korea). Mice were anesthetized with isoflurane and mounted into stereotaxic frames (David Kopf Instruments, Tujunga, CA, USA). Viruses were injected using syringe pump (KD Scientific, Holliston, MA, USA), bilaterally unless otherwise specified, into CA1 of hippocampus with the following coordinates (from bregma): anterior-posterior, –1.9 mm; medial-lateral, ±1.45 mm, dorsal-ventral, –1.5 mm.

### Electrophysiology, Ca^2+^ imaging, immunohistochemistry from acute brain slices

#### Preparation of acute brain slice for electrophysiological experiments, Ca^2+^ imaging, and BDNF treatment

Mice were anesthetized using isoflurane and then decapitated. The brain was quickly isolated and sectioned into 300-μm-thick coronal slices using a Compresstome (VF-510-0Z, Precisionary) or a vibrating microtome (Linear Slicer Pro7N, DSK) in ice-cold, oxygenated (95% O_2_/5% CO_2_) sucrose-based dissection buffer. The dissection buffer contained the following components (in mM): 5 KCl, 1.23 NaH_2_PO_4_, 26 NaHCO_3_, 10 glucose, 0.5 CaCl_2_, 10 MgSO_4_, and 212.5 sucrose. Brain slices were allowed to recover for at least 1 h in oxygenated artificial cerebrospinal fluid (aCSF) containing (in mM): 124 NaCl, 5 KCl, 1.25 NaH_2_PO_4_, 2.5 CaCl_2_, 1.5 MgCl_2_, 26 NaHCO_3_, and 10 glucose at room temperature.

#### Whole-cell patch recording for astrocyte and CA1 pyramidal neurons in the hippocampus

Astrocyte patch pipettes were filled with an intracellular solution composed of (in mM): 60 KCl, 80 K-gluconate, 5 EGTA, 10 HEPES, 4 Mg-ATP, and 0.3 Na-GTP, pH 7.35, with an osmolarity of 280 mOsm/kg. To visualize astrocyte morphology and astrocyte gap junction coupling, 300 μM Texas Red Dextran 3 kDa (Invitrogen) and 0.2% biocytin (B4261, sigma) were added to the intracellular solution. Astrocytes were identified by their characteristic morphology, passive conductance, and negative resting membrane potential. For assessing passive conductance, a voltage step protocol was applied ranging from -180 to 20 mV in 20 mV intervals. The rectification index was calculated as the difference in current between +20 mV and -80 mV, divided by the difference in current between –80 mV and –180 mV. After a stable dialysis period of 20 min, patch pipette was gently detached from the cells and the slice was fixed in 4% paraformaldehyde in PBS overnight at 4 °C for further immunohistochemistry.

For the excitability test in CA1 pyramidal neurons, patch pipettes were filled with an intracellular solution composed of (in mM): 126 K-gluconate, 10 HEPES, 0.5 MgCl_2_, 10 BAPTA, 4 Mg-ATP, and 0.3 Na-GTP, pH 7.2, with an osmolarity of 281 mOsm/kg. For the spontaneous excitatory postsynaptic currents (EPSCs) and inhibitory postsynaptic currents (IPSCs) measurements, patch pipettes were filled with an intracellular solution composed of (in mM): 130 CsMeSO_4_, 10 HEPES, 10 EGTA, 9 TEA-Cl, 5 QX-314, 4 Mg-ATP, and 0.3 Na-GTP, pH 7.35, with an osmolarity of 283 mOsm/kg. Spontaneous IPSCs and EPSCs were measured at 0 mV and –70 mV, respectively. The amplitude and frequency of spontaneous IPSCs and EPSCs were detected and measured using MiniAnalysis 6.0.7 (Synaptosoft). Electrical signals were digitized and sampled at 50 μs intervals with Digidata 1440 A data acquisition system and the Multiclamp 700B Amplifier (Molecular Devices) using the pClamp10.7 software. Data were filtered at 2 kHz.

#### Confocal astrocytic Ca^2+^ imaging and analysis

To access astrocytic Ca^2+^ dynamics, AAV-CAG-FLEX-NES-jRCaMP1a and AAV-GFAP-Cre-4x6T were co-injected into *Ank2*^*+/+*^ or *Ank2*^*fl/fl*^ mice. This approach enabled Cre-dependent expression of jRCaMP1a in astrocytes, with or without the Cre-mediated deletion of Ank2. Acute brain slices were then prepared as described above. Fluorescence imaging was conducted using a laser scanning confocal microscope (Nikon A1R MP).

For the analysis, we followed the approaches from previous studies^44,65^. First, the detection of Regions of Interest (ROIs) displaying Ca^2+^ elevations was performed using ImageJ in a semi-automated manner with the GECIquant plugin^44^. ROIs were initially identified based on the soma, with an area threshold set at 30 μm^2^ to infinity for somatic ROIs and 0.5–4 μm^2^ for microdomains. Fluorescence intensity values for each ROI were extracted using ImageJ and then converted into ∆F/F values to quantify relative fluorescence changes. To normalize fluorescence comparisons across different astrocyte, the Ca^2+^ signal was expressed as ∆F/F_0_ = (F_t_ - F_0_)/F_0_, where F_0_ was defined as the 15th percentile of the entire fluorescence trace per ROI, serving as a global baseline. The baseline trace for each ROI was determined by selecting points in the ∆F/F_0_ trace with absolute values smaller than twice the standard deviation of the overall signal.

#### BDNF treatment

After preparing brain slices, they were allowed to recover for 1 h in aCSF before conducting the BDNF treatment experiment. For the BDNF treatment, recombinant human BDNF (R&D Systems) was prepared as a stock solution (250 µg/ml). For the control condition, denatured BDNF was prepared by boiling BDNF at 100 °C for 10 min. BDNF and boiled BDNF were then diluted in aCSF to a final concentration of 50 ng/ml. Brain slices were incubated in these solutions for 30 min, after which they were transferred to normal aCSF for an additional 30 min to allow for further morphological responses. Subsequently, the slices were post-fixed overnight in 4% paraformaldehyde (PFA) in 0.1 M PBS, followed by immunohistochemistry the next day.

#### Field excitatory postsynaptic potentials (fEPSP) recording

To assess basal synaptic transmission, paired-pulse ratio (PPR), and long-term potentiation (LTP), preparation of hippocampal slice and fEPSP experiments were conducted as previously described^51^. Briefly, mice were anesthetized with isoflurane and decapitated. The brain was then quickly isolated and sectioned into 400-μm-thick transverse hippocampal slices using a vibratome (VT1200S, Leica) in ice-cold, oxygenated (95% O_2_/5% CO_2_) sucrose-based dissection buffer. The dissection buffer contained the following components (in mM): 5 KCl, 1.23 NaH_2_PO_4_, 26 NaHCO_3_, 10 glucose, 0.5 CaCl_2_, 10 MgSO_4_, and 212.5 sucrose. The hippocampal slices were allowed to recover for at least 1 h in oxygenated aCSF containing (in mM): 124 NaCl, 5 KCl, 1.25 NaH_2_PO_4_, 2.5 CaCl_2_, 1.5 MgCl_2_, 26 NaHCO_3_, and 10 glucose at 28  ±  1 °C. The fEPSPs were evoked in the Schaffer collateral pathway by applying an electrical stimulus, generated by a stimulus isolator (A365R, WPI), using a concentric bipolar electrode (CBBPE75, FHC). For recording fEPSPs, an aCSF-filled recording pipette, made from borosilicate glass capillary (1–3 MΩ, Harvard Apparatus), was placed in the stratum radiatum of the hippocampal CA1 region. The fEPSP slope was measured and analyzed using WinLTP v2.01 software (WinLTP Ltd.). Basal synaptic transmission was evaluated by gradually increasing the stimulus intensity by 50 pA increments from 0 to 300 pA. In subsequent experiments, the stimulus intensity was adjusted to 40–45% of the maximum response. For the PPR experiment, two pulses were delivered at intervals of 10, 25, 50, 100, 250, 500, and 1000 ms, and the ratio was calculated by dividing the fEPSP slope of the second response by that of the first response. For the LTP experiment, the fEPSP slope was monitored at 0.067 Hz (one pulse every 15 s). After stable fEPSP responses were recorded for at least 20 min, LTP was induced using a theta-burst stimulation (TBS) protocol consisting of either 12 or 3 bursts, each composed of 4 pulses at 100 Hz (referred to as 12×TBS or 3×TBS, respectively). Potentiation was quantified by averaging the fEPSP slopes, normalized to baseline, over the last 10 min of recording at each time point: 60, 120, and 180 min for 12×TBS, and only 180 min for 3×TBS. For the Opto-T1 experiments, AAV-GFAP-Opto-T1-HA and AAV- EF1α-mCherry were co-injected in hippocampal CA1. To activate Opto-T1 expressed in astrocytes, blue light (473 nm; 50 μW/mm^2^, MBL-III-473 nm, CNI Laser) was delivered for 10 min starting 1 h after 3×TBS stimulation.

### Cannula implantation and infusion of BDNF in the hippocampus

For cannula implantation, mice were anesthetized with isoflurane and secured in stereotaxic frames (David Kopf Instruments, Tujunga, CA, USA). After making an incision in the scalp, a hole was drilled into the skull above the CA1 region at coordinates (anterior/posterior: −2 mm; medial/lateral: ±1.5 mm; from bregma), with coordinates determined using the Allen Mouse Brain Atlas. Bilateral guide cannula (8IC235G30XXC-2MM, Protech international, Inc) containing dummy cannula (8IC235DCSPCC-2.0-2MM-0.5MMPROJ, Protech international, Inc) was implanted at a dorsoventral coordinate of –1.5 mm from bregma and secured to the skull using Charisma Classic (Kulzer). Mice were then allowed to recover for at least five days.

On the day of BDNF infusion, the dummy cannula was carefully removed, and an internal cannula (8IC235ISPCXC-3.0-2MM-0.5MMPROJ, Protech International, Inc.) was inserted into the guide cannula. Recombinant human BDNF (R&D Systems) was dissolved in ACSF at a concentration of 250 ng/µl. Infusion was performed using a microinfusion pump connected via polyethylene tubing (PE20) to a 25-μl Hamilton syringe filled with mineral oil (Sigma). BDNF was delivered bilaterally at a rate of 0.2 μl/min for 2 min, with a total volume of 0.4 μl (100 ng) per side. The internal cannula remained in place for an additional minute to ensure the solution was properly delivered into the hippocampus with minimal leakage. To validate BDNF-dependent astrocyte morphogenesis in vivo, mice were sacrificed 1 h after BDNF infusion.

### Construction of pAAV-GfaABC1D-Opto-T1 and pAAV-GfaABC1D-IP3R2 ABD-4x6T

For the construction of the main viral vectors, we used pAAV-CamK2a(0.4)-Opto-cytTrkB(PHR^E281A^)-HA (Addgene, #180589)^49^ and pAAV-GfaABC1D-EGFP (Addgene, #50473). First, the PCR-amplified product (i.e., Opto-cytTrkB(PHR^E281A^)-HA) from pAAV-CamK2a(0.4)-Opto-cytTrkB(PHR^E281A^)-HA was cloned into pAAV-GfaABC1D-EGFP at the XbaI and EcoRI sites using the EZ-Fusion^TM^ HT Cloning kit (Enzynomics), resulting in the construction of pAAV-GfaABC1D-opto-cytTrkB(PHR^E281A^)-HA. To replace cytosolic TrkB with cytosolic TrkB.T1, pAAV-GfaABC1D-opto-cytTrkB(PHR^E281A^)-HA was first cut with XbaI and BamHI to serve as the backbone for pAAV-GfaABC1D-opto-cytTrkB.T1(PHR^E281A^)-HA. We then amplified the N-terminal sequence, including the myristoylation sequence (Lyn signal peptide) and up to the shared sequence between TrkB and TrkB.T1, from pAAV-GfaABC1D-opto-cytTrkB(PHR^E281A^)-HA by PCR. Finally, we utilized oligomers containing the rat cytosolic TrkB.T1 sequence, including the RhoGDI1 (Rho GDP dissociation inhibitor) binding site, and inserted them into the digested backbone, resulting in the construction of pAAV-GfaABC1D-opto-cytTrkB.T1(PHR^E281A^)-HA. Light-insensitive pAAV-GfaABC1D-opto-cytTrkB.T1(PHR^E281A, D387A^)-HA mutant was generated by site-directed mutagenesis.

To construct pAAV-GfaABC1D-IP3R2 ABD-4x6T, we used pAAV-GfaABC1D-eGFP-4x6T as the backbone plasmid. The backbone was digested with BsrGI and BamHI, and annealed oligomers containing P2A and the IP3R2 ABD sequence were inserted into the digested sites via in-fusion cloning.

### Unilateral transcranial blue light stimulation for Opto-T1 activation

First, Opto-T1 was systemically expressed via retro-orbital injection of AAV-PHP.eB-GFAP-Opto-T1-HA, resulting in broad astrocytic expression across the brain. Three weeks later, the mice were anesthetized with isoflurane. To accurately localize the stimulation site, the scalp was briefly incised to expose the skull surface, stereotaxic coordinates were identified (AP -1.9 mm, ML + 1.45 mm from bregma), and the scalp was sutured back prior to stimulation. Continuous blue light (473 nm, 0.5 mW/mm^2^, 10 min; MBL-III-473, CNI Laser) was then applied transcranially to the right hemisphere. The mice were sacrificed 1 h after stimulation by transcardial perfusion. Following fixation, brains were extracted, and a notch was made in the left hemisphere to mark the unstimulated side for subsequent sectioning. Coronal sections (40 µm) were processed for subsequent immunohistochemistry.

### Laser attenuation through scalp, skull and cortex (SSC) tissues

Laser power was measured using a PM100D optical power meter with an S121C photodiode sensor (Thorlabs, Newton, NJ, USA), under blue light illumination (0.5 mW/mm^2^). Cadaver-derived scalp, skull and cortex tissues (SSC) were placed over the detector during illumination to measure attenuation through overlying tissues, with the cortex approximately 1 mm thick.

### Western blotting

To examine differences in protein expression between ctrl and Ank2^GFAP^ cKO mice, hippocampal tissues were dissected and lysed with RIPA buffer (pH 7.4, 50 mM Tris-HCl, 150 mM NaCl, 5 mM EDTA, 1 mM PMSF, and 1% NP-40) containing protease inhibitor cocktail (P3100, GenDEPOT) and phosphatase inhibitor cocktail (P3200, GenDEPOT). The obtained protein lysates were separated by SDS-PAGE using 10% gels for Ank2 and Ezrin proteins and transferred onto PVDF membranes. The blots were incubated with a rabbit anti-Ank2 antibody (75–145, NeuroMab) or a rabbit anti-Ezrin antibody (3145S, Cell Signaling), followed by appropriate horseradish peroxidase (HRP)-conjugated secondary antibodies (Jackson ImmunoResearch). Protein signals were detected using enhanced chemiluminescence (ECL, GE Healthcare), and band intensity was acquired using ImageQuant LAS 4000 (GE Healthcare). To detect β-actin, membranes were incubated in 1 N NaOH for 10 min for stripping, followed by re-incubation with a rabbit anti-β-actin antibody (ab133626, Abcam) and the corresponding HRP-conjugated secondary antibody (Jackson ImmunoResearch). Western blot bands were quantified using ImageJ software (NIH).

### Co-Immunoprecipitation

The whole brain of the adult C57BL/6 mice was dissected and homogenized with a homogenizer (PowerMasher II, Nippi) in ice-cold Pierce™ IP Lysis Buffer (87788, Thermo Scientific) with protease inhibitor cocktail (P3100, GenDEPOT) and phosphatase inhibitor cocktail (P3200, GenDEPOT), incubated on ice for 20 min and centrifuged at ~20,000 × *g* for 20 min at 4 °C. The protein supernatant was collected, and input from the supernatant was separated at this step to be used for western blotting. The remaining protein supernatants were subjected to immunoprecipitation with 1 μg of rabbit anti-IP3R1 (07-1213, Merck Millipore), rabbit anti-IP3R2 (ab3000, Merck Millipore), rabbit anti-IP3R3 (ab9076, Merck Millipore), and normal rabbit anti-IgG antibodies (2729, Cell Signaling Technology), respectively, overnight at 4 °C, followed by 1 h incubation with 30 μl of precleared 50% slurry of protein A/G agarose beads (WC322042, Thermo Fisher Scientific) at 4 °C. After incubation, the beads were washed 5 times with the IP lysis buffer. After carefully removing the last supernatant, the protein complex with beads were prepared in PAGESTA Reducing 5× SDS-PAGE sample buffer (GeneAll) by boiling at 98 °C for 10 min, and western blotting was then performed with mouse anti-Ank2 antibody (75–145, Neuromab).

### Immunohistochemistry (IHC)

#### IHC for cryostat-dissected brain slices

Mice were deeply anesthetized with isoflurane and transcardially perfused with 0.9% saline followed by ice-cold 4% PFA in 0.1 M PBS. The excised brains were post-fixed overnight at 4 °C and then transferred to 30% sucrose in 0.1 M PBS for 48 h. The brains were sectioned into 40 or 100 μm coronal slices using a cryostat (CM1950, Leica). After washing with PBS, brain sections were translocated into 24-well plates filled with blocking solution (0.3% Triton X-100, 4% donkey serum in 0.1 M PBS) and incubated for 1.5 h.

The slices were then transferred to blocking solution containing primary antibodies at optimized dilutions and incubated overnight at 4 °C on a shaker. Primary antibodies used for immunostaining included: chicken anti-GFAP (AB5541, Merck Millipore), rabbit anti-HA-Tag (3724S, Cell Signaling Technology), guinea pig anti-NeuN (ABN90, Merck Millipore), rabbit anti-Sox9 (AB5535, Merck Millipore), and rabbit anti-IP3R2 (PA1-904, Invitrogen). The next day, slices were washed three times with PBS, and then incubated with the corresponding secondary antibodies in blocking solution for 1.5 h at room temperature. Following this, slices were washed three more times with PBS, and DAPI (62248, Thermo Fisher Scientific) was added in the second wash step to visualize cell nuclei. Finally, sections were mounted using fluorescent mounting medium (S3023, Dako), dried, and imaged using a Zeiss LSM900 confocal microscope.

#### IHC for acute brain slices

Acute brain slices, which had been fixed overnight in 4% PFA in 0.1 M PBS, were washed three times with PBS the following day. Slices were then incubated in blocking solution (0.3% Triton X-100, 10% donkey serum in 0.1 M PBS) for 1.5 h. Next, slices were transferred to a solution containing primary antibodies (prepared in 0.3% Triton X-100, 4% donkey serum in 0.1 M PBS) and incubated for two days at 4 °C. To visualize biocytin, Avidin-Alexa Fluor™ 488 conjugate (A21370, Invitrogen) was utilized. After three washes with PBS, slices were incubated with secondary antibodies (prepared in 0.3% Triton X-100, 4% donkey serum in 0.1 M PBS) for 24 h at 4 °C. Following secondary antibody incubation, slices were washed three times with PBS and mounted using DAPI Fluoromount-G® (SouthernBiotech). Fluorescent images were acquired using a Zeiss LSM900 confocal microscope.

### Astrocyte-eGRASP technique

Astrocyte-eGRASP technique was used as previously described^10^. Briefly, astrocyte peri-eGRASP was expressed in astrocytes by GfaABC1D promoter, and neuronal post-eGRASP^66^ was expressed in engram neurons by doxycycline induced tetO system. All the constructs were expressed using adeno-associated virus serotype1/2. Confocal images were obtained using a Leica Stellaris5 confocal microscope with ×63 objective with distilled water immersion. eGRASP analysis was conducted using IMARIS 10 (Bitplane, Zurich, Switzerland) software. The length of each dendrite was measured using IMARIS filament function. The number of eGRASP was manually counted along with each denoted filament. The examiner was unaware of the group.

### Transmission electron microscopy (TEM)

Mice were deeply anesthetized with isoflurane and perfused transcardially with 0.15 M cacodylate buffer (pH 7.4) containing 2% paraformaldehyde and 2.5% glutaraldehyde. Brains were removed, and 150-μm-thick coronal sections were cut on a vibratome (VT1000 S, Leica). Sections encompassing the hippocampal CA1 region were dissected into small tissue blocks for EM processing. To enhance visualization of the postsynaptic density (PSD) for synapse quantification, tissue blocks were washed in 0.15 M cacodylate buffer, post-fixed in 2% osmium tetroxide (18459, Ted Pella) for 1 h, and then stained en bloc with 1% uranyl acetate overnight at 4 °C. Following alcohol series dehydration and acetone infiltration, samples were embedded in Epon 812 resin (41420, EMS) and polymerized at 60 °C for 48 h. Ultrathin sections (80 nm) were cut using Leica EM UC7 ultramicrotome, collected onto 200-mesh nickel grids, and post-stained with UranyLess (22409, EMS) and lead citrate (22410, EMS). TEM images were acquired at 3,500× magnification using a Tecnai 20 (Thermo Fisher Scientific). The densities of excitatory and inhibitory synapses were quantified using a systematic-random sampling scheme with a 5 μm × 5 μm unbiased counting frame. Random fields within the CA1 region were selected and analyzed using Fiji/ImageJ.

### Image analysis

Confocal microscopic images were obtained and expression were analyzed using the ImageJ 1.54 h (NIH) and IMARIS 9.9.0 (Bitplane, Zurich, Switzerland) software. With ImageJ, fluorescence intensities were calculated using the mean intensity value of each fluorescence pixels in the marker-positive area. The marker-positive area was defined by thresholding and is converted into a binary mask. The mean intensity of immunostained pixels in the binary mask was calculated. Confocal images of brain sections immunostained with GFAP antibody were used for volume measurement and Sholl analysis with IMARIS software. For measuring GFAP-positive volume of astrocyte, raw image files were used for further analysis using IMARIS software. First, manual surface reconstruction was used to obtain the stratum radiatum region of the hippocampus, and GFAP signals were masked with the reconstructed surface of the stratum radiatum. Then, the astrocyte surface was reconstructed with a newly created GFAP channel. The Filament plugin applied in IMARIS software constructs astrocytic branches. For Sholl analysis, the number of intercepts of GFAP-positive processes at serially concentric circles with 5 µm intervals was counted. For measuring astrocytic volume, images from section of mouse injected with AAV-PHP.eB-GFAP-Lck-GFP virus were used. GFP signals were reconstructed to 3D surface, and volume and area of reconstructed GFP signals were analyzed.

### STRING analysis

STRING database (version 12.0, https://string-db.org) was used to construct a protein-protein interaction (PPI) network for *Mus musculus* Ank2. The resulting network consisted of 11 nodes and 30 edges, with an expected edge count of 11, an average node degree of 5.45, an average local clustering coefficient of 0.833, and a PPI enrichment p value of 3.12×10^-6^. Gene Ontology enrichment analysis for molecular function terms was performed using the built-in STRING pipeline, and the top 10 terms were visualized based on strength (log_10_[observed/expected]). Enrichment parameters were set to a maximum false discovery rate (FDR) of 0.05, minimum signal of 0.01, and minimum strength of 0.01.

### Statistics

Statistical analyses were performed using Prism 10. All data are presented as mean ± SEM. No statistical method was used to predetermine sample size; it was empirically determined based on previous experiences or literature. Animals were genotyped before the experiment and treated uniformly. They were randomly allocated to each experimental condition, and data collection was conducted in a blind manner with different researchers performing animal preparations and experiments. For behavioral analysis, EthoVision and Freezeframe were used. For electrophysiology analysis, Clampfit (Molecular Devices), Minianalysis (Synaptosoft) and WinLTP (WinLTP), were used. For image analysis, ImageJ (NIH) and IMARIS (Bitplane) software were utilized. Statistical significance was set at *p < 0.05, **p < 0.01, ***p < 0.001, ****p < 0.0001.

### Reporting summary

Further information on research design is available in the Nature Portfolio Reporting Summary linked to this article.

## Supplementary information

Supplementary Information

Description of Additional Supplementary Files

Supplementary Data 1

Reporting Summary

Transparent Peer Review file

## Source data

Source data

## Data Availability

Additional data supporting the findings of this study are available from the corresponding author. Source data are provided with this paper.
