# Supplementary material for: Astrocytic ankyrin-2 enables memory persistence in the mouse hippocampus
Source: Nat Commun. 2026 Jul 7;17:5730. doi: 10.1038/s41467-026-75009-5 (PMC13342111; doi:10.1038/s41467-026-75009-5)
Supplement: Supplementary file 1 — Supplementary Information [file 41467_2026_75009_MOESM1_ESM.pdf]

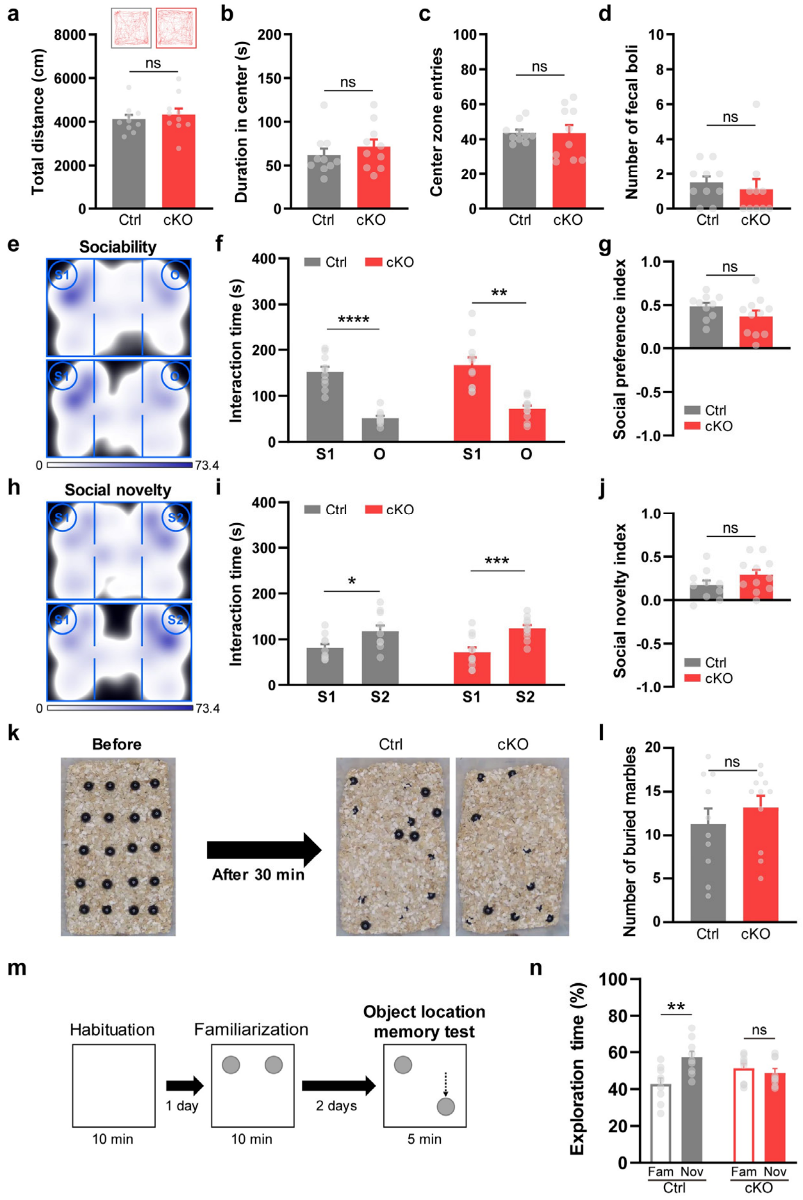

Supplementary Fig. 1. Behavioral characterization of astrocytic Ank2-deleted mice. (a-d) Open field test measuring general locomotion and anxiety-like behavior. (a) Total distance traveled (cm), (b) Time spent in the center zone (s), (c) Number of entries into the center zone, and (d) Number of fecal boli. Control group, n = 10 mice; Ank2^{GFAP} cKO group, n = 10 mice. (e-j) Three-chamber test assessing sociability and social novelty. (e) Representative heat map from the sociability test, where S1 represents the stranger mouse and O represents the empty wire cup. (f) Interaction time (s) with S1 and O. (g) Social preference index, calculated as (interaction time with S1 – interaction time with O) / (interaction time with S1 + interaction time with O). (h) Representative heat map from the social novelty test, where S1 refers to the previously encountered stranger mouse and S2 to a novel stranger mouse. (i) Interaction time (s) with S1 and S2. (j) Social novelty index, calculated as (interaction time with S2 – interaction time with S1) / (interaction time with S2 + interaction time with S1). Control group, n = 10 mice; Ank2^{GFAP} cKO group, n = 11 mice. (k, l) Repetitive behavior assessed using the marble-burying test. (k) images of the marble arrangement before (left) and after (right) the test. (l) Number of buried marbles. Control group, n = 10 mice; Ank2^{GFAP} cKO group, n = 11 mice. (m, n) Object Location Memory (OLM) test. (m) behavioral scheme of OLM. (n) Summary results of OLM. Control group, n = 9 mice; Ank2^{GFAP} cKO group, n = 9 mice. Data are mean ± SEM. *$P < 0.05$, **$P < 0.01$, ***$P < 0.001$, ****$P < 0.0001$. ns, not significant. Unpaired t-test (a, c, g, j), Mann-Whitney test (b, d, l), paired t-test (f, i), and Sidak's multiple comparisons test (n). All statistical tests were two-sided. Detailed statistics are provided in Supplementary Data 1. Source data are provided as a Source Data file.

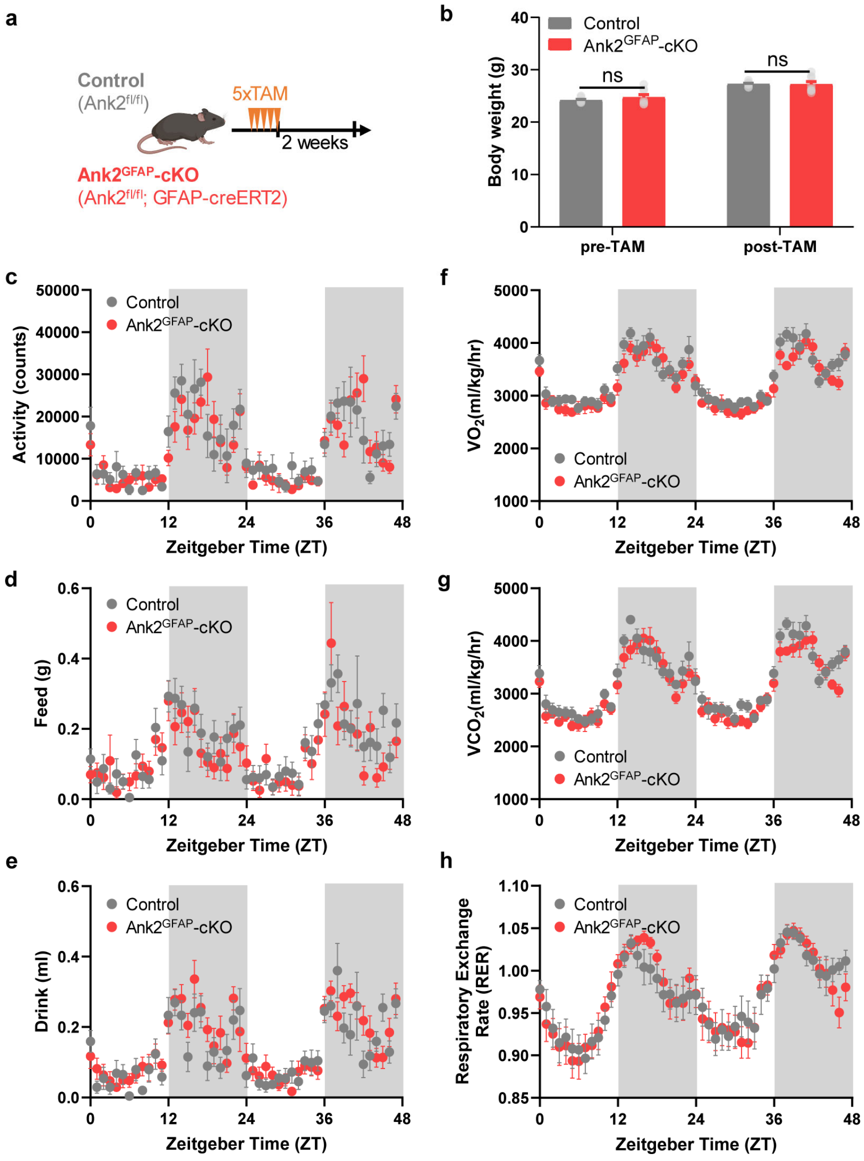

Supplementary Fig. 2. Metabolic and circadian measurements in astrocytic Ank2-deleted mice. (a) Preparation of control and Ank2^{GFAP} cKO mice. (b) Body weight comparison before (pre-TAM) and two weeks after tamoxifen injection (post-TAM). Control group, n = 7 mice; Ank2^{GFAP} cKO group, n = 8 mice. (c-h) Metabolic and circadian parameters measured using the Phenomaster metabolic cage system. Measurements were recorded over two consecutive days following a three-day habituation period in single-housed metabolic cages. (c) Locomotor activity, represented by the number of beam breaks. (d) Food consumption (gram). (e) Water intake (ml). (f) Oxygen consumption (VO_2, ml/kg/hr). (g) Carbon dioxide production (VCO_2, ml/kg/hr). (h) Respiratory exchange ratio (RER), calculated as VCO_2/VO_2. Data are mean ± SEM. ns, not significant. Mann-Whitney test (b). All statistical tests were two-sided. Detailed statistics are provided in Supplementary Data 1. (a) was created in BioRender. Koh, W. (2026) https://BioRender.com/4hltk5u. Source data are provided as a Source Data file.

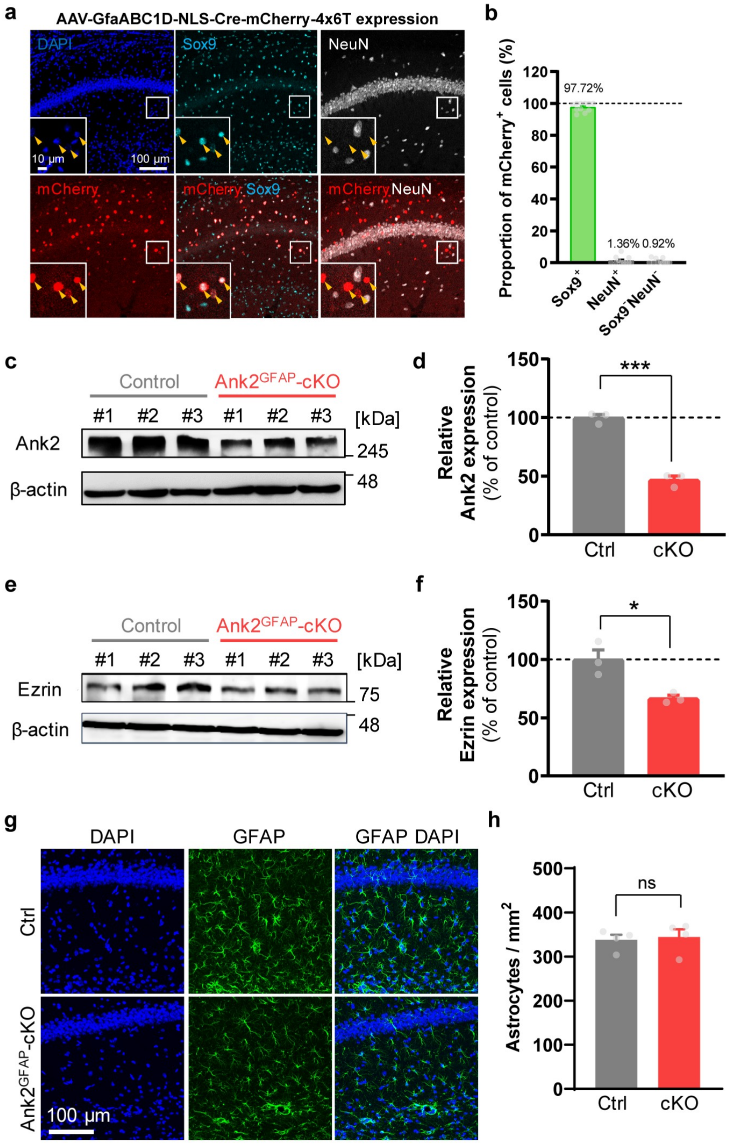

Supplementary Fig. 3. Specificity of AAV-GfaABC1D-NLS-Cre-mCherry-4x6T virus and quantification of Ank2 and Ezrin proteins as well as astrocyte number in the hippocampus. (a) Representative images of AAV-GfaABC1D-NLS-Cre-mCh-4x6T expression. Nuclei are stained with DAPI (blue), Cre-mCherry (red), astrocytes with Sox9 (green), and neurons with NeuN (gray). Yellow arrowheads indicate mCherry localization in Sox9-positive ($Sox9^+$) cells. (b) Quantification of $mCherry^+$ cells from 11 images across 4 mice. Dots indicate the proportion of $mCherry^+$ cells (%) in each image. Of 630 $mCherry^+$ cells, 618 were $Sox9^+$, 6 were $NeuN^+$, and 6 were negative for both Sox9 and NeuN. (c, d) Western blot analysis of Ank2 protein expression in the hippocampus of control and $Ank2^{GFAP}$ cKO mice. (c) Representative western blot image of Ank2 protein. (d) Quantification of relative Ank2 expression normalized to β-actin, followed by normalization to the control group. Control group, n = 3 mice; $Ank2^{GFAP}$ cKO group, n = 3 mice. (e, f) Western blot analysis of ezrin protein expression in the hippocampus of control and $Ank2^{GFAP}$ cKO mice. (e) Representative western blot image of ezrin protein. (f) Quantification of relative ezrin expression normalized to β-actin, followed by normalization to the control group. Control group, n = 3 mice; $Ank2^{GFAP}$ cKO group, n = 3 mice. (g, h) Assessment of astrocyte density in the hippocampal CA1 region. (g) Representative images of hippocampal CA1 astrocytes in control and $Ank2^{GFAP}$ cKO mice. (h) Quantified astrocyte density in control and $Ank2^{GFAP}$ cKO mice. Control group, n = 4 slices from 2 mice; $Ank2^{GFAP}$ cKO group, n = 4 slices from 2 mice. Data are mean ± SEM. *$P < 0.05$, ***$P < 0.001$. ns, not significant. Unpaired t-test with Welch's correction (d) and unpaired t-test (f, h). All statistical tests were two-sided. Detailed statistics are provided in Supplementary Data 1. Source data are provided as a Source Data file.

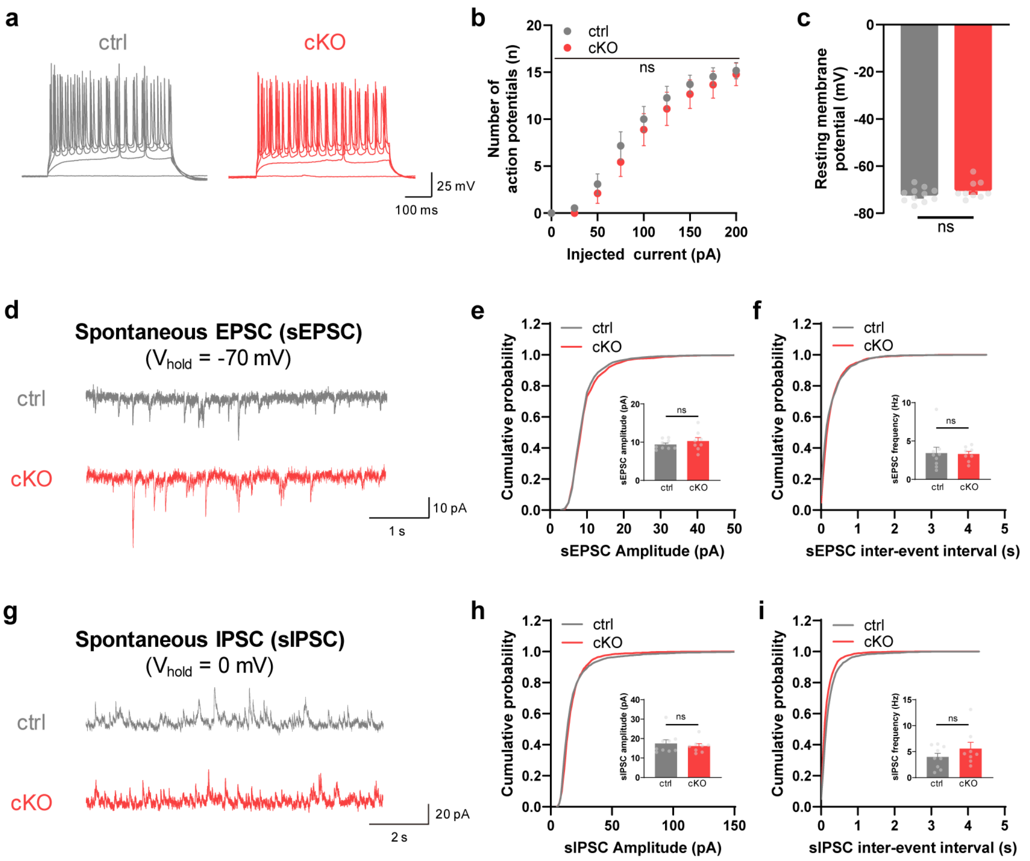

Supplementary Fig. 4. Basal synaptic properties of hippocampal CA1 pyramidal neurons assessed by whole-cell patch-clamp recording. (a) Representative traces of action potentials in CA1 pyramidal neurons from control and $Ank2^{GFAP}$ cKO mice in response to varying injected currents. (b) Summary of the number of action potentials induced at different current injection levels (pA). (c) Summary of resting membrane potential (mV). Control group, n = 11 CA1 pyramidal neurons from 2 mice; $Ank2^{GFAP}$ cKO group, n = 9 CA1 pyramidal neurons from 2 mice. (d) Representative traces of spontaneous excitatory postsynaptic currents (sEPSCs). (e) Summary of sEPSC amplitude. (f) Summary of sEPSC frequency. (g) Representative traces of spontaneous inhibitory postsynaptic currents (sIPSCs). (h) Summary of sIPSC amplitude. (i) Summary of sIPSC frequency. Control group, n = 9 CA1 pyramidal neurons from 2 mice; $Ank2^{GFAP}$ cKO group, n = 8 CA1 pyramidal neurons from 2 mice. Data are mean ± SEM. ns, not significant. Mann-Whitney test (b, f, h, i) and unpaired t-test (c, e). All statistical tests were two-sided. Detailed statistics are provided in Supplementary Data 1. Source data are provided as a Source Data file.

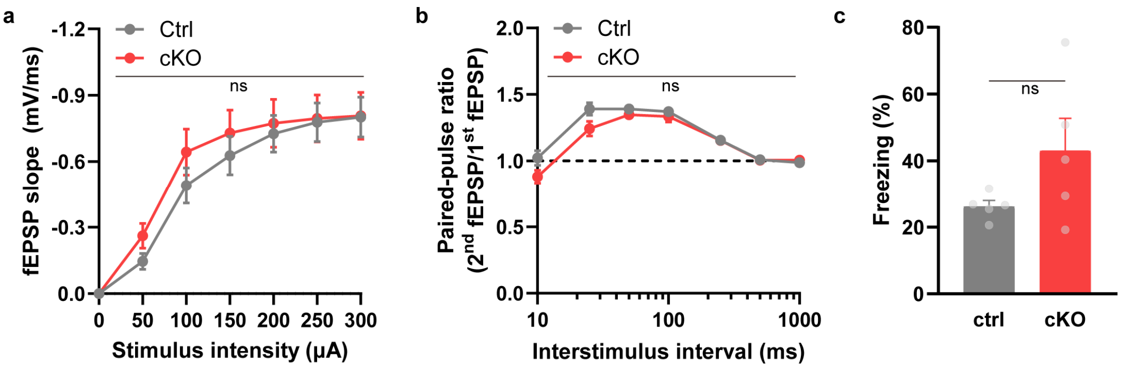

Supplementary Fig. 5. Basal synaptic properties in Ank2^{GFAP} cKO mice and recent memory performance in astrocyte-eGRASP experiments. (a) Input-output curve showing the field excitatory postsynaptic potential (fEPSP) slope from Schaffer collateral pathway in response to increasing stimulus intensities in control and Ank2^{GFAP} cKO mice. (b) Paired-pulse ratio (PPR) measured at inter-stimulus intervals of 10, 25, 50, 100, 250, 500, and 1000 ms in control and Ank2^{GFAP} cKO mice. Control group, n = 12 slices from 3 mice; Ank2^{GFAP} cKO group, n = 12 slices from 3 mice. (c) Freezing behavior during the recent memory test (Day 1) in control and Ank2^{GFAP} cKO mice used for astrocyte-eGRASP analysis in Fig. 4g-i. Astrocyte-eGRASP was assessed at this recent time point because the long-term stability of doxycycline-based eGRASP labeling has not yet been fully established[10]. Control group, n = 5 mice; Ank2^{GFAP} cKO group, n = 5 mice. Data are mean ± SEM. Unpaired t-test (a, b, c). All statistical tests were two-sided. Detailed statistics are provided in Supplementary Data 1. Source data are provided as a Source Data file.

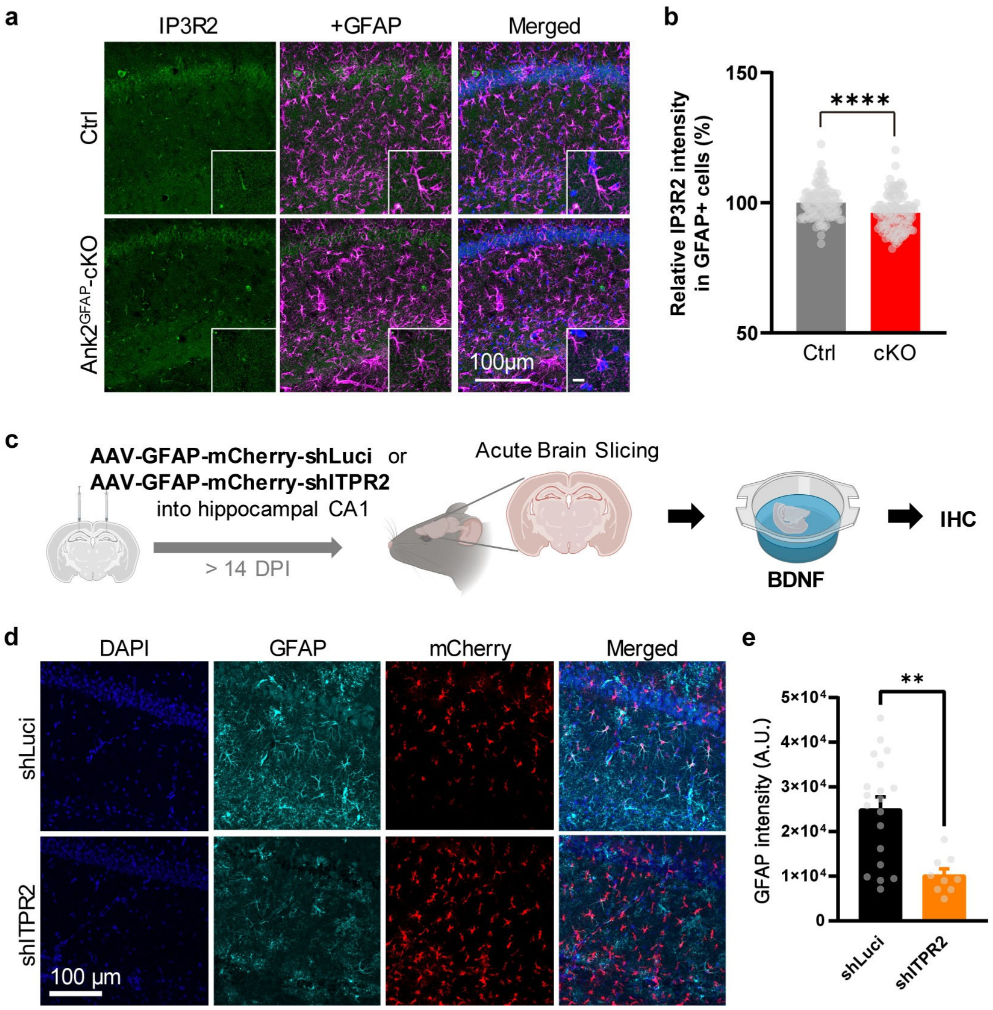

Supplementary Fig. 6. Involvement of IP3R2 in BDNF-dependent astrocyte morphogenesis and validation of BDNF-induced astrocyte morphogenesis *in vivo*. (a) Representative images of IP3R2 staining in control and Ank2^{GFAP} cKO mice. (b) Quantified IP3R2 intensity in GFAP^{+} cells. Control group, n = 101 astrocytes from 4 mice; Ank2^{GFAP} cKO group, n = 109 astrocytes from 4 mice. (c) Schematic diagram illustrating the investigation of IP3R2 involvement in BDNF-dependent astrocyte morphogenesis. (d) Representative images of GFAP staining in brain slices from mice injected with either AAV-GFAP-mCherry-shLuci (control) or AAV-GFAP-mCherry-shITPR2, following BDNF treatment. (e) Comparison of GFAP intensity between shLuci and shITPR2 groups after BDNF treatment. shLuci group, n = 19 astrocytes from 2 mice; shITPR2 group, n = 9 astrocytes from 2 mice. Data are mean ± SEM. $^{**}P < 0.01$, $^{****}P < 0.0001$. Unpaired t-test (b, e). All statistical tests were two-sided. Detailed statistics are provided in Supplementary Data 1. (c) was created in BioRender. Koh, W. (2026) https://BioRender.com/4hltk5u. Source data are provided as a Source Data file.

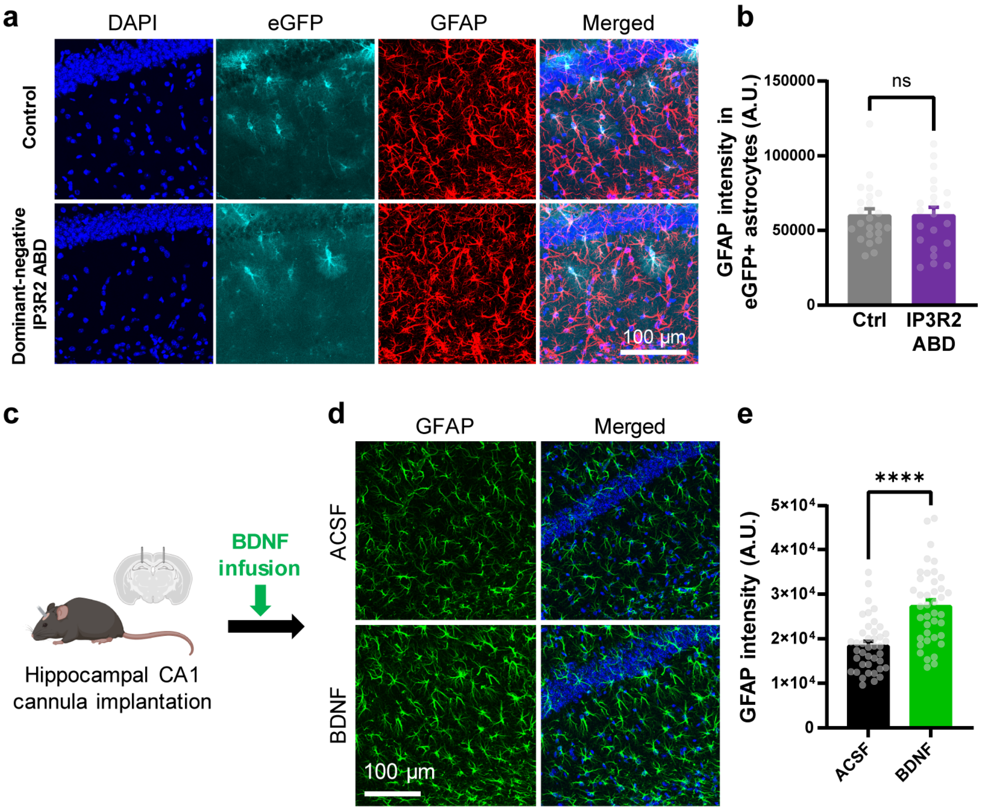

Supplementary Fig. 7. Expression of dominant-negative IP3R2 Ankyrin-binding domain (ABD) in hippocampal astrocytes and BDNF-induced hippocampal astrocyte morphogenesis. (a) Representative images of AAV-GFAP-eGFP-P2A-IP3R2 ABD and control, AAV-GFAP-eGFP virus injected mice. (b) GFAP intensity in eGFP+ astrocytes. Control group, n = 23 astrocytes from 2 mice; IP3R2 ABD group, n = 22 astrocytes from 2 mice. (c) Schematic of hippocampal CA1 cannula implantation, performed at least one week before infusion. (d) Representative images of GFAP staining following ACSF or BDNF (100 ng/0.4 µL ACSF/side) infusion. (e) Comparison of GFAP intensity between ACSF- and BDNF-infused mice. ACSF group, n = 41 astrocytes from 2 mice; BDNF group, n = 40 astrocytes from 2 mice. Data are mean ± SEM. ****$P < 0.0001$. Welch's t-test (b) and unpaired t-test (e). All statistical tests were two-sided. Detailed statistics are provided in Supplementary Data 1. (c) was created in BioRender. Koh, W. (2026) https://BioRender.com/4hltk5u. Source data are provided as a Source Data file.

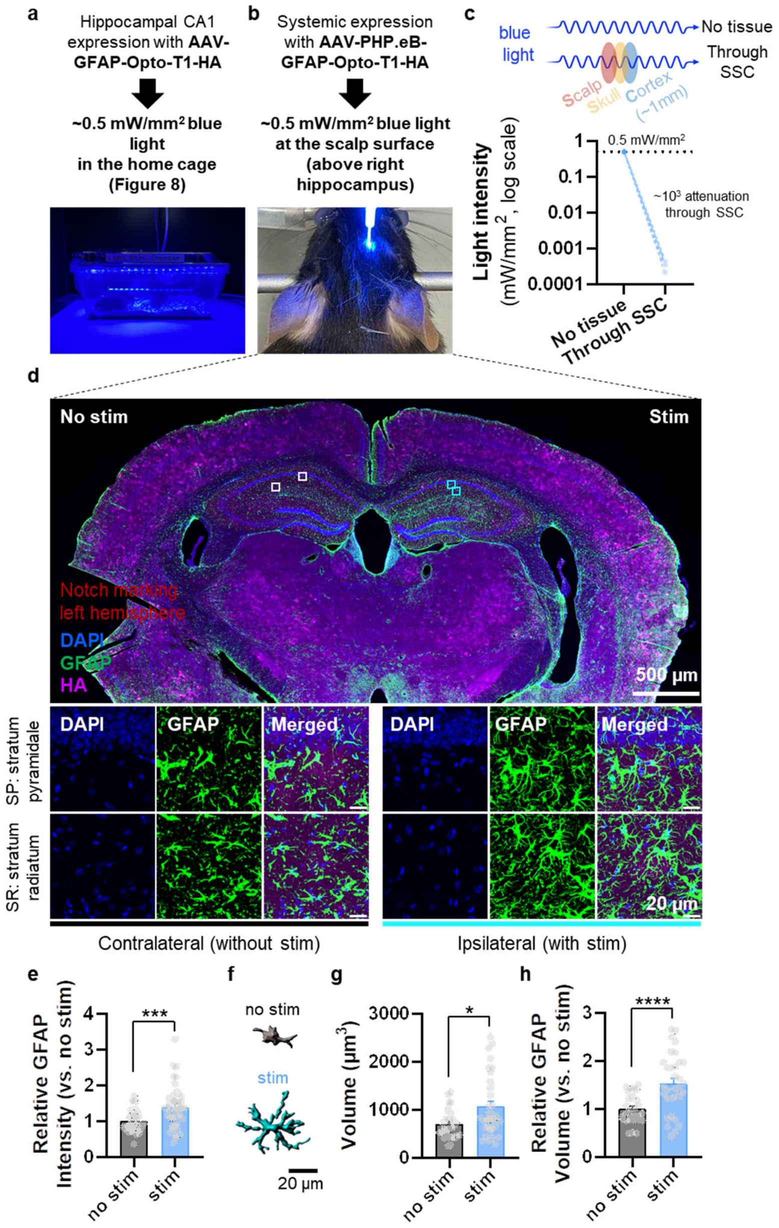

Supplementary Fig. 8. Non-invasive blue light stimulation (0.5 mW/mm^2) is sufficient to activate Opto-T1 through the intact scalp, skull, and cortex (SSC). (a) Non-invasive blue light stimulation setup in the home cage (0.5 mW/mm^2 through a custom LED lid) used in Figure 8. (b) To directly test light penetration, Opto-T1 was broadly expressed via retro-orbital AAV-PHP.eB-GFAP-Opto-T1-HA. Three weeks later, the blue light (473 nm, 0.5 mW/mm^2, 10 min) was applied to the scalp above the right hemisphere in anesthetized mice. The mice were sacrificed 1 h after stimulation (the left hemisphere was notched for identification after fixation). (c) Blue light transmission through overlying tissues was measured by placing cadaver-derived scalp, skull, and cortex (SSC) above the photodetector. Irradiance decreased by approximately three orders of magnitude (10^3) when light passed through SSC compared to the no-tissue condition. (d) GFAP immunostaining revealed robust activation only in the illuminated hemisphere, demonstrating that 0.5 mW/mm^2 blue light is sufficient to activate Opto-T1 through the intact scalp and reach hippocampal CA1. (e) Relative GFAP intensity normalized to the no stimulation (no stim) condition. n = 35 (no stim) and 44 (stim) cells; 7 slices from 3 mice. (f) Representative GFAP volume rendering using IMARIS. (g) GFAP volume. (h) Relative GFAP volume (normalized to no stim). n = 29 (no stim) and 32 (stim) cells; 7 slices from 3 mice. Data are mean ± SEM. **P < 0.01, ***P < 0.001, ****P < 0.0001. Mann-Whitney test (e, g) and unpaired t-test (h). All statistical tests were two-sided. Detailed statistics are provided in Supplementary Data 1. (c) was created in BioRender. Koh, W. (2026) https://BioRender.com/4hltk5u. Source data are provided as a Source Data file.

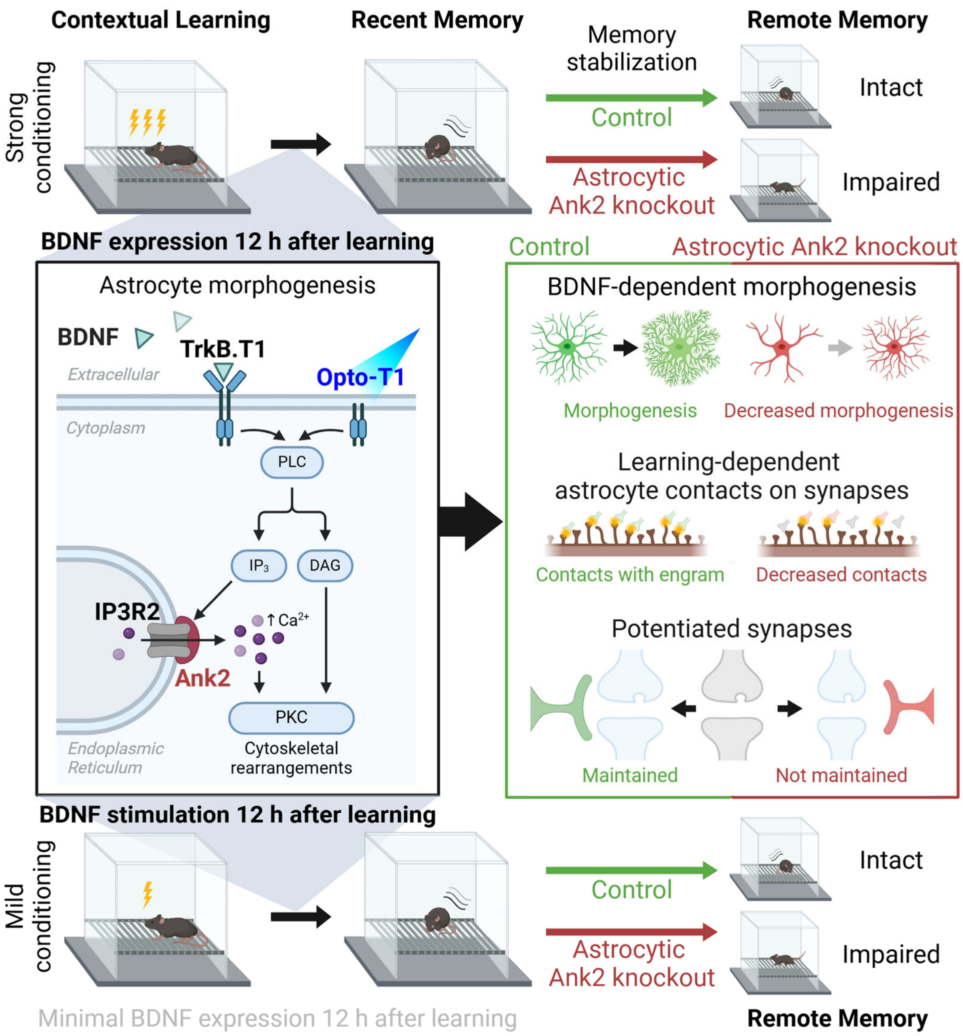

Supplementary Fig. 9. Graphical summary of the role of astrocytic Ank2 in remote memory function. Astrocytic Ank2 sustains IP3R2 expression and function within the BDNF-TrkB.T1-IP3R2 signaling axis, which is engaged ~12 hours after learning, a critical window for memory persistence. Through this pathway, BDNF induces cytoskeletal rearrangements and astrocyte morphogenesis. The resulting structural plasticity promotes astrocytic contacts with engram neurons, thereby stabilizing potentiated synapses and ensuring long-term memory persistence. Opto-T1 activation further shows that astrocytic TrkB.T1 signaling alone is sufficient to enhance memory persistence in the hippocampus. Created in BioRender. Koh, W. (2026) https://BioRender.com/4hltk5u.
