# Supplementary material for: Astrocytic ankyrin-2 enables memory persistence in the mouse hippocampus
Source: Nat Commun. 2026 Jul 7;17:5730. doi: 10.1038/s41467-026-75009-5 (PMC13342111; doi:10.1038/s41467-026-75009-5)
Supplement: Supplementary file 2 — Description of Additional Supplementary Files [file 41467_2026_75009_MOESM2_ESM.pdf]

Description of Additional Supplementary Files

Supplementary Data 1 Statistical analysis summary
