# Supplementary material for: Astrocytic ankyrin-2 enables memory persistence in the mouse hippocampus
Source: Nat Commun. 2026 Jul 7;17:5730. doi: 10.1038/s41467-026-75009-5 (PMC13342111; doi:10.1038/s41467-026-75009-5)
Supplement: Supplementary file 4 — Reporting Summary [file 41467_2026_75009_MOESM4_ESM.pdf]

nature portfolio

Corresponding author(s): Wuhyun Koh

Last updated by author(s): Jun 6, 2026

Reporting Summary

Nature Portfolio wishes to improve the reproducibility of the work that we publish. This form provides structure for consistency and transparency in reporting. For further information on Nature Portfolio policies, see our Editorial Policies and the Editorial Policy Checklist.

Statistics

For all statistical analyses, confirm that the following items are present in the figure legend, table legend, main text, or Methods section.

n/a	Confirmed	
☐	☒	The exact sample size (*n*) for each experimental group/condition, given as a discrete number and unit of measurement
☐	☒	A statement on whether measurements were taken from distinct samples or whether the same sample was measured repeatedly
☐	☒	The statistical test(s) used AND whether they are one- or two-sided<br>*Only common tests should be described solely by name; describe more complex techniques in the Methods section.*
☒	☐	A description of all covariates tested
☐	☒	A description of any assumptions or corrections, such as tests of normality and adjustment for multiple comparisons
☐	☒	A full description of the statistical parameters including central tendency (e.g. means) or other basic estimates (e.g. regression coefficient) AND variation (e.g. standard deviation) or associated estimates of uncertainty (e.g. confidence intervals)
☐	☒	For null hypothesis testing, the test statistic (e.g. *F*, *t*, *r*) with confidence intervals, effect sizes, degrees of freedom and *P* value noted<br>*Give P values as exact values whenever suitable.*
☒	☐	For Bayesian analysis, information on the choice of priors and Markov chain Monte Carlo settings
☒	☐	For hierarchical and complex designs, identification of the appropriate level for tests and full reporting of outcomes
☒	☐	Estimates of effect sizes (e.g. Cohen's *d*, Pearson's *r*), indicating how they were calculated

Our web collection on statistics for biologists contains articles on many of the points above.

Software and code

Policy information about availability of computer code

Data collection	Confocal images were captured using an LSM 900 (Zeiss) microscope with ×20/0.8 NA (air) and ×63/1.4 NA (oil) objectives, as well as a Nikon A1R MP microscope. In electrophysiology experiments, electrical data were digitized and recorded using a Digidata 1440A system and an Axon Multiclamp 700B patch-clamp amplifier (Molecular Devices, CA, USA). Field excitatory postsynaptic potentials (fEPSPs) were measured with a DAM-80 amplifier and WinLTP software. Cellular morphology was visualized using an upright microscope (Examiner D1; Zeiss, Germany) and a CMOS camera (ORCA-Flash 4.0; Hamamatsu, Japan).
Data analysis	Confocal imaging data was analyzed using Imaris (9.9.0 and 10, Oxford Instruments), FIJI (ImageJ), and Nikon Imaging Software (NIS Elements AR 64-bit version 3.21). General behavior was recorded and analyzed using EthoVision XT (Noldus). Fear conditioning experiments were conducted in a fear conditioning chamber (Coulbourn Instruments), with data recorded and analyzed using FreezeFrame4 software. Electrophysiology data was analyzed using pClamp 10.7 or WinLTP software. Excitatory and inhibitory postsynaptic currents were detected and analyzed using MiniAnalysis (synaptosoft).

For manuscripts utilizing custom algorithms or software that are central to the research but not yet described in published literature, software must be made available to editors and reviewers. We strongly encourage code deposition in a community repository (e.g. GitHub). See the Nature Portfolio guidelines for submitting code & software for further information.

Data

Policy information about availability of data

All manuscripts must include a data availability statement. This statement should provide the following information, where applicable:

- Accession codes, unique identifiers, or web links for publicly available datasets
- A description of any restrictions on data availability
- For clinical datasets or third party data, please ensure that the statement adheres to our policy

All data generated in this study should be provided in the Source Data file.

Research involving human participants, their data, or biological material

Policy information about studies with human participants or human data. See also policy information about sex, gender (identity/presentation), and sexual orientation and race, ethnicity and racism.

Reporting on sex and gender	Not applicable
Reporting on race, ethnicity, or other socially relevant groupings	Not applicable
Population characteristics	Not applicable
Recruitment	Not applicable
Ethics oversight	Not applicable

Note that full information on the approval of the study protocol must also be provided in the manuscript.

Field-specific reporting

Please select the one below that is the best fit for your research. If you are not sure, read the appropriate sections before making your selection.

☒ Life sciences ☐ Behavioural & social sciences ☐ Ecological, evolutionary & environmental sciences

For a reference copy of the document with all sections, see nature.com/documents/nr-reporting-summary-flat.pdf

Life sciences study design

All studies must disclose on these points even when the disclosure is negative.

Sample size	No statistical method was used to predetermine the sample size. Instead, sample sizes were determined based on previous experience or a review of similar experiments in the literature.
Data exclusions	Mice were excluded from the experiments if the surgery was unsuccessful due to virus expression in a non-targeted region.
Replication	To ensure the reproducibility of the experimental findings, data from both cell (electrophysiology, calcium imaging) and animal experiments were collected across multiple trials. The sample sizes, including the number of cells and animals used, are provided in the respective figure legends.
Randomization	For the behavioral tests (including those involving virus-injected mice), age-matched male mice were randomly assigned to groups. For other experiments, both male and female mice were randomly assigned to groups while matching for both age and sex.
Blinding	To conduct behavioral experiments in a blinded manner, different researchers were responsible for experiments and data analysis. In imaging analysis (astrocyte morphology and astrocyte-eGRASP), researchers were not informed of the genotype until they completed the analysis.

Reporting for specific materials, systems and methods

We require information from authors about some types of materials, experimental systems and methods used in many studies. Here, indicate whether each material, system or method listed is relevant to your study. If you are not sure if a list item applies to your research, read the appropriate section before selecting a response.

Materials & experimental systems

n/a	Involved in the study	
☐	☒	Antibodies
☒	☐	Eukaryotic cell lines
☒	☐	Palaeontology and archaeology
☐	☒	Animals and other organisms
☒	☐	Clinical data
☒	☐	Dual use research of concern
☒	☐	Plants

Methods

n/a	Involved in the study	
☒	☐	ChIP-seq
☒	☐	Flow cytometry
☒	☐	MRI-based neuroimaging

Antibodies

Antibodies used	For immunohistochemistry, primary and secondary antibodies used are as follow: chicken anti-GFAP (1:500, AB5541, Millipore), rabbit HA-Tag (1:500, 3724S, Cell signaling), guinea pig anti-NeuN (1:500, ABN90, Millipore), donkey anti-chicken IgG Alexa Fluor 488 (1:500, 703-545-155, Jackson), donkey anti-chicken IgG Alexa Fluor 594 (1:500, 703-585-155, Jackson), donkey anti-chicken IgG Alexa Fluor 647 (1:500, 703-605-155, Jackson), donkey anti-guinea pig IgG Alexa Fluor 647 (1:500, 706-605-148, Jackson).<br>For western blotting, primary and secondary antibodies used are as follow: rabbit anti-Ank2 antibody (75-671 145, NeuroMab), rabbit anti-Ezrin antibody (3145S, Cell Signaling), rabbit anti-β-actin antibody (ab133626, Abcam), anti-rabbit HRP (Pierce, 170-6515).
Validation	Anti-Ank2 was validated in this paper using Ank2 cKO mice. Anti-GFAP, anti-NeuN, and anti-β-actin were validated previous studies including (Kang, et al., Nat Commun 15, 5830 (2024).

Animals and other research organisms

Policy information about studies involving animals; ARRIVE guidelines recommended for reporting animal research, and Sex and Gender in Research

Laboratory animals	C57BL/6J mice, Ank2 (fl/fl): GFAP-creERT2 mice (experimental group, astrocyte-specific conditional knockout) and Ank2 (fl/fl) mice (control group), aged 8 to 25 weeks, were used in this study. They were maintained on a 12-hour light/dark cycle with free access to food and water.
Wild animals	Not applicable
Reporting on sex	Both male and female mice were utilized in the study, with only males being used for behavioral tests.
Field-collected samples	Not applicable
Ethics oversight	All experimental procedures were approved by the Institutional Animal Care and Use Committee (IACUC) of the Institute for Basic Science (IBS, Republic of Korea). Surgical procedures were performed in compliance with IBS IACUC guidelines.

Note that full information on the approval of the study protocol must also be provided in the manuscript.

Plants

Seed stocks	Not applicable
Novel plant genotypes	Not applicable
Authentication	Not applicable
