# Supplementary material for: Astrocytic ankyrin-2 enables memory persistence in the mouse hippocampus
Source: Nat Commun. 2026 Jul 7;17:5730. doi: 10.1038/s41467-026-75009-5 (PMC13342111; doi:10.1038/s41467-026-75009-5)
Supplement: Supplementary file 5 — Transparent Peer Review file [file 41467_2026_75009_MOESM5_ESM.pdf]

nature portfolio

Peer Review File

Astrocytic Ankyrin-2 Enables Memory Persistence in the Mouse Hippocampus

Corresponding Author: Dr Wuhyun Koh

This file contains all reviewer reports in order by version, followed by all author rebuttals in order by version.

Attachments originally included by the reviewers as part of their assessment can be found at the end of this file.

Version 0:

Reviewer comments:

Reviewer #1

(Remarks to the Author)
The manuscript provides significant new insights into the role of astrocytic Ankyrin-2 (Ank2) in memory persistence. The authors demonstrate that astrocyte-specific deletion of Ank2 impairs remote memory without affecting recent memory, reduces astrocyte morphological complexity, impairs long-term potentiation (LTP) maintenance, and reduces astrocytic participation in engram. Mechanistically, Ank2 interacts with IP3R2, regulates astrocyte morphogenesis, and memory persistence. Furthermore, selective activation of astrocytic BDNF signaling via optogenetics enhances remote memory, highlighting the astrocytic contribution to cognitive functions.

The work addresses an important and understudied aspect of astrocyte biology related to cognitive functions. The authors comprehensively use molecular genetics, electrophysiology, imaging techniques, and behavioral assays. They also applied innovative tools (e.g. astrocyte-eGRASP and Opto-cytTrkB.T1) that strengthen the impact of the study. This work provides clear demonstration of the necessity and sufficiency of astrocytic signaling in memory persistence.

Comments

1. The rationale for selecting GFAP-creERT2 over Aldh1l1-creERT2 lacks robustness. GFAP is heterogeneously expressed in astrocytes and other cell types, which might confound specificity. ALDH1L1 is a more uniformly expressed astrocyte marker. The authors should provide a more substantial justification or clarify potential limitations explicitly in the manuscript.

2. "Astrocyte-engram neuron contact" usage can be confusing. Both astrocytes and neurons can form an engram (https://doi.org/10.1038/s41586-024-08170-w; https://doi.org/10.1016/j.tins.2021.07.006). Consider explicitly mentioning "astrocytic component of an engram" to avoid ambiguity.

3. "PAPs" is also confusing since this abbreviation can refer to both "Perisynaptic Astrocytic Processes" and "Peripheral Astrocytic Processes" (approx. 50% each). Specifically, the authors appear to mean astrocytic leaflets (fine, organelle-free protrusions), not thicker organelle-containing astrocytic branches, which can also be perisynaptic (https://doi.org/10.3389/fncel.2018.00248). It is recommended to clearly define and consistently use "leaflets" in the text (https://doi.org/10.1016/j.tcb.2023.09.006).

4. Reduced ezrin expression and GFAP changes observed in Ank2 cKO mice conflict with previous literature showing inverse relationships under pathological conditions. Previous studies have shown that ezrin deficiency leads to upregulation of GFAP expression (https://doi.org/10.1002/glia.24253), and increased GFAP with reduced ezrin expression is correlated with astrocytic atrophy in aging and conditions such as Alzheimer's disease and depression. Address this discrepancy and discuss potential underlying mechanisms.

5. The binding of Ank2 to IP3R2 and its role in astrocytic calcium signaling are primarily correlative. Direct functional experiments demonstrating Ank2's influence on IP3R2-mediated calcium dynamics are necessary to establish causality. Astrocytic atrophy and reduced communication with neurons may be the primary reasons for decreased calcium activity in astrocytes in Ank2 cKO mice. The authors should clearly state this limitation.

6. Potential alternative explanations for impaired LTP (such as quicker deterioration of slices in Ank2 cKO mice due to reduced astrocytic metabolic support) should be considered and experimentally addressed or at least thoroughly discussed. This is important given that astrocytic shrinkage can impair metabolic support, potentially leading to faster tissue deterioration ex vivo.

Reviewer #2

(Remarks to the Author)
Overall Assessment:
This manuscript utilizes transgenic mice or Cre-virus mediated astrocyte-specific knockdown of Ank2, demonstrating that conditional knockout (cKO) mice exhibit impaired remote memory recall, reduced astrocytic morphology, decreased synaptic interactions leading to impaired synaptic plasticity and LTP. These effects are mediated through the BDNF-TrkB.T1-IP3R2 Ca2+ signaling pathway. The finding in this study about astrocytes Ank2 specifically in remote memory is interesting, adding valuable insights for the field. While the study presents a logically coherent narrative with reasonable experimental design (particularly the innovative Opto-T1 tool), several critical issues need to be addressed prior to publication.

Major Concerns:
1. The authors employ GFAP-creERT2 mice for astrocyte-specific Ank2 knockout. While cited reference #32 reports high astrocyte specificity (>80%) but relative low astrocyte coverage (50-60%) in hippocampal CA1 and dentate gyrus with this line, other reviews note that GFAP-Cre/ERT2 exhibits low expression in astrocytes relative to other cell types （Yu X, Nagai J, Khakh BS. Improved tools to study astrocytes. Nat Rev Neurosci. 2020 Mar;21(3):121-138）. Although WB shows ~50% Ank2 reduction, this was performed on whole hippocampus CA1 tissue lysates without astrocyte sorting. Additional validation of expression efficiency and specificity of Cre recombinase, and Ank2 knockdown efficiency in astrocytes is required, such as: immunofluorescence co-staining (providing spatial distribution data) or WB analysis of sorted astrocytes.
2. The co-localization rate of Cre-mCherry with GFAP in Fig 1J appears suboptimal. Quantitative analysis of mCherry/GFAP co-labeling efficiency and Ank2 knockdown efficiency in astrocytes should be provided to confirm targeting specificity.
3. Figs 2I-2K analyze only 7-9 astrocytes - this sample size is statistically inadequate. While cell numbers can serve as n-values for morphological analysis, the number of animals used should also be reported.
4. The astrocyte-eGRASP signal (Fig 4) infers synaptic coverage changes, and the Graphical summary (Supplementary Fig. 7) suggests reduced astrocytic process envelopment of synapses. However, Electron microscopy demonstrating tripartite synapse structural changes or advanced imaging tools (such as NAPA, Octeau JC, Chai H, Jiang R, Bonanno SL, Martin KC, Khakh BS. An Optical Neuron-Astrocyte Proximity Assay at Synaptic Distance Scales. Neuron. 2018 Apr 4;98(1):49-66.e9.) is recommended to claim this issue more sufficiently.
5. The proposed mechanism involves Ank2 knockdown → impaired IP3R2 function → reduced Ca2+ signaling → morphological/functional deficits. Does IP3R2 activation or astrocytic Ca2+ elevation rescue astrocyte morphology, tripartite synapse structure, synaptic plasticity or behavioral deficits in cKO mice?
6. Since BDNF infusion and Opto-T1 activation modify astrocyte morphology and memory persistence, comparative data such as astrocytic Ca2+ signaling, LTP, astrocyte-neuron contacts under both BDNF and Opto-T1 conditions between cKO and ctrl should be provided to strengthen the mechanistic chain.
7. Supplementary Fig. 4d/g exhibits an unstable baseline (notably for IPSCs), and a discrepancy exists between trace scale bars and histogram frequencies (Supplementary Fig. 4f/i): the ~5s trace in panel d displays ~10 EPSCs, inconsistent with the reported ~4Hz mean frequency.
8. The high background in Fig 8C HA staining compromises astrocyte morphology visualization, necessitating higher magnification images and co-localization analysis (HA/astrocyte/neuron markers) to confirm viral infection efficiency and targeting specificity.
9. Sholl analysis revealed a reduction in total branch length but no significant change in ending radius, warranting further discussion on its potential impact on synaptic coverage.

Minor Concerns:
1. Fig 1j schematic: Remove space in "2 w eeks" → "2 weeks".
2. Supplementary Figure 1 shows inconsistent font/axis formatting among panels a-d and others. Remove '**' from legends where absent in panels (apply to all legends).
3. Fig 7e: The Y-axis scale obscures data resolution. Please consider reducing the range.
4. Line 562: Add negative sign to "-70mV".
5. The panel labeling is inconsistent across figures, with lowercase letters (a, b, c, d) used in Figures 1 and 2, while uppercase letters (A, B, C, D) appear in Figure 3.
6. The P-values in Figure 2's figure caption are italicized, while those in other figure captions are not. Please standardize the formatting of all P-values across figures to comply with journal guidelines.
7. In Fig.5, “n=433” should be “n = 433”.
8. The term “GFAP-creERT2” should be uniformly corrected to “GFAP-CreERT2” (title case) throughout the manuscript.

Reviewer #3

(Remarks to the Author)
While contextual fear memories are known to depend on neurons in the hippocampal formation, much less is known about the role of astrocytes in the formation and maintenance of memories. Hayoung et al. have used a wide range of experimental

methods to demonstrate that the cytoskeletal adapter protein Ank2 supports BDNF-mediated structural plasticity in astrocytes that is required and sufficient for remote memory expression.
While their work is technically of a high standard, encompasses a wide range of methods, and convincingly demonstrates links between Ank2 and the structural integrity of astrocytes, the links between Ank2 and BDNF as well as Ank2 and memory persistence currently appear weak and require further evidence to support the authors' claims.
If further evidence can be provided, the identification of a mechanistic link between cytoskeletal proteins in astrocytes supporting memory persistence in the hippocampus would be an important discovery to warrant publication in Nature Communications.

Major:
1. The authors' main argument rests on their finding of impaired remote memory performance after conditional Ank2 deletion in CA1 astrocytes (Fig. 1m). Where is Fig. 1n on memory persistence that is referred to in the legend? Is persistence affected? If not, why not? Were these injections performed bilaterally or unilaterally? – the figure legend/methods remain unclear on this. Why were only 9/8 mice tested here? The statistically differences are significant but barely so. They are definitively much weaker than those shown in Fig. 1f, which begs the question if there are other astrocytic populations that play a more important role? The authors should strengthen their claims by either consolidating their results with additional animals or identify other targets outside CA1 that could explain the clear behavioural phenotype seen in Fig. 1f.
2. The relationship of BDNF with Ank2 appears rather weak and requires further data. For example, while the authors suggest an impaired "volume change (Δ Volume)" in cKO animals after BDNF treatment (Fig. 6d-e), it is unclear why the authors would quantify absolute differences and not proportional differences. Indeed, comparing Fig. 6c and 6d, it appears that booth ctrl and cKO volumes approximately double in volume after BDNF treatment, which suggests that cKO astrocytes are still responding the same way to BDNF as controls, despite their lower baseline. Furthermore, in Fig. 7, the authors claim that "BDNF-induced memory persistence is reduced in astrocyte-specific Ank2-deleted mice", but the authors only compare control+vehicle, control+BDNF, and cKO+BDNF, but not the criticial cKO+vehicle. Hence, it remains unclear what the baseline cKO level is that any BDNF effects would need to be compared to.
3. I remain unconvinced of the efficacy of the Opto-T1 construct. Since the authors newly developed this construct and show only a single quantitative panel featuring an unknown number of mice, further (in vitro) data is required to establish this tool as functional. I am furthermore surprised to see this construct improving memory persistence. Why is the memory persistence of controls in Fig. 1g >90%, while it is ~60% in Fig. 8i? Is this due to different shock protocols? If yes, the authors should also show the data with congruent shock protocols (3 shocks in Fig. 8; 1 shock in Fig. 1). Furthermore, to demonstrate the currently missing link to Ank2, the authors should aim to rescue the memory phenotype produced by Ank2-GFAP-cKO with the activation of opto-T1 in the hippocampus.
4. The authors need to explain the link between the visible decline of Ank2GFAP-cKO in L-LTP starting at 2 hours after LTP induction (Fig. 4b) and their behavioural results indicating no memory deficits 24 hours after conditioning (Fig. 1e). How do the authors reconcile the fact that LTP seems impaired already after 2 hours, while behavioural phenotypes remain intact for at least 24 hours? The authors need to discuss this point or produce further evidence to reconcile these results.
5. Why was eGRASP used in mice that underwent conditioning and memory tests if no behavioural results are shown (Fig. 4c-g)? The authors should show if memory expression levels are correlated with eGRASP density, as would be expected. If not, the authors need to explain this or adjust their model.
6. The authors claim "Ank2 as a key regulator of IP3R2 function" (l. 269-270), referring only to one panel of co-immunoprecipitation (Fig. 5a) and a single article published in 2022 in Molecular Psychiatry. The data in Fig. 5b-h shows an impairment in calcium dynamics that could be the result of other cellular processes. The authors should either tone down this statement or find further evidence for this.
Minor points:
• The author should consider changing the title, which is more specific to the fear memory persistence. For example, memory can be classified as aversive and pleasant memories, explicit and implicit memories, or long-term and short-term memories. If the authors give the paper a title, "Astrocytic Ankyrin2 Enables Memory Persistence", the experiments must be designed to investigate various types of memories; alternatively, the authors may want to be more specific in their title.
• In Fig. 7e, the authors show context specificity for the memory tested. The authors should show such specificity also for the data presented in Fig. 1 to rule out indiscriminate anxiety.
• The Cre-LoxP system is a widely used tool for genetic modification in mouse models. However, the specificity of the virus needs to be quantified and shown in the paper. For example, the percentage of colocalization between astrocytes and the viral marker. Will hippocampus-specific deletion of astrocytic Ank2 decrease the number or volume of astrocytes? The images in Fig. 1.j are not enough to show the specificity. Please give the calculation results and add a higher magnification of the colocalized examples.
• The authors did not describe the experimental steps for contextual fear conditioning in the hippocampal BDNF diffusion groups.
• In Fig.7 and Fig. 8.f-j, the authors used a similar protocol to generate fear memory in mice. However, the freezing time for the control groups is different (BDNF control group: ~27%, optic control group: ~50%) in the 1 day after conditioning contextual tests. The authors should discuss the potential reason in the discussion part or explain it with references.
• The authors need to list all statistical results for every figure. For example, the author should apply a suitable statistical analysis between groups (e.g., two-way ANOVA) for Fig. 1.d, h, and k, Fig. 2.d, and Supple. 5a.
• The authors should provide an overview image of injection and implantation sites in the mouse brain for each mouse.
• What does the abbreviation "> 14 DPI" mean in Fig. 8.b and Supplementary Fig. 6.a?
• The author should describe the laser delivery strategy in the methods part. How long is the laser stimulus? Is that pulsed or continuous stimulation? What is the wavelength used for the blue light?
• In Fig. 2, Fig. 3, Fig. 4, Fig. 5, Fig. 6, and Fig. 8.e, the authors need to show the number of mice in the groups and calculate the mean value from each mouse instead show the whole group of data was gathered from slices, astrocytes, soma, or microdomains. It is less convincing if the data came from one or two mice.

• The authors used similar schematic figures to show the virus injection in Fig. 1.j, Fig. 4.d, Fig. 5.b, Fig. 8.b. The authors need to make the figures clear enough for the reader to understand their experimental design. Please show the unilateral and bilateral injections clearly in the figures.
• Please replace Supplementary Figure 3.c with a higher resolution image. (The result of the control #1 looks a bit artificial.)
• Please do not compress the data points in Fig. 7.e and Fig. 8.j. The authors need to find a suitable y axis range to show the data clearly in the figures.
• In Supplementary Fig. 7, the authors wrote: “Astrocytic Ank2 plays a distinct role in maintaining IP3R2 expression and function within the TrkB.T1-IP3R2 axis, which is essential for astrocyte morphogenesis via cytoskeletal rearrangements”. However, it is not consistent with the figure described as IP3Rs. The authors need to summarize the figure and consistently provide a description.

Version 1:

Reviewer comments:

Reviewer #1

(Remarks to the Author)
The authors thoroughly addressed all my comments.

Reviewer #2

(Remarks to the Author)
The authors have done a good revision to address my concerns. Now I don't have any major concerns regarding the current form of the manuscript. The only thing that I still would like to note is that : they stated in the response letter point 5 that "In addition, because we observed reduced IP3R2 expression in Ank2 cKO astrocytes, approaches such as DREADD-mediated activation of Gq, one of the upstream regulators of IP3R2, would not be appropriate." I feel this argument might be too strong. The IP3R2 reduction is minimal in cKO mice as shown in Fig S6. While I am not insisting on performing DREADD-based experiments, I do think the authors should include a brief discussion on rescuing morphology using DREADD approach as a potential future research direction before the publication of the current form.

Reviewer #3

(Remarks to the Author)
I appreciate the authors’ efforts in addressing the points raised by us. Many points have now been addressed, but we feel that further additions to text, analysis, and additional experimental data are still required to support the claims of the manuscript. Most importantly, the feasibility of non-invasive blue light stimulation of the authors’ Opto-T1 construct needs to be convincingly demonstrated. Please find my responses and comments to the authors' responses in the attached DOCX as green text.

Version 2:

Reviewer comments:

Reviewer #3

(Remarks to the Author)
I thank the authors for the additional work they performed and presented. However, my concerns have not yet been fully addressed, as described below:

1. Fig. 7: Panel b seems to be missing the “cKO + Veh.” data? Panel c: As the authors state in their response, it is very surprising to find that BDNF application actually significantly reduces memory persistence. The authors should at least discuss this finding in their Discussion section and identify plausible hypotheses for this, based on literature. Otherwise, it may throw into doubt the mechanistic specificity of their cKO construct.
2. Fig. 5k-l: The authors claim that an ANOVA would be “statistically inappropriate” here if data from Fig. S7a,b were compared to the data presented here. Such a comparison has not been suggested. As I have pointed out, to make claims about “BDNF-dependent” effects, the inclusion of a vehicle control group would be necessary. The authors write that such claims have been toned down but, for example, the figure title still states “Ank2 interacts with IP3R2 and regulates astrocytic Ca2+ signaling and BDNF-dependent morphogenesis.”
3. Fig. S8: I appreciate the authors’ efforts to provide more evidence that their Opto-T1 construct works. The data provided are insufficient to gauge the efficacy of this construct though.
a. First, the data come from a single animal and show a single brain slice. I see no quantification presented, comparing left and right hemisphere. Why are the two ROIs from left and right hemisphere from different hippocampal layers (contralateral includes SP, ipsilateral does not)?
b. Secondly, I don’t see why the construct is expressed throughout the brain (as evidenced by HA staining) but GFAP seems mostly visible in hippocampus, but not the overlaying cortex that should receive stronger light levels.
c. What are the effective light levels inside the hippocampus with this method? This could be measured in a cadaver, for example. Rough estimates would be around 10-6, resulting in hippocampal irradiance of ~ 0.0005 µW/mm2.

d. Hong & Heo (2020) still used double the irradiance and three times the duration presented here.
e. Is vision becoming impaired from such high levels of constant blue light exposure? Can mice still perform visual discrimination, for example? Zhang et al. (2024, https://doi.org/10.1016/j.exer.2024.109946) describe significant retinal damage in mice when exposed for 2h to 2.55 mW/cm2 (0.0255 mW/mm2) 450 nm light. This is one twentieth of the light levels used in this study.

Version 4:

Reviewer comments:

Reviewer #3

(Remarks to the Author)
I have carefully evaluated the revised manuscript and the authors' responses to my comments. In this revision, the authors have satisfactorily addressed the last remaining concerns raised during the review process and have substantially strengthened the manuscript.
The study provides important insights into the role of astrocytic Ank2 in memory persistence and identifies a mechanistic link between Ank2-dependent astrocyte morphogenesis, BDNF-TrkB.T1-IP3R2 signaling, synaptic plasticity, and long-term memory maintenance. In addition, the development and application of the Opto-T1 tool represent a valuable technical advance for the field.
The revised manuscript is comprehensive and technically rigorous, and the conclusions are well supported by the presented data. I believe the work will be of broad interest to researchers studying astrocyte biology, memory mechanisms, and neuropsychiatric disorders.
Overall, I support publication of this manuscript in the journal.

Open Access This Peer Review File is licensed under a Creative Commons Attribution 4.0 International License, which permits use, sharing, adaptation, distribution and reproduction in any medium or format, as long as you give appropriate credit to the original author(s) and the source, provide a link to the Creative Commons license, and indicate if changes were made.
In cases where reviewers are anonymous, credit should be given to 'Anonymous Referee' and the source.
The images or other third party material in this Peer Review File are included in the article's Creative Commons license, unless indicated otherwise in a credit line to the material. If material is not included in the article's Creative Commons license and your intended use is not permitted by statutory regulation or exceeds the permitted use, you will need to obtain permission directly from the copyright holder.
To view a copy of this license, visit https://creativecommons.org/licenses/by/4.0/

Reviewer #1 (Remarks to the Author)

The manuscript provides significant new insights into the role of astrocytic Ankyrin-2 (Ank2) in memory persistence. The authors demonstrate that astrocyte-specific deletion of Ank2 impairs remote memory without affecting recent memory, reduces astrocyte morphological complexity, impairs long-term potentiation (LTP) maintenance, and reduces astrocytic participation in engram. Mechanistically, Ank2 interacts with IP3R2, regulates astrocyte morphogenesis, and memory persistence. Furthermore, selective activation of astrocytic BDNF signaling via optogenetics enhances remote memory, highlighting the astrocytic contribution to cognitive functions.

The work addresses an important and understudied aspect of astrocyte biology related to cognitive functions. The authors comprehensively use molecular genetics, electrophysiology, imaging techniques, and behavioral assays. They also applied innovative tools (e.g. astrocyte-eGRASP and Opto-cytTrkB.T1) that strengthen the impact of the study. This work provides clear demonstration of the necessity and sufficiency of astrocytic signaling in memory persistence.

Response: We greatly appreciate the reviewer's generous evaluation of our work and for highlighting both the novelty and the technical breadth of our study. We are encouraged by the recognition that our findings provide significant new insights into the astrocytic contribution to memory persistence, and that the diverse approaches we employed, including astrocyte-eGRASP and Opto-cytTrkB.T1, strengthen the impact of our conclusions. We have carefully considered all of the reviewer's constructive comments and addressed each point in detail. Our specific responses are outlined below in blue, with corresponding revisions to the manuscript indicated in red for clarity.

Comments

1. The rationale for selecting GFAP-creERT2 over Aldh1l1-creERT2 lacks robustness. GFAP is heterogeneously expressed in astrocytes and other cell types, which might confound specificity. ALDH1L1 is a more uniformly expressed astrocyte marker. The authors should provide a more substantial justification or clarify potential limitations explicitly in the manuscript.

Response: We thank the reviewer for this valuable comment. We selected GFAP-CreERT2 rather than Aldh1l1-CreERT2 because Aldh1l1 is abundantly expressed in peripheral organs such as the liver and lungs, whereas GFAP expression is largely restricted to the CNS. Since Ank2 is highly expressed in cardiomyocytes and other peripheral cell types, recombination outside the CNS could lead to severe systemic dysfunction, particularly as full-body Ank2 knockout is lethal (DOI: 10.1016/j.celrep.2023.112784). We acknowledge that GFAP-CreERT2 may introduce some heterogeneity, and to address this limitation we also employed a Cre virus containing the 4x6T cassette (DOI: 10.1038/s41467-023-42746-w) in Ank2 floxed mice to further delineate astrocyte-specific effects. In contrast, although Aldh1l1-CreERT2 may label a broader astrocyte population, its peripheral expression would introduce systemic confounds and complicate interpretation of CNS-specific phenotypes. We have now clarified these considerations in the revised manuscript.

Revised manuscript

Results [lines 61-64]

Given the broad expression of *Ank2* throughout the body, including in the heart, thymus, and other tissues[11,28], we used the GFAP-CreER^{T2} line to induce tamoxifen-dependent Cre expression predominantly in brain astrocytes[29], thereby reducing peripheral recombination compared to ALDH1l1-CreER^{T2} line[30].

Discussion [lines 470-474]

While GFAP-CreER^{T2} was selected in this study to reduce peripheral recombination, ALDH1l1-CreER^{T2} exhibits pan-astrocytic expression and targets a broader range of astrocyte subtypes, which could reveal additional roles of Ank2 in astrocyte populations not efficiently targeted by GFAP-based approaches. Notably, astrocyte-specific ARL13B deletion using ALDH1l1-CreER^{T2}, which disrupts primary cilia, leads to reduced Ank2 expression and a decrease in astrocytic surface area[60].

2. "Astrocyte-engram neuron contact" usage can be confusing. Both astrocytes and neurons can form an engram (https://doi.org/10.1038/s41586-024-08170-w; https://doi.org/10.1016/j.tins.2021.07.006). Consider explicitly mentioning "astrocytic component of an engram" to avoid ambiguity.

Response: We thank the reviewer for this valuable comment and for pointing out the potential ambiguity. We agree that the phrase “astrocyte-engram neuron contact” could be interpreted in several ways. Since our study focuses on the role of Ank2 rather than on engram astrocytes themselves (e.g., Fos-positive astrocytes), we consider “astrocytic contacts on engram neurons” to be the most accurate description of our results. We have revised the manuscript accordingly. We also appreciate the reviewer’s insightful suggestion regarding the “astrocytic component of an engram,” which we agree is highly relevant for future studies and will be adopted when appropriate in subsequent work.

3. "PAPs" is also confusing since this abbreviation can refer to both "Perisynaptic Astrocytic Processes" and "Peripheral Astrocytic Processes" (approx. 50% each). Specifically, the authors appear to mean astrocytic leaflets (fine, organelle-free protrusions), not thicker organelle-containing astrocytic branches, which can also be perisynaptic (https://doi.org/10.3389/fncel.2018.00248). It is recommended to clearly define and consistently use "leaflets" in the text (https://doi.org/10.1016/j.tcb.2023.09.006).

Response: We thank the reviewer for pointing out this potential ambiguity. We agree with the recommendation and have replaced “PAPs” with “astrocytic leaflets” throughout the revised manuscript for clarity and consistency.

4. Reduced ezrin expression and GFAP changes observed in Ank2 cKO mice conflict with previous literature showing inverse relationships under pathological conditions. Previous studies have shown that ezrin deficiency leads to upregulation of GFAP expression (https://doi.org/10.1002/glia.24253), and increased GFAP with reduced ezrin expression is correlated with astrocytic atrophy in aging and

conditions such as Alzheimer's disease and depression. Address this discrepancy and discuss potential underlying mechanisms.

Response: We appreciate the reviewer's careful observation regarding the apparent discrepancy between our results and previous reports. We acknowledge that our finding of reduced ezrin expression together with GFAP changes in Ank2 cKO astrocytes differs from studies showing an inverse relationship under certain pathological conditions (e.g., DOI: 10.1002/glia.24253). However, this correlation is not universal, as astrocyte-specific ezrin deletion has also been reported to show a trend toward decreased GFAP expression (Badia-Soteras et al., 2023, DOI: 10.1016/j.biopsych.2022.10.013). In addition, ezrin has been identified as a binding partner of Ank2 (Liu et al., 2018, DOI: 10.1038/s41467-018-03523-2), suggesting that Ank2 may influence ezrin localization or stability in astrocytes. While the precise mechanism remains to be clarified, these findings raise the possibility that Ank2 loss could uniquely alter ezrin-GFAP regulation compared to other contexts. We have revised the manuscript to explicitly discuss this discrepancy and its potential mechanistic basis.

Revised manuscript

Discussion [lines 441-447]

Interestingly, ezrin expression was reduced in Ank2 cKO astrocytes, which is unexpected because previous studies reported an inverse relationship between ezrin and GFAP in certain pathological conditions[56]. This correlation is not universal, as ezrin deletion in astrocytes has also been reported to show a trend toward decreased GFAP expression[6]. Ezrin has also been identified as a binding partner of Ank2 by affinity purification mass spectrometry[57], suggesting that Ank2 may influence ezrin localization or stability, although this remains untested in astrocytes. As a structural scaffold, Ank2 shapes astrocytic morphology and interacts with many other proteins, making it difficult to separate direct from indirect effects in this study.

5. The binding of Ank2 to IP3R2 and its role in astrocytic calcium signaling are primarily correlative. Direct functional experiments demonstrating Ank2's influence on IP3R2-mediated calcium dynamics are necessary to establish causality. Astrocytic atrophy and reduced communication with neurons may be the primary reasons for decreased calcium activity in astrocytes in Ank2 cKO mice. The authors should clearly state this limitation.

Response: We appreciate the reviewer's insightful comment. We agree that our initial evidence for Ank2-IP3R2 interaction was primarily correlative and that direct functional experiments are required to establish causality. To address this concern, we conducted additional analyses and experiments. First, STRING analysis of Ank2 molecular functions revealed the strongest enrichment for inositol 1,4,5-triphosphate-sensitive calcium-release channel activity, suggesting a functional link to IP3Rs. Second, to directly test whether the Ank2-IP3R2 interaction is required for astrocytic morphogenesis, we generated a dominant-negative construct containing the ankyrin-binding domain (ABD) of IP3R2 and expressed it in astrocytes. While this manipulation had little effect under basal conditions, it significantly impaired BDNF-dependent astrocyte morphogenesis, indicating that Ank2-IP3R2 binding is critical for astrocytic BDNF signaling.

At the same time, we acknowledge the reviewer's point that reduced astrocytic Ca^{2+} activity in Ank2 cKO mice could also reflect secondary consequences of structural atrophy and diminished astrocyte-neuron communication. Indeed, although we observed no changes in spontaneous EPSCs or IPSCs, it remains possible that altered distances between astrocytes and synapses limited the detection of functional differences.

In summary, our findings support a functional contribution of Ank2-IP3R2 interaction to BDNF-dependent astrocyte morphogenesis, while we also recognize that alternative explanations such as astrocytic shrinkage and altered neuron-astrocyte interactions could also contribute to the observed reduction in astrocytic Ca^{2+} activity. We have revised the Discussion to clearly state this limitation.

Revised manuscript

Result [lines 229-231]

STRING analysis of Ank2 molecular function revealed strong enrichment for inositol 1,4,5-triphosphate-sensitive calcium-release channel activity and high association scores with cytoskeletal proteins (Fig. 5a), suggesting a functional link to IP3Rs.

Result [lines 248-255]

To specifically interrogate the functional relevance of the Ank2-IP3R2 interaction, we targeted the ankyrin-binding domain (ABD) located in residues 955-991, which is conserved among IP3Rs[46]. We expressed the IP3R2 ABD as a dominant-negative construct (AAV-GFAP-eGFP-P2A-IP3R2 ABD) in astrocytes (Fig. 5i-l, Supplementary Fig. 7a, b). Expression of IP3R2 ABD alone did not alter GFAP intensity (Supplementary Fig. 7a, b). We next asked whether expression of IP3R2 ABD alters BDNF-dependent astrocyte morphogenesis *in vivo*. Hippocampal infusion of BDNF (100 ng/0.4 µL) induced astrocyte morphogenesis (Supplementary Fig. 7c-e). Astrocytes expressing IP3R2 ABD exhibited significantly lower GFAP volume after BDNF infusion compared with controls (Fig. 5k, l).

Discussion [lines 448-458]

It should be acknowledged that the present study focused primarily on the BDNF-IP3R2 pathway, yet other mechanisms could also contribute to the observed phenotypes. Exploring these alternative possibilities will be necessary to fully understand the roles of Ank2 in astrocytic Ca^{2+} signaling and neuron-astrocyte interactions. For example, the reduced Ca^{2+} activity in Ank2 cKO astrocytes may reflect not only impaired IP3R2-dependent signaling, as seen in IP3R2 knockout mice, but also consequences of astrocytic atrophy and reduced neuron-astrocyte communication. Downstream of BDNF-IP3R signaling, store-operated Ca^{2+} channels have been proposed as an additional source of $Ca^{2+,}$ which could further amplify the effects of BDNF. Interestingly, in cardiomyocytes, Ank2 has been reported to interact with Na^{+}-K^{+} ATPase and Na^{+}-Ca^{2+} exchanger[58], both of which are important Ca^{2+} sources in astrocytes[59]. The potential role of Ank2-interacting proteins in the ER and plasma membrane (PM) in assembling complexes[43] that may facilitate ER-PM junction formation and modulate Ca^{2+} signaling in astrocytes warrants further study.

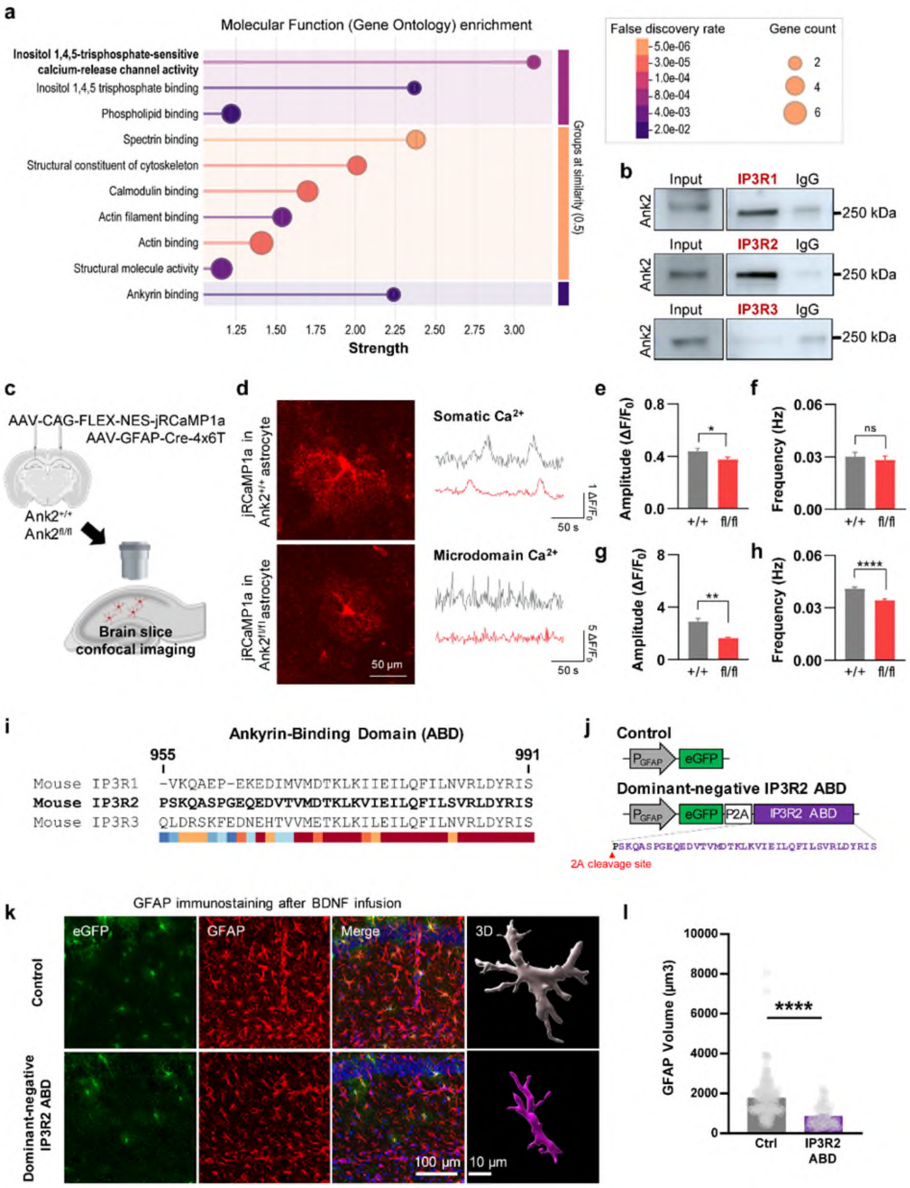

Fig. 5. Ank2 interacts with IP3R2 and regulates astrocytic Ca2+ signaling and BDNF-dependent morphogenesis. (a) STRING analysis of Ank2 molecular function enrichment showing associations with inositol 1,4,5-triphosphate-sensitive calcium-release channel activity and cytoskeletal proteins. (b) Co-immunoprecipitation (Co-IP) of Ank2 with IP3R1, IP3R2, and IP3R3 from mouse brain lysates. (c) Experimental scheme for the expression of jRCaMP1a (Ca^{2+} indicator) with Cre-dependent deletion of Ank2. (d, left) Representative images of jRCaMP1a-expressing hippocampal astrocytes from $Ank2^{+/+}$ (top) and $Ank2^{fl/fl}$ (bottom) mice. (d, right) Representative traces of jRCaMP1a fluorescence from soma (top) and microdomains (bottom) in $Ank2^{+/+}$ (gray) and $Ank2^{fl/fl}$ (red) astrocytes. (e-h) Quantification of $\Delta F/F_0$ jRCaMP1a signals in soma (e, f) and microdomains (g, h) of $Ank2^{+/+}$ and $Ank2^{fl/fl}$ astrocytes. Control group, n = 51 soma and n = 433 microdomains from 3 mice; $Ank2^{GFAP}$ cKO group, n = 40 soma and n = 289 microdomains from 2 mice. (i) Alignment of ankyrin-binding domains (ABD, residues 955-991) conserved across IP3R isoforms. (j) Schematic of AAV constructs for expression of control (GFAP-eGFP) and dominant-negative IP3R2 ABD (GFAP-eGFP-P2A-IP3R2 ABD). (k) Representative images of GFAP immunostaining and eGFP signals in hippocampal tissue following BDNF infusion (100 ng/0.4 µL). (l) Quantification of GFAP volume in astrocytes expressing control or IP3R2 ABD constructs after BDNF infusion. Control group, n = 167 astrocytes from 3 mice; IP3R2 ABD group, n = 75 astrocytes from 3 mice. Data are mean ± SEM. *P < 0.05, **P < 0.01, ****P < 0.0001; ns, not significant. Mann-Whitney test (e-h) and Welch's t-test (l). Detailed statistics are provided in Table S1.

6. Potential alternative explanations for impaired LTP (such as quicker deterioration of slices in Ank2 cKO mice due to reduced astrocytic metabolic support) should be considered and experimentally addressed or at least thoroughly discussed. This is important given that astrocytic shrinkage can impair metabolic support, potentially leading to faster tissue deterioration ex vivo.

Response: We appreciate the reviewer's important comment regarding potential metabolic stress due to astrocytic shrinkage. To examine whether faster tissue deterioration *ex vivo* could account for the impaired LTP, we tested whether Ank2 cKO slices showed gradual deterioration in the absence of stimulation. Specifically, we recorded baseline fEPSPs for up to 3.5 hours without applying TBS and did not observe any significant change over this period, suggesting that general slice viability is not the primary cause of impaired LTP.

Importantly, we acknowledge that astrocytic shrinkage can, in principle, compromise metabolic support to neurons, and this remains an important possibility. While our current data do not directly address this mechanism, we agree with the reviewer that further investigation will be required to determine whether impaired metabolic support contributes to the LTP deficits observed in Ank2 cKO mice. In particular, our astrocyte-eGRASP results revealed that Ank2 deletion reduces learning-dependent astrocytic contacts with engram neurons, raising the possibility that insufficient astrocytic interactions with these memory-related synapses may limit both structural stabilization and metabolic support. We have added this point to the Discussion.

Revised manuscript

Results [lines 193-194]

Baseline fEPSPs remained stable without TBS (Fig. 4a, unfilled red circle), thereby excluding slice deterioration as a contributing factor.

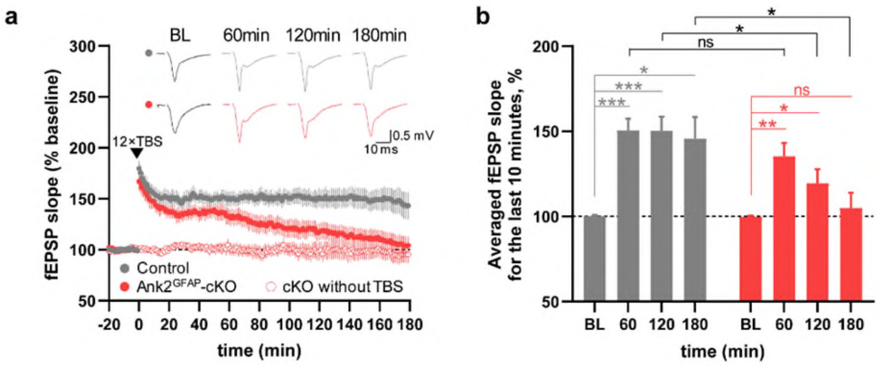

Fig. 1. Astrocytic Ank2 deletion impairs LTP maintenance and reduces learning-dependent astrocytic contacts with engram neurons in the hippocampus. (a) LTP induced by 12-theta burst stimulation (12×TBS). (b) Averaged fEPSP slope changes at 60, 120, and 180 min after 12×TBS. Control group, n = 8 slices from 3 mice; Ank2^{GFAP} cKO group, n = 8 slices from 3 mice; Ank2^{GFAP} cKO without TBS group, n = 11 slices from 2 mice.

Discussion [lines 459-468] Finally, while ex vivo recordings revealed impaired LTP maintenance in Ank2 cKO mice, this finding should be interpreted with caution. Although LTP has long been regarded as a cellular correlate of memory storage, actual learning and memory formation involve multiple levels of

consolidation, from synaptic to systems-level processes. Therefore, impairment of L-LTP over 3 hours cannot be directly equated to a 3-hour limit in memory retention. Nevertheless, the data indicate a reduced ability to maintain synaptic potentiation, and it is possible that diminished astrocytic metabolic support in Ank2 cKO mice limits the capacity to sustain potentiated synapses. Interestingly, we did not detect significant changes in the ending radius of GFAP-positive branches, which may suggest that a minimal level of metabolic support and astrocytic syncytium is still preserved. Given that astrocytic contacts on engram neurons may serve as hubs for such metabolic support to maintain enlarged spines, future work exploring this possibility could provide important mechanistic insight.

Reviewer #2

Overall Assessment:

This manuscript utilizes transgenic mice or Cre-virus mediated astrocyte-specific knockdown of Ank2, demonstrating that conditional knockout (cKO) mice exhibit impaired remote memory recall, reduced astrocytic morphology, decreased synaptic interactions leading to impaired synaptic plasticity and LTP. These effects are mediated through the BDNF-TrkB.T1-IP3R2 Ca2+ signaling pathway. The finding in this study about astrocytes Ank2 specifically in remote memory is interesting, adding valuable insights for the field. While the study presents a logically coherent narrative with reasonable experimental design (particularly the innovative Opto-T1 tool), several critical issues need to be addressed prior to publication.

Response: We sincerely appreciate the reviewer's thoughtful overall assessment of our manuscript. We are encouraged by the recognition that our work provides novel insights into the role of astrocytic Ank2 in remote memory. We have carefully considered all of the critical issues raised and revised the manuscript accordingly. Our specific responses to each point are outlined below in blue, with corresponding revisions to the manuscript indicated in red for clarity.

Major Concerns:

1. The authors employ GFAP-creERT2 mice for astrocyte-specific Ank2 knockout. While cited reference #32 reports high astrocyte specificity (>80%) but relative low astrocyte coverage (50-60%) in hippocampal CA1 and dentate gyrus with this line, other reviews note that GFAP-Cre/ERT2 exhibits low expression in astrocytes relative to other cell types (Yu X, Nagai J, Khakh BS. Improved tools to study astrocytes. Nat Rev Neurosci. 2020 Mar;21(3):121-138). Although WB shows ~50% Ank2 reduction, this was performed on whole hippocampus CA1 tissue lysates without astrocyte sorting. Additional validation of expression efficiency and specificity of Cre recombinase, and Ank2 knockdown efficiency in astrocytes is required, such as: immunofluorescence co-staining (providing spatial distribution data) or WB analysis of sorted astrocytes.

Response: We thank the reviewer for raising this important concern regarding astrocyte specificity and recombination efficiency. We selected GFAP-CreERT2 rather than Aldh1l1-CreERT2 because Aldh1l1 is abundantly expressed in peripheral tissues (e.g., liver, lung), which increases the risk of peripheral recombination, whereas GFAP expression is largely restricted to the CNS. In this study, we employed the Tg(GFAP-Cre/ERT2)13Kdmc line, which has been reported to exhibit strong astrocytic specificity (DOI: 10.5607/en.2018.27.6.508).

For cell-type specificity validation, we note that there is currently no antibody validated for detecting Ank2 in brain slices. For this reason, previous neuronal Ank2 studies also did not demonstrate cell-type-specific decrease, but instead relied on western blot analysis (Yoon et al., 2023, DOI: 10.1016/j.celrep.2023.112784, Oh et al., 2023, DOI: 10.1038/s41467-023-39203-z). Nevertheless, to further ensure the specific function of Ank2 in hippocampal astrocytes, we employed a Cre virus containing the 4x6T cassette (DOI: 10.1038/s41467-023-42746-w), which enhances astrocytic specificity by selectively suppressing neuronal expression. We additionally validated recombination specificity by quantifying Cre expression with Sox9, a reliable astrocytic nuclear marker, and found that nearly all Cre-mCherry signals co-localized with Sox9-positive nuclei. This confirms that Cre expression occurred specifically in astrocytes. Furthermore, when this astrocyte-specific Cre virus was injected into the hippocampus, we observed a reduction in memory persistence similar to that seen with GFAP-CreERT2-

mediated Ank2 deletion, thereby supporting the conclusion that Ank2 function in astrocytes is critical for memory persistence.

Revised manuscript

Results [lines 104-112]

To test whether the remote memory deficit in Ank2^{GFAP} cKO mice results specifically from the loss of astrocytic Ank2 in the hippocampus, we injected an adeno-associated virus (AAV) carrying Cre recombinase under the GFAP promoter (AAV-GfaABC1D-NLS-Cre-mCh-4x6T)[34] into the CA1 region of Ank2$^{fl/fl}$ mice. This construct contains a 4x6T miRNA targeting cassette that markedly enhances astrocyte specificity[34], which was reproduced (Supplementary Fig. 3a,b). As a control, we used an AAV expressing Flp recombinase (AAV-GfaABC1D-NLS-Flp-mCh-4x6T). Consistent with our earlier findings, mice with hippocampal astrocyte-specific Ank2 deletion displayed intact recent memory but impaired remote memory (Fig. 1j-n). These results demonstrate that Ank2 in hippocampal astrocytes is required for remote memory formation, highlighting its crucial role in memory persistence.

2. The co-localization rate of Cre-mCherry with GFAP in Fig 1J appears suboptimal. Quantitative analysis of mCherry/GFAP co-labeling efficiency and Ank2 knockdown efficiency in astrocytes should be provided to confirm targeting specificity.

Response: We thank the reviewer for this helpful suggestion. As shown in the original figure, Cre-mCherry is expressed as a fusion protein containing a nuclear localization signal (NLS), and therefore its signal is restricted to the nucleus. For this reason, we performed quantitative co-localization analysis with Sox9, a reliable nuclear marker for astrocytes. Cre-mCherry showed a high rate of co-localization with Sox9-positive nuclei, confirming that Cre expression was highly specific to hippocampal astrocytes. We have included these new analyses in Supplementary Figure 3a,b of the revised manuscript.

Revised manuscript

Supplementary Figure 3

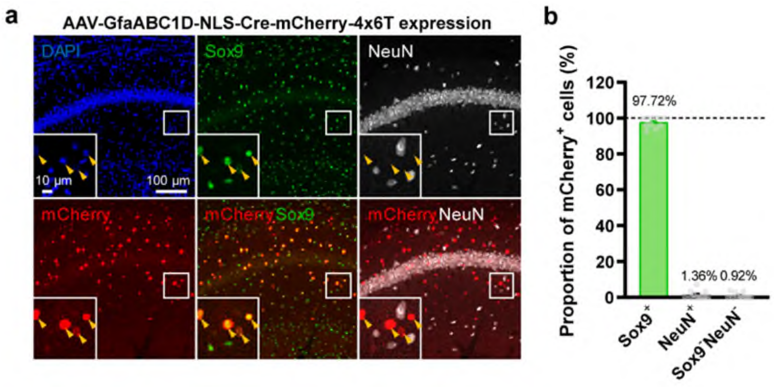

Supplementary Fig. 3. Specificity of AAV-GfaABC1D-NLS-Cre-mCherry-4x6T virus and quantification of Ank2 and Ezrin proteins as well as astrocyte number in the hippocampus. (a) Representative images of AAV-GfaABC1D-Cre-mCh-

4x6T expression. Nuclei are stained with DAPI (blue), Cre-mCherry (red), astrocytes with Sox9 (green), and neurons with NeuN (gray). Yellow arrowheads indicate mCherry localization in Sox9-positive ($Sox9^+$) cells. (b) Quantification of $mCherry^+$ cells from 11 images across 4 mice. Dots indicate the proportion of $mCherry^+$ cells (%) in each image. Of 630 $mCherry^+$ cells, 618 were $Sox9^+$, 6 were $NeuN^+$, and 6 were negative for both Sox9 and NeuN.

3. Figs 2I-2K analyze only 7-9 astrocytes - this sample size is statistically inadequate. While cell numbers can serve as n-values for morphological analysis, the number of animals used should also be reported.

Response: We thank the reviewer for raising this important concern regarding statistical adequacy. We agree that reporting only cell numbers can be misleading and that the number of animals should also be provided. In the revised manuscript, we have clearly indicated both the number of astrocytes analyzed and the number of animals from which they were obtained. These details have been added to the figure legend and Methods section to ensure transparency and statistical rigor.

Revised manuscript

Figure 2

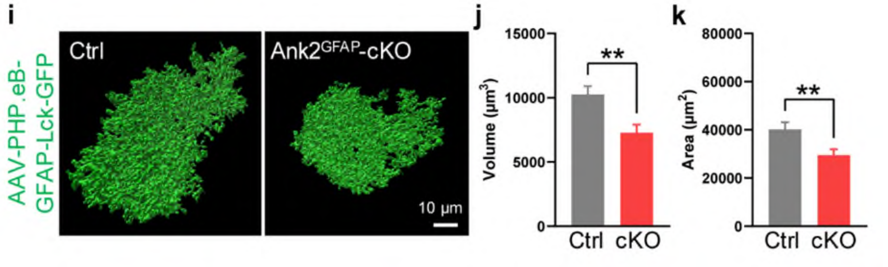

Fig. 1. Deletion of Ank2 in astrocytes reduces astrocyte volume and morphological complexity. (i) Visualization of whole astrocyte morphology with Lck-GFP expression in control and $Ank2^{GFAP}$ cKO mice. (j) Quantification of astrocyte volume. Control group, n = 20 astrocytes from 4 mice; $Ank2^{GFAP}$ cKO group, n = 19 astrocytes from 3 mice. (k) Surface area of hippocampal astrocytes. Control group, n = 20 astrocytes; $Ank2^{GFAP}$ cKO group, n = 19 astrocytes. Data are mean ± SEM. *P < 0.05, **P < 0.01, ***P < 0.001, ****P < 0.0001; ns, not significant. Mann-Whitney test (b, d-h), unpaired t-test (j, k). Detailed statistics are provided in Table S1.

4. The astrocyte-eGRASP signal (Fig 4) infers synaptic coverage changes, and the Graphical summary (Supplementary Fig. 7) suggests reduced astrocytic process envelopment of synapses. However, Electron microscopy demonstrating tripartite synapse structural changes or advanced imaging tools (such as NAPA, Octeau JC, Chai H, Jiang R, Bonanno SL, Martin KC, Khakh BS. An Optical Neuron-Astrocyte Proximity Assay at Synaptic Distance Scales. Neuron. 2018 Apr 4;98(1):49-66.e9.) is recommended to claim this issue more sufficiently.

Response: We thank the reviewer for this valuable suggestion. We fully agree that tools such as NAPA provide a powerful way to assess neuron-astrocyte proximity using FRET-based imaging. However, NAPA requires specialized FRET setups and complex construct validation, which were not feasible within the revision period. By contrast, astrocyte-eGRASP is a recently developed tool that can be readily quantified with conventional confocal microscopy. We therefore used astrocyte-eGRASP to demonstrate that Ank2 deletion reduces astrocytic contacts on engram neurons.

In line with the reviewer's recommendation, we also attempted electron microscopy as a complementary approach. Although this method does not allow us to trace learning-dependent contacts with engram neurons, it did provide an interesting result. Ank2 cKO mice exhibited a modest but significant increase in excitatory synapse density compared to controls under baseline condition (i.e., before learning). This observation may reflect a compensatory adjustment to maintain basal synaptic transmission despite reduced astrocytic volume. While these TEM data are not central to our main conclusion, they offered unexpected insights, and we are grateful to the reviewer for motivating us to perform this additional analysis. We have incorporated these TEM results into the revised manuscript.

Revised manuscript

Figure 4

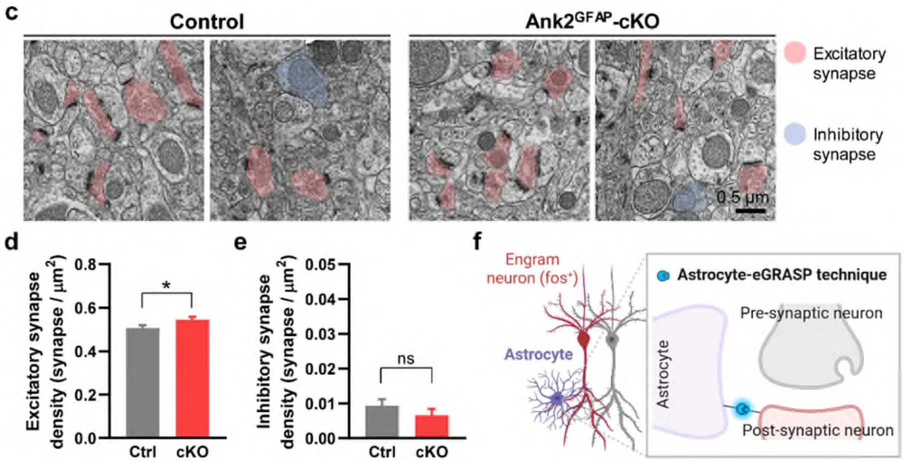

Fig. 2. (c) Representative TEM images showing excitatory and inhibitory synapses in hippocampal CA1. (d, e) Quantification of excitatory (d) and inhibitory (e) synapse density. Control group, n = 90 images from 3 mice; Ank2^{GFAP} cKO group, n = 90 images from 3 mice.

Results [lines 196-203]

Astrocytic leaflets enwrap active synapses, supporting their stabilization and maintenance[8], which may contribute to remote memory consolidation. We wondered whether astrocytic Ank2 loss alters astrocytic contacts on engram neurons after memory acquisition[9,10]. First, using transmission electron microscopy (TEM), we quantified the density of excitatory and inhibitory synapses in hippocampal CA1. Inhibitory synapse density was unchanged, whereas excitatory synapse density was modestly but significantly increased in Ank2^{GFAP} cKO mice compared to controls (Fig. 4c-e). Given that basal synaptic transmission was unaltered in Ank2^{GFAP} cKO slices, this increase likely reflects a compensatory adjustment to preserve synaptic transmission despite reduced astrocytic volume and complexity.

5. The proposed mechanism involves Ank2 knockdown → impaired IP3R2 function → reduced Ca2+ signaling → morphological/functional deficits. Does IP3R2 activation or astrocytic Ca2+ elevation rescue astrocyte morphology, tripartite synapse structure, synaptic plasticity or behavioral deficits in cKO mice?

Response: We thank the reviewer for this insightful and important question. We agree that directly testing whether IP3R2 activation or astrocytic Ca^{2+} elevation can rescue morphology, tripartite synapse structure, or behavioral deficits would provide strong mechanistic evidence. However, we also note that this is technically and conceptually challenging. While lowering astrocytic Ca^{2+} levels can be achieved relatively easily with tools such as CalEx, elevating Ca^{2+} in a physiologically meaningful manner is far more difficult. Extracellular versus intracellular Ca^{2+} sources can have distinct effects, and even ER-derived Ca^{2+} signals can differ depending on the release mechanism (DOI: 10.1523/JNEUROSCI.1717-08.2008). Accordingly, while we agree such rescue experiments would be highly informative, designing them in a selective and interpretable manner remains technically difficult at present.

In addition, because we observed reduced IP3R2 expression in Ank2 cKO astrocytes, approaches such as DREADD-mediated activation of Gq, one of the upstream regulators of IP3R2, would not be appropriate. As an alternative, we took advantage of Opto-T1 to activate TrkB.T1 signaling, which allowed us to provide an upstream sufficiency test in naïve mice. Through this approach, we demonstrated that enhancing BDNF-TrkB.T1 signaling can restore astrocytic morphology and memory persistence, thereby supporting the mechanistic link while avoiding the ambiguities inherent to direct Ca^{2+} rescue experiments.

6. Since BDNF infusion and Opto-T1 activation modify astrocyte morphology and memory persistence, comparative data such as astrocytic Ca2+ signaling, LTP, astrocyte-neuron contacts under both BDNF and Opto-T1 conditions between cKO and ctrl should be provided to strengthen the mechanistic chain.

Response: We thank the reviewer for this insightful comment. To directly examine the role of BDNF/Opto-T1 activation in sustaining synaptic plasticity, we performed LTP experiments using a 3xTBS protocol, which induces only E-LTP that normally decays after about one hour (DOI: 10.1038/npjscilearn.2016.3). When 50 µg/ml BDNF was applied for 10 minutes after one hour following 3xTBS, the decaying LTP was rescued and maintained, indicating that BDNF infusion can sustain long-term potentiation. Similarly, in hippocampal slices expressing Opto-T1, 10 minutes of blue light stimulation delivered one hour after 3xTBS also preserved LTP, demonstrating that astrocytic Opto-T1 activation is sufficient to support long-term synaptic plasticity. We acknowledge that the downstream signaling pathways of astrocytic TrkB.T1 remain incompletely established, and we plan to further investigate the precise mechanisms in future work.

Revised manuscript

Results [lines 360-368]

We next asked whether activation of astrocytic TrkB.T1 signaling by Opto-T1 could reproduce the well-established contribution of BDNF to maintaining L-LTP[50]. Unlike 12×TBS (Fig. 4a), 3×TBS in naïve hippocampal slices induced only E-LTP (Fig. 8g, h), consistent with the previous report[50]. Application of BDNF (50 ng/ml, 10 min) 1 h after 3×TBS allowed the maintenance of LTP for at least 3 h. To test whether astrocytic TrkB.T1 activation could reproduce this effect, we performed LTP recordings in mice injected in hippocampal CA1 with AAV-GFAP-Opto-T1-HA (co-injected with AAV-EF1a-mCherry to verify the injection site). In hippocampal slices from Opto-T1-expressing mice, continuous blue light

stimulation (50 μW/mm^2, 10 min, 1 h post-3×TBS) likewise maintained LTP for at least 3 h (Fig. 8g, h), indicating that astrocytic TrkB.T1 activation is sufficient to sustain LTP maintenance.

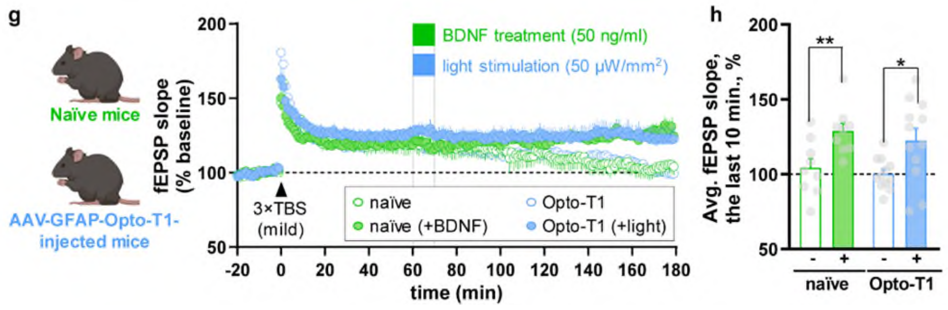

Fig. 8. (g) fEPSP slope changes by three theta-burst stimulations (3×TBS) with or without BDNF (50 ng/ml, 10 min) or Opto-T1 activation (50 μW/mm^2, continuous for 10 min). naïve, n = 9 slices from 5 mice; naïve+BDNF, n = 9 from 5 mice; Opto-T1, n = 12 slices from 5 mice; Opto-T1+light, n = 12 slices from 5 mice. (h) Summary graph of late-phase LTP (L-LTP).

7. Supplementary Fig. 4d/g exhibits an unstable baseline (notably for IPSCs), and a discrepancy exists between trace scale bars and histogram frequencies (Supplementary Fig. 4f/i): the ~5s trace in panel d displays ~10 EPSCs, inconsistent with the reported ~4Hz mean frequency.

Response: We thank the reviewer for carefully pointing out the issues in our supplementary data, particularly regarding the unstable baseline for IPSCs and the apparent discrepancy between the representative trace and mean frequencies for EPSCs.

First, regarding the EPSC frequency discrepancy (Supplementary Fig. 4d/f), the originally presented trace showed relatively small peaks that may not have been clearly distinguishable as representative EPSC events. In fact, the mean frequency is approximately 3.3–3.4 Hz, typically yielding 15-17 peaks over the 5-second recording window. To better reflect this, we have replaced the trace with one that more clearly represents the mean frequency and displays identifiable EPSC events.

Second, for the IPSC traces in Supplementary Fig. 4g/i, we agree that the baseline appeared unstable. This is due to our recording configuration, in which IPSCs were measured at a holding potential of 0 mV without the application of glutamate receptor blockers (e.g., CNQX or AP5), potentially resulting in increased background noise and a less stable-looking trace. However, the recordings themselves were stable. To better illustrate this, we extended the length of the representative trace to 10-second. The baseline current itself remains steady, even though IPSC events vary in size. Most importantly, both control and Ank2 cKO conditions were recorded under same settings and analyzed in a blinded manner. Therefore, we believe the data provide sufficient basis to conclude that IPSCs in CA1 pyramidal neurons do not differ significantly between control and Ank2 cKO mice.

Revised manuscript

Supplementary Figure 4

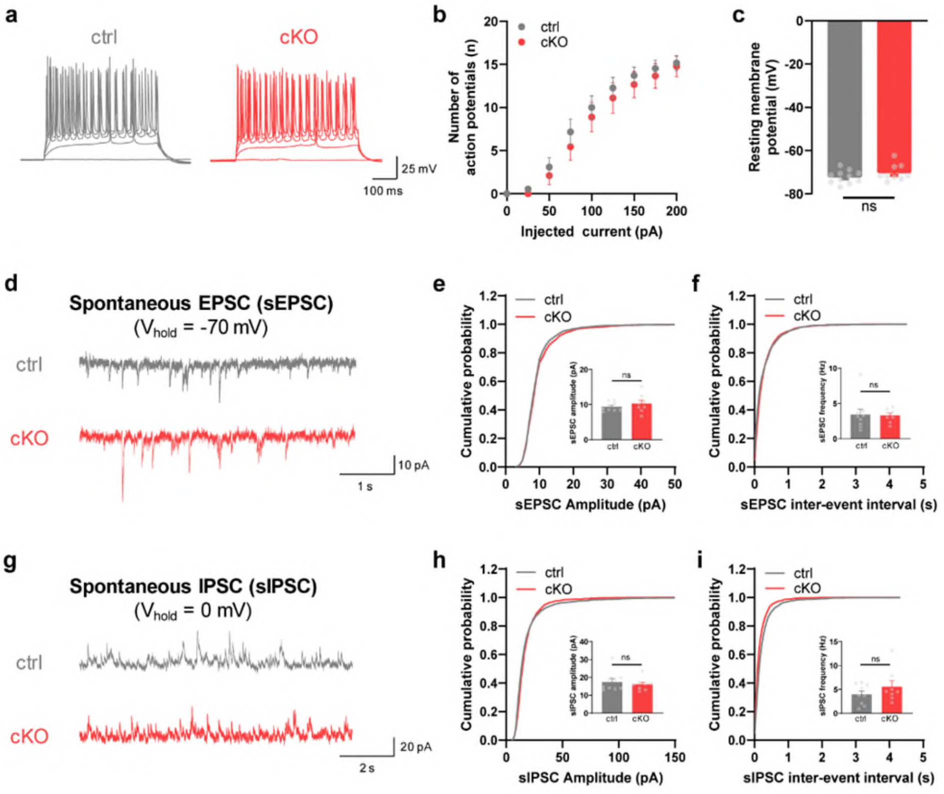

a
ctrl
cKO
25 mV
100 ms
b
ctrl
cKO
Number of action potentials (n)
Injected current (pA)
c
Resting membrane potential (mV)
ns
d
Spontaneous EPSC (sEPSC)
(V hold = -70 mV)
ctrl
cKO
10 pA
1 s
e
Cumulative probability
sEPSC Amplitude (pA)
sEPSC amplitude (pA)
ns
f
Cumulative probability
sEPSC inter-event interval (s)
sEPSC frequency (Hz)
ns
g
Spontaneous IPSC (sIPSC)
(V hold = 0 mV)
ctrl
cKO
20 pA
2 s
h
Cumulative probability
sIPSC Amplitude (pA)
sIPSC amplitude (pA)
ns
i
Cumulative probability
sIPSC inter-event interval (s)
sIPSC frequency (Hz)
ns

8. The high background in Fig 8C HA staining compromises astrocyte morphology visualization, necessitating higher magnification images and co-localization analysis (HA/astrocyte/neuron markers) to confirm viral infection efficiency and targeting specificity.

Response: We thank the reviewer for raising this point regarding the HA staining. We believe the HA signals observed in Fig. 8C represent true positive labeling rather than nonspecific background, as they display a round, bushy morphology consistent with our other whole-astrocyte staining (e.g., Fig. 2f, including fine processes) and distinct from the neuronal morphology in CA1. To further strengthen this point, we acquired higher-magnification confocal images and performed quantitative analysis of viral infection efficiency and targeting specificity. These new data have been incorporated into the revised manuscript.

Revised manuscript

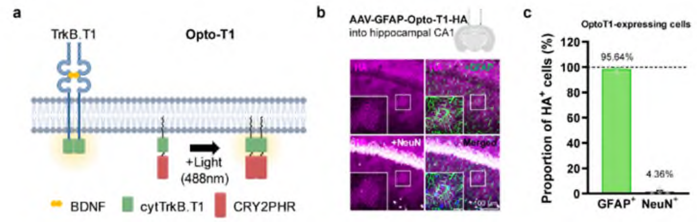

Fig. 8. Astrocyte-specific BDNF signaling sufficiently induces memory persistence. (a) Design of Opto-cytTrkB.T1 (Opto-T1), containing a light-sensitive CRY2PHR^{E281A} domain fused to the cytosolic domain of the TrkB.T1 receptor. (b) Representative image showing astrocyte-specific expression of AAV-GFAP-Opto-T1-HA. (c) Quantification of astrocytic specificity of Opto-T1 expression. Each dot indicates one slice. A total of 311 HA^{+} cells were analyzed from 4 slices across 3 mice.

9. Sholl analysis revealed a reduction in total branch length but no significant change in ending radius, warranting further discussion on its potential impact on synaptic coverage.

Response: We thank the reviewer for this helpful comment. It is important to note that our Sholl analysis was based on GFAP staining, which primarily labels thick main branches rather than fine astrocytic leaflets. In this context, the ending radius reflects the length of the longest GFAP-positive branch, whereas the total branch length represents the cumulative length of all such branches. In our data, the ending radius of main branches was unchanged, suggesting that the maximal projection range of GFAP-positive processes was preserved. However, the number of branches was reduced, leading to a shorter total branch length. This reduction in branching complexity could decrease the overall capacity for synaptic coverage, even though the projection range and a minimal level of astrocytic syncytium appear to be maintained. We have clarified this point in the revised discussion.

Revised manuscript [lines 465-467]

Interestingly, we did not detect significant changes in the ending radius of GFAP-positive branches, which may suggest that a minimal level of metabolic support and astrocytic syncytium is still preserved.

Minor Concerns:

1. Fig 1j schematic: Remove space in "2 w eeks" → "2 weeks".

2. Supplementary Figure 1 shows inconsistent font/axis formatting among panels a-d and others. Remove '**' from legends where absent in panels (apply to all legends).

3. Fig 7e: The Y-axis scale obscures data resolution. Please consider reducing the range.

4. Line 562: Add negative sign to "-70mV".

5. The panel labeling is inconsistent across figures, with lowercase letters (a, b, c, d) used in Figures 1 and 2, while uppercase letters (A, B, C, D) appear in Figure 3.

6. The P-values in Figure 2's figure caption are italicized, while those in other figure captions are not. Please standardize the formatting of all P-values across figures to comply with journal guidelines.

7. In Fig.5, “n=433” should be “n = 433”.

8. The term “GFAP-creERT2” should be uniformly corrected to “GFAP-CreERT2” (title case) throughout the manuscript.

Response: We appreciate the reviewer’s careful attention to these details, which has helped us improve the clarity and professionalism of the manuscript. We have corrected the typographical errors, standardized figure formatting (axis labels, legends, and P-value style), adjusted panel labeling for consistency, and unified terminology (e.g., GFAP-CreERT2) throughout the revised manuscript.

Reviewer #3 (Remarks to the Author):

While contextual fear memories are known to depend on neurons in the hippocampal formation, much less is known about the role of astrocytes in the formation and maintenance of memories. Hayoung et al. have used a wide range of experimental methods to demonstrate that the cytoskeletal adapter protein Ank2 supports BDNF-mediated structural plasticity in astrocytes that is required and sufficient for remote memory expression.

While their work is technically of a high standard, encompasses a wide range of methods, and convincingly demonstrates links between Ank2 and the structural integrity of astrocytes, the links between Ank2 and BDNF as well as Ank2 and memory persistence currently appear weak and require further evidence to support the authors' claims.

If further evidence can be provided, the identification of a mechanistic link between cytoskeletal proteins in astrocytes supporting memory persistence in the hippocampus would be an important discovery to warrant publication in Nature Communications.

Response: We greatly appreciate the reviewer's thoughtful assessment of our study. We are encouraged by the recognition that our work is technically rigorous and that it highlights an important and underexplored role of astrocytic Ank2 in memory. At the same time, we acknowledge the reviewer's concerns regarding the strength of the evidence linking Ank2-IP3R2 in BDNF signaling and to memory persistence. We have carefully addressed these points through additional analyses and clarifications, which are detailed below in blue, with corresponding revisions to the manuscript indicated in red for clarity.

Major:

1. The authors' main argument rests on their finding of impaired remote memory performance after conditional Ank2 deletion in CA1 astrocytes (Fig. 1m). Where is Fig. 1n on memory persistence that is referred to in the legend? Is persistence affected? If not, why not? Were these injections performed bilaterally or unilaterally? – the figure legend/methods remain unclear on this. Why were only 9/8 mice tested here? The statistically differences are significant but barely so. They are definitively much weaker than those shown in Fig. 1f, which begs the question if there are other astrocytic populations that play a more important role? The authors should strengthen their claims by either consolidating their results with additional animals or identify other targets outside CA1 that could explain the clear behavioural phenotype seen in Fig. 1f.

Response: We thank the reviewer for this valuable comment and for pointing out the issues in Fig. 1. In the revised manuscript, we have clarified that Fig. 1n represents memory persistence following bilateral injections of Cre virus into the CA1 of Ank2 floxed mice. The figure legend and Methods section have been corrected to clearly describe the injection strategy. We increased the number of animals included in this analysis and obtained consistent results, which strengthen and support our original conclusion.

We also acknowledge that the behavioral effect size observed in CA1-targeted deletion is smaller than that seen with broader astrocytic manipulations, suggesting that additional astrocytic populations outside CA1 may contribute to memory persistence. The most likely candidates are astrocytes in regions such as the medial prefrontal cortex (mPFC) or anterior cingulate cortex (ACC), which have been highly implicated in the storage of remote memories. While a systematic dissection of these regions is beyond

the scope of the present study, we agree this represents an important avenue for future investigation, and we have noted this possibility in the revised Discussion.

Revised manuscript

Results

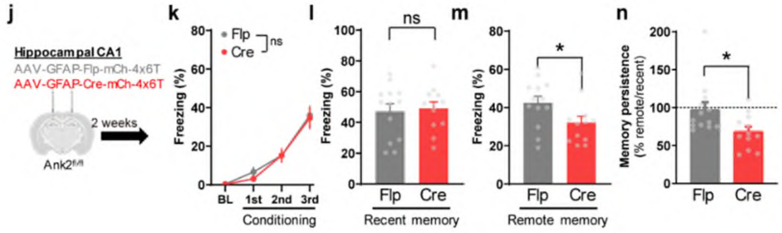

Fig. 1. Astrocytic Ank2 deletion impairs memory persistence. (j) Hippocampal astrocyte-specific Ank2 deletion. (k-n) Freezing behavior during acquisition (k), recent memory test (l), and remote memory test (m). (n) Memory persistence in mice with hippocampal astrocytic Ank2 deletion. Flp group, n = 13 mice; Cre group, n = 12 mice. Data are mean ± SEM. *$P < 0.05$, ***$P < 0.001$, ****$P < 0.0001$; ns, not significant. Two-way repeated measures ANOVA (d, h, k), unpaired t-test (e, f, l, m), Mann-Whitney test (g, n), and t-tests for (i): paired within groups, unpaired between groups. Detailed statistics are provided in Table S1.

Discussion [lines 469-479]

Beyond its role in memory persistence, Ank2 may have broader functions in astrocyte biology, including the regulation of astrocyte maturation. While GFAP-CreER^{T2} was selected in this study to reduce peripheral recombination, ALDH1l1-CreER^{T2} exhibits pan-astrocytic expression and targets a broader range of astrocyte subtypes, which could reveal additional roles of Ank2 in astrocyte populations not efficiently targeted by GFAP-based approaches. Notably, astrocyte-specific ARL13B deletion using ALDH1l1-CreER^{T2}, which disrupts primary cilia, leads to reduced Ank2 expression and a decrease in astrocytic surface area[60]. Given the link between astrocytic structural integrity and neurodevelopmental disorders such as ASD, further investigation is warranted to determine how Ank2 contributes to astrocyte maturation and whether it influences brain development. Whether Ank2 primarily regulates astrocytic morphology and signaling or also contributes to broader neurodevelopmental processes remains an open question, with potential implications for ASD and intellectual disability.

2. The relationship of BDNF with Ank2 appears rather weak and requires further data. For example, while the authors suggest an impaired “volume change (Δ Volume)” in cKO animals after BDNF treatment (Fig. 6d-e), it is unclear why the authors would quantify absolute differences and not proportional differences. Indeed, comparing Fig. 6c and 6d, it appears that booth ctrl and cKO volumes approximately double in volume after BDNF treatment, which suggests that cKO astrocytes are still responding the same way to BDNF as controls, despite their lower baseline. Furthermore, in Fig. 7, the authors claim that “BDNF-induced memory persistence is reduced in astrocyte-specific Ank2-deleted mice”, but the authors only compare control+vehicle, control+BDNF, and cKO+BDNF, but not the

criticial cKO+vehicle. Hence, it remains unclear what the baseline cKO level is that any BDNF effects would need to be compared to.

Response: We thank the reviewer for this insightful comment. We agree that both control and Ank2 cKO astrocytes show approximately a two-fold increase in volume following BDNF treatment. However, we chose to quantify absolute volume rather than proportional change because the ability of astrocytes to contact and support surrounding synapses is ultimately constrained by their absolute size. Thus, although the relative increase appeared similar, Ank2 cKO astrocytes began from a lower baseline and remained significantly smaller even after BDNF treatment, which likely limits their functional capacity to support synaptic interactions.

Regarding the cKO+vehicle condition, our behavioral experiments were specifically designed to test whether exogenous BDNF supports memory persistence and whether this effect requires astrocytic Ank2. Mild contextual fear conditioning does not induce remote memory persistence, as previously reported (DOI: 10.1007/s12035-018-1463-4). Consistent with this, and given that we had already observed that Ank2 cKO mice fail to form remote memory persistence even under stronger training conditions, we did not include a separate cKO+vehicle group, as this condition was not expected to yield remote memory either. Instead, we focused on comparing the effect of BDNF infusion across genotypes. First, in control mice, hippocampal BDNF infusion delivered 12 hours after training significantly enhanced memory persistence, indicating that exogenous BDNF is sufficient to induce memory persistence. Next, we asked whether this BDNF-dependent effect is preserved in Ank2 cKO mice. We found that, despite receiving identical BDNF treatment (with genotype being the only difference), Ank2 cKO mice exhibited markedly reduced memory persistence. This divergence indicates that astrocytic Ank2 is required for the BDNF-dependent memory persistence.

3. I remain unconvinced of the efficacy of the Opto-T1 construct. Since the authors newly developed this construct and show only a single quantitative panel featuring an unknown number of mice, further (in vitro) data is required to establish this tool as functional. I am furthermore surprised to see this construct improving memory persistence. Why is the memory persistence of controls in Fig. 1g >90%, while it is ~60% in Fig. 8i? Is this due to different shock protocols? If yes, the authors should also show the data with congruent shock protocols (3 shocks in Fig. 8; 1 shock in Fig. 1). Furthermore, to demonstrate the currently missing link to Ank2, the authors should aim to rescue the memory phenotype produced by Ank2-GFAP-cKO with the activation of opto-T1 in the hippocampus.

Response: We thank the reviewer for raising these important points. To strengthen the validity of our Opto-T1 construct, we performed additional experiments showing that its activation produces effects similar to BDNF treatment in sustaining long-term potentiation in the hippocampus, supporting the conclusion that Opto-T1 in astrocytes is functional. We also note that, since the seminal pioneering work of Rose et al. (2003, Nature), relatively little progress has been made in delineating the downstream signaling pathways of TrkB.T1 in astrocytes. We therefore view Opto-T1 as a useful new tool to pursue such mechanistic questions in future studies, and we are planning more detailed investigations of this tool and the downstream mechanisms of TrkB.T1.

In the present study, we included the Opto-T1 experiments for a specific reason. The Ank2 coding sequence is exceptionally large (~6 kbp), which made it technically unfeasible to restore Ank2 expression in an astrocyte-specific manner using our AAV virus system. As an alternative strategy to assess astrocytic sufficiency, we modified the well-established Opto-cytTrkB construct (Woo et al., 2019,

Cell Chemical Biology, Hong and Heo, 2020, Journal of Molecular Biology) by replacing the full-length TrkB domain with TrkB.T1. Aside from this domain difference, the construct is identical to the original tool, and indeed it mimicked BDNF-dependent astrocyte morphogenesis. This approach allowed us to test directly whether selective activation of astrocytic signaling is sufficient to support memory persistence, thereby complementing our loss-of-function experiments and underscoring the contribution of astrocytes.

A notable aspect of this tool is that it also increased GFAP expression, yet without inducing the detrimental features of reactive astrocytes that are typically associated with impaired LTP and memory. Instead, similar to BDNF treatment, astrocytic activation of Opto-T1 enhanced LTP maintenance and remote memory persistence, indicating that GFAP elevation in this context reflects functional astrocytic engagement rather than pathological reactivity. We believe this unique feature adds further significance to the Opto-T1 findings in the context of astrocytic roles in memory persistence.

Regarding the difference in memory persistence levels between Fig. 1 and Fig. 8, this was intentional. We used a weaker conditioning paradigm that results in minimal induction of BDNF expression around 12 hours after training and lower memory persistence, making this time window particularly suitable for testing whether exogenous BDNF (Fig. 7) or Opto-T1 activation (Fig. 8) can enhance memory persistence. Based on the existing literature and previous studies, and with approval from IACUC, we designed the experiments to specifically address three questions: (1) whether Ank2 is required to maintain memory persistence under strong conditioning, (2) whether exogenous BDNF can sustain memory persistence under mild conditioning where it normally fails, and (3) whether Ank2 is necessary for BDNF-induced memory persistence. Therefore, we focused experiments (Fig 7 and 8) on conditions where endogenous BDNF induction is minimal, rather than on strong conditioning in which BDNF is already robustly induced.

Finally, we sincerely thank the reviewer for suggesting the possibility of a rescue experiment in Ank2 cKO mice using Opto-T1, which we found truly inspiring. However, because the expression and functions of IP3R2 are already reduced in Ank2 cKO astrocytes, activating upstream signaling through Opto-T1 would likely be ineffective in resolving the deficits observed. However, the reviewer's comment has inspired us to consider exploring IP3R2 downstream signaling and to investigate whether these pathways can be modulated by optogenetic approaches. Such experiments, particularly those that drive astrocytic morphogenesis, would represent a powerful and informative direction, and we are very grateful for this suggestion. Given the scope of the current study, we felt it most appropriate to conclude our experiments here, while highlighting this as an exciting avenue for future work.

4. The authors need to explain the link between the visible decline of Ank2GFAP-cKO in L-LTP starting at 2 hours after LTP induction (Fig. 4b) and their behavioural results indicating no memory deficits 24 hours after conditioning (Fig. 1e). How do the authors reconcile the fact that LTP seems impaired already after 2 hours, while behavioural phenotypes remain intact for at least 24 hours? The authors need to discuss this point or produce further evidence to reconcile these results.

Response: We thank the reviewer for this important comment. We acknowledge that our *ex vivo* LTP recordings and the behavioral results occur on different timescales. While LTP has long been considered a cellular correlate of memory storage, actual learning and memory formation involve multiple layers of consolidation, including synaptic, circuit, and systems-level processes, which take hours to days. Thus, the impairment in L-LTP maintenance observed within a 3-hour recording window should not be directly equated to a strict time limit for memory retention *in vivo*, particularly in the context of *ex vivo* slices.

Although we confirmed in additional experiments that hippocampal slices can remain healthy for more than 3 hours (Fig. 4), *in vivo* conditions involve systemic consolidation processes across multiple brain regions and the action of various neuromodulators, which are not fully captured in slice preparations. Therefore, the LTP impairment we observed likely reflects only part of the overall process, which may explain the apparent temporal discrepancy. We have clarified this point in the revised manuscript.

Revised manuscript

Discussion [lines 459-465]

Finally, while ex vivo recordings revealed impaired LTP maintenance in Ank2 cKO mice, this finding should be interpreted with caution. Although LTP has long been regarded as a cellular correlate of memory storage, actual learning and memory formation involve multiple levels of consolidation, from synaptic to systems-level processes. Therefore, impairment of L-LTP over 3 hours cannot be directly equated to a 3-hour limit in memory retention. Nevertheless, the data indicate a reduced ability to maintain synaptic potentiation, and it is possible that diminished astrocytic metabolic support in Ank2 cKO mice limits the capacity to sustain potentiated synapses.

5. Why was eGRASP used in mice that underwent conditioning and memory tests if no behavioural results are shown (Fig. 4c-g)? The authors should show if memory expression levels are correlated with eGRASP density, as would be expected. If not, the authors need to explain this or adjust their model.

Response: We thank the reviewer for this thoughtful comment. As clearly described in the figure legend, main text, and Methods, the memory test was performed one day after training, and brains were immediately fixed thereafter for astrocyte-eGRASP analysis. We have now included the corresponding behavioral results below.

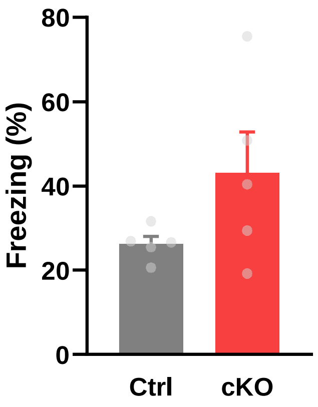

Although this experiment required additional manipulations (habituation to the anesthesia chamber, inhalation anesthesia, and doxycycline injection prior to learning), the results were consistent with those shown in Fig. 1. Ank2 cKO mice did not exhibit a deficit in recent memory. The cKO group even showed slightly higher freezing, which was largely driven by one mouse with high freezing levels. Importantly, despite this behavioral outcome, astrocyte-eGRASP density was clearly reduced in Ank2 cKO mice,

indicating that synaptic contact reduction can be detected independently of behavioral performance at recent time points.

We couldn't test the astrocyte-eGRASP in the remote timepoint because astrocyte-eGRASP is a novel tool (Kim et al., 2023), and its long-term stability has not yet been fully established. In particular, because our design used a doxycycline-based labeling strategy, both the stability of eGRASP reconstitution and the persistence of engram neuron labeling decline over time. For this reason, we focused our analysis on Day 1 after conditioning, when labeling is still reliable.

Importantly, this limitation also led to an unexpected finding. Even though behavioral recall on Day 1 did not differ between groups (Fig. 1 and above), astrocyte-eGRASP revealed a clear reduction in contacts onto engram neurons in Ank2 cKO mice at this recent time point. This indicates that astrocytic contacts are already altered one day after learning, before behavioral differences emerge (i.e., remote time point). Notably, this occurred on the day after the critical 12-hour window in which BDNF signaling is required for memory persistence, highlighting the significance of this observation. Further studies, for example using longitudinal *in vivo* imaging of astrocyte morphology and astrocytic contacts on engram neurons, will be needed to determine how astrocytic structure dynamically changes after learning (recent versus remote time points). Our results suggest that these early structural alterations may provide a mechanistic basis for later differences in spine stability and memory persistence.

6. The authors claim "Ank2 as a key regulator of IP3R2 function" (l. 269-270), referring only to one panel of co-immunoprecipitation (Fig. 5a) and a single article published in 2022 in Molecular Psychiatry. The data in Fig. 5b-h shows an impairment in calcium dynamics that could be the result of other cellular processes. The authors should either tone down this statement or find further evidence for this.

Response: We thank the reviewer for this valuable comment. We acknowledge that our initial wording "Ank2 as a key regulator of IP3R2 function" was overstated given the limited evidence originally presented. To strengthen this point, we performed additional analyses and experiments. First, STRING analysis of Ank2 molecular functions revealed the strongest enrichment for inositol 1,4,5-triphosphate-sensitive calcium-release channel activity, suggesting a functional link to IP3Rs. Second, to directly test whether the Ank2-IP3R2 interaction is required for astrocytic morphogenesis, we expressed a dominant-negative construct containing the ankyrin-binding domain (ABD) of IP3R2 in astrocytes. While this manipulation had little effect under basal conditions, it significantly impaired BDNF-dependent astrocytic morphogenesis, indicating that Ank2-IP3R2 binding contributes to astrocytic BDNF signaling.

Based on these results, we have revised the manuscript to more cautiously state that Ank2 supports IP3R2 stability and function, rather than serving as a "key regulator." We have also noted in the Discussion that other cellular processes may contribute to the impaired calcium dynamics in Ank2 cKO.

<u>Revised manuscript</u>

Results [lines 228-233]

Given that IP3R2 is the predominant isoform in astrocytes, we hypothesized that Ank2 supports IP3R2 function in mediating BDNF signaling. STRING analysis of Ank2 molecular function revealed strong enrichment for inositol 1,4,5-triphosphate-sensitive calcium-release channel activity and high association

scores with cytoskeletal proteins (Fig. 5a), suggesting a functional link to IP3Rs. Co-immunoprecipitation from brain lysates confirmed Ank2 binding to IP3R1, as previously reported[43], and additionally to IP3R2, with minimal binding to IP3R3 (Fig. 5b). IP3R2 protein levels were significantly reduced in hippocampal astrocytes of Ank2 cKO mice, indicating that Ank2 contributes to the expression of IP3R2 (Supplementary Fig. 6a, b).

Results [lines 248-258]

To specifically interrogate the functional relevance of the Ank2-IP3R2 interaction, we targeted the ankyrin-binding domain (ABD) located in residues 955-991, which is conserved among IP3Rs[46]. We expressed the IP3R2 ABD as a dominant-negative construct (AAV-GFAP-eGFP-P2A-IP3R2 ABD) in astrocytes (Fig. 5i-l, Supplementary Fig. 7a, b). Expression of IP3R2 ABD alone did not alter GFAP intensity (Supplementary Fig. 7a, b). We next asked whether expression of IP3R2 ABD alters BDNF-dependent astrocyte morphogenesis *in vivo*. Hippocampal infusion of BDNF (100 ng/0.4 μL) induced astrocyte morphogenesis (Supplementary Fig. 7c-e). Astrocytes expressing IP3R2 ABD exhibited significantly lower GFAP volume after BDNF infusion compared with controls (Fig. 5k, l).

Together, these results demonstrate that Ank2 supports IP3R2 function to mediate BDNF-dependent astrocyte morphogenesis. Given the established role of IP3R2 in L-LTP and remote memory[2], Ank2 emerges as a key regulator of astrocytic structural plasticity that underlies memory persistence.

Discussion [lines 448-451]

It should be acknowledged that the present study focused primarily on the BDNF-IP3R2 pathway, yet other mechanisms could also contribute to the observed phenotypes. Exploring these alternative possibilities will be necessary to fully understand the roles of Ank2 in astrocytic Ca^{2+} signaling and neuron-astrocyte interactions.

Minor points:

• The author should consider changing the title, which is more specific to the fear memory persistence. For example, memory can be classified as aversive and pleasant memories, explicit and implicit memories, or long-term and short-term memories. If the authors give the paper a title, "Astrocytic Ankyrin2 Enables Memory Persistence", the experiments must be designed to investigate various types of memories; alternatively, the authors may want to be more specific in their title.

Response: We thank the reviewer for this important comment regarding the title. We also considered whether astrocytic Ank2 might be specific to fear-related memory or extend to other forms of memory. While it is not feasible to examine every type of memory within this study, we performed an additional object location memory test in Ank2 cKO mice. Although object location memory is typically retained for only ~2 days and thus is not generally categorized as remote memory, it is regarded as a hippocampus-dependent form of long-term memory. We found that Ank2 cKO mice failed to discriminate the changed object location, unlike controls, indicating that the role of astrocytic Ank2 is not restricted to fear memory but extends to other hippocampus-dependent long-term memories. Based on these findings, and in order

to specify the scope of our work more precisely, we have revised the title to highlight the hippocampal context of our study.

Revised manuscript

Title: Astrocytic Ankyrin2 Enables Memory Persistence in the Hippocampus

• In Fig. 7e, the authors show context specificity for the memory tested. The authors should show such specificity also for the data presented in Fig. 1 to rule out indiscriminate anxiety.

Response: In Figures 7 and 8, we specifically tested context specificity in order to evaluate whether BDNF- or astrocyte-mediated enhancement of memory persistence was context-dependent. In contrast, the experiment in Figure 1 was designed simply to assess whether memory for the shock-associated context itself was reduced, and therefore an alternative context was not included in this paradigm. Although we did not perform a context-specificity test in Figure 1, we and others have repeatedly demonstrated context-specific learning in contextual fear conditioning using naïve C57BL/6 mice, and we therefore expect that freezing levels in a novel context would have remained low, as no associative memory would have been formed.

• The Cre-LoxP system is a widely used tool for genetic modification in mouse models. However, the specificity of the virus needs to be quantified and shown in the paper. For example, the percentage of colocalization between astrocytes and the viral marker. Will hippocampus-specific deletion of astrocytic Ank2 decrease the number or volume of astrocytes? The images in Fig. 1.j are not enough to show the specificity. Please give the calculation results and add a higher magnification of the colocalized examples.

Response: We thank the reviewer for raising this important point. Although the Cre virus we used has been validated in a previous study (DOI: 10.1038/s41467-023-42746-w), we re-confirmed its specificity in our experiments using immunohistochemistry and added these results to Supplementary Figure 3. We also realized that we had not previously noted the presence of a nuclear localization signal (NLS) in this construct and have now clarified this in the manuscript. Because Cre contains the NLS, we performed colocalization analysis with Sox9 as an astrocyte marker and NeuN as a neuronal marker. The results confirmed astrocyte-specific expression of Cre. As suggested, we have updated the manuscript to include these findings, with higher-magnification images as well.

Revised manuscript

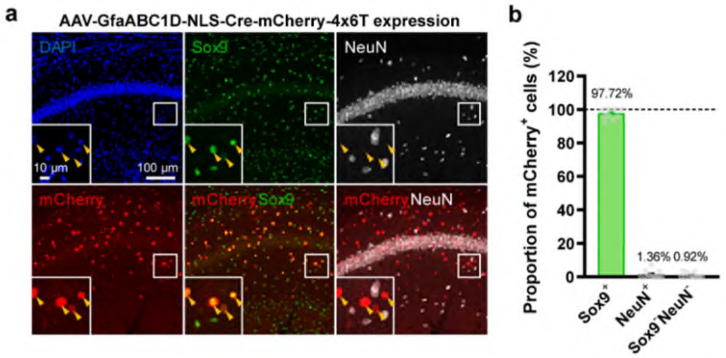

Supplementary Fig. 3. Specificity of AAV-GfaABC1D-NLS-Cre-mCherry-4x6T virus and quantification of Ank2 and Ezrin proteins as well as astrocyte number in the hippocampus. (a) Representative images of AAV-GfaABC1D-Cre-mCh-4x6T expression. Nuclei are stained with DAPI (blue), Cre-mCherry (red), astrocytes with Sox9 (green), and neurons with NeuN (gray). Yellow arrowheads indicate mCherry localization in Sox9-positive (Sox9$^+$) cells. (b) Quantification of mCherry$^+$ cells from 11 images across 4 mice. Dots indicate the proportion of mCherry$^+$ cells (%) in each image. Of 630 mCherry$^+$ cells, 618 were Sox9$^+$, 6 were NeuN$^+$, and 6 were negative for both Sox9 and NeuN.

• The authors did not describe the experimental steps for contextual fear conditioning in the hippocampal BDNF diffusion groups.

Response: We have now clarified this in Main text as well as the Methods section.

Revised manuscript

Results [lines 75-82]

Next, we evaluated learning and memory using contextual fear conditioning (Fig. 1c-g). During the acquisition phase, both control and Ank2^{GFAP} cKO mice exhibited similar responses to three electric shocks (Fig. 1d), indicating normal shock perception and immediate response. One day post-conditioning, memory recall (recent memory) showed no significant difference between groups (Fig. 1e). However, when remote memory recall was assessed 14 days post-conditioning, Ank2^{GFAP} cKO mice exhibited significantly reduced freezing behavior compared to controls (Fig. 1f), suggesting impaired remote memory. Memory persistence, calculated as the ratio of freezing on day 14 to freezing on day 1, was also significantly reduced in Ank2^{GFAP} cKO mice (Fig. 1g).

Methods [lines 508-534]

For both the recent and remote memory tests, a hexahedral conditioning chamber (18 cm wide × 18 cm long × 30 cm height; H10-11M-TC; Coulbourn Instruments) was used. The chamber consisted of a metal grid floor, aluminum sidewalls, and a clear plexiglass front door and back wall. In the conditioning protocol, after an acclimation period of 208 seconds, mice were exposed to a strong unconditioned stimulus consisting of three foot-shocks (0.75 mA, 2 s each) delivered at 30-second intervals. Following the final foot-shock, the mice remained in the conditioning chamber for an additional 30 seconds before being returned to their home cages. For the recent memory test, conducted 24 hours post-conditioning, the mice were placed in the conditioned context chamber for 5 minutes, during which freezing behavior was

measured. For the remote memory test, conducted 14 days post-conditioning, the same procedure was repeated. Freezing behavior was analyzed using Freezeframe software, and memory persistence was assessed by calculating the ratio of freezing time on day 14 to day 1.

For the extinction test, the protocol consisted of 4 days. On the first day, after an acclimation period of 208 seconds, mice were exposed to three foot-shocks (0.75 mA, 2 s each) delivered at 30-second intervals. On the following day, mice were placed back into the conditioned context chamber for 30 minutes. Freezing behavior was measured every 5 minutes, resulting in 6 blocks. The same procedure was repeated on the third day. On the final day, freezing behavior was measured for 5 minutes and compared to the first block on the second day to calculate memory extinction.

For remote memory enhancement with BDNF infusion or Opto-T1, we used a mild conditioning protocol (single foot-shock, 0.75 mA, 2 s) following a 208-s acclimation period, as previously described[48]. Mice remained in the chamber for 30 s after the shock before returning to their home cages. Twelve hours after acquisition, either BDNF (100 ng in 0.4 μl per side, delivered bilaterally at 0.2 μl/min) or optogenetic stimulation was applied. Detailed surgical procedures for BDNF infusion are described in Cannula implantation and infusion of BDNF in the hippocampus. Non-invasive blue light stimulation (~0.5 mW/mm^2 for 10 min) was delivered using a custom blue-light LED cage lid (Scitech Korea), with minor modifications from a previously described design[49]. Recent and remote memory were assessed 24 h and 14 days after conditioning, respectively, by measuring freezing for 5 min in the conditioned context. To test context specificity, mice were exposed to a novel cylindrical arena (20 cm diameter, 25 cm height) with bedding on the floor for 5 min one day after the remote memory test.

• In Fig.7 and Fig. 8.f-j, the authors used a similar protocol to generate fear memory in mice. However, the freezing time for the control groups is different (BDNF control group: ~27%, optic control group: ~50%) in the 1 day after conditioning contextual tests. The authors should discuss the potential reason in the discussion part or explain it with references.

Response: We appreciate the reviewer 3's insightful comment. As noted, the baseline freezing levels in the control groups differed between Figure 7 (BDNF experiment) and Figure 8 (Opto-T1 experiment). The major procedural difference is that, 12 hours after learning, BDNF was infused in Figure 7, whereas in Figure 8 mice in the home cage received non-invasive blue light stimulation. A potential reason is that blue light can be perceived by the mouse, and previous study has reported that blue light exposure may induce mild anxiety-like responses (10.1016/j.neuroimage.2025.1212). Such effects could contribute to the elevated baseline freezing in the optic experimental groups in Figure 8. Importantly, however, both the control and Opto-T1 groups in Figure 8 were exposed to identical blue light conditions, and no differences were observed at day 1. The divergence appeared only at day 14, strongly supporting our main conclusion that astrocyte-specific Opto-T1 activation influences memory persistence, despite the apparent difference in basal responses compared with the BDNF infusion experiments.

• The authors need to list all statistical results for every figure. For example, the author should apply a suitable statistical analysis between groups (e.g., two-way ANOVA) for Fig. 1.d, h, and k, Fig. 2.d, and Supple. 5a.

Response: We thank the reviewer for this comment. All statistical results, including the appropriate analyses between groups, were provided in Table S1. We also recognize that readers may not always refer to supplementary tables. To improve clarity, we have now included the relevant statistical details directly in the figure legends, within the journal's 350-word limit per legend.

• The authors should provide an overview image of injection and implantation sites in the mouse brain for each mouse.

Response: We have now added additional images of the injection and implantation sites where they were missing, together with the detailed descriptions of the virus injection and implantation protocols.

Revised manuscript

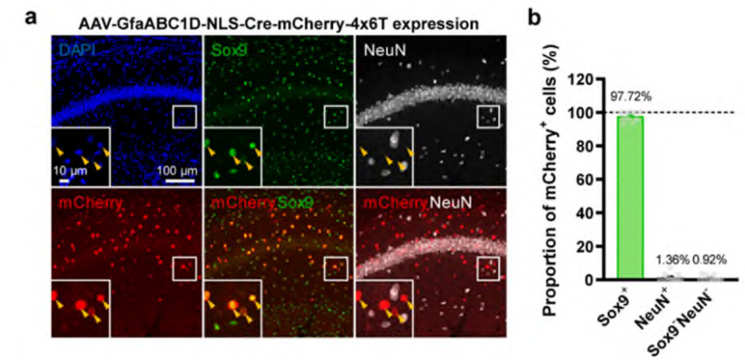

Supplementary Fig. 3. Specificity of AAV-GfaABC1D-NLS-Cre-mCherry-4x6T virus and quantification of Ank2 and Ezrin proteins as well as astrocyte number in the hippocampus. (a) Representative images of AAV-GfaABC1D-Cre-mCh-4x6T expression. Nuclei are stained with DAPI (blue), Cre-mCherry (red), astrocytes with Sox9 (green), and neurons with NeuN (gray). Yellow arrowheads indicate mCherry localization in Sox9-positive ($Sox9^+$) cells. (b) Quantification of $mCherry^+$ cells from 11 images across 4 mice. Dots indicate the proportion of $mCherry^+$ cells (%) in each image. Of 630 $mCherry^+$ cells, 618 were $Sox9^+$, 6 were $NeuN^+$, and 6 were negative for both Sox9 and NeuN.

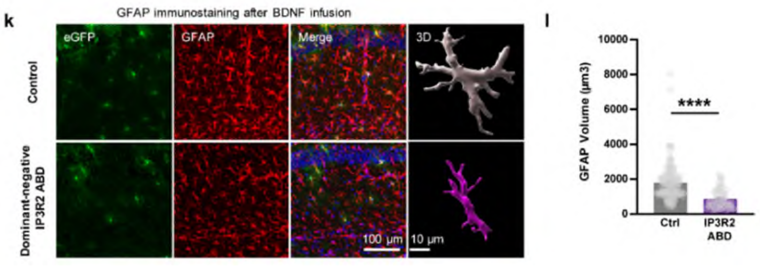

Fig. 5. Ank2 interacts with IP3R2 and regulates astrocytic Ca2+ signaling and BDNF-dependent morphogenesis. (k) Representative images of GFAP immunostaining and eGFP signals in hippocampal tissue following BDNF infusion (100 ng/0.4 µL). (l) Quantification of GFAP volume in astrocytes expressing control or IP3R2 ABD constructs after BDNF infusion. Control group, n = 167 astrocytes from 3 mice; IP3R2 ABD group, n = 75 astrocytes from 3 mice. Data are mean ± SEM. $^*P < 0.05$, $^{**}P < 0.01$, $^{****}P < 0.0001$; ns, not significant. Mann-Whitney test (e-h) and Welch's t-test (l). Detailed statistics are provided in Table S1.

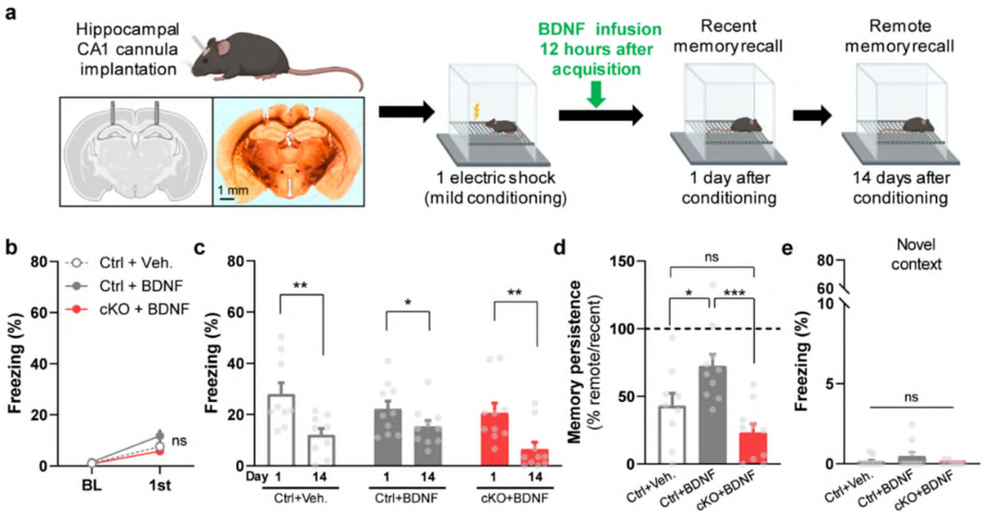

Fig. 7. BDNF-induced memory persistence is reduced in astrocyte-specific Ank2-deleted mice. (a) Schematic diagram of contextual fear conditioning with BDNF infusion into the hippocampal CA1. Mild conditioning (one electric shock instead of three) was used to assess BDNF-induced memory persistence. (b-e) Freezing behavior measured across different phases: (b) during acquisition, (c) in the recent and remote memory test. (d) Memory persistence in control mice with vehicle (aCSF) or BDNF (100ng/0.4μL/side) infusion, and in Ank2^{GFAP} cKO mice with BDNF infusion into hippocampal CA1 12 hours after memory acquisition. (e) Freezing behavior in the novel context test. Control+vehicle group, n = 9 mice; Control+BDNF group, n = 10 mice; Ank2^{GFAP} cKO+BDNF group, n = 10 mice. Data are mean ± SEM. *P < 0.05, **P < 0.01, ***P < 0.001. ns, not significant. Two-way repeated measures ANOVA (b), paired t-test (c, control+vehecle and control+BDNF), Wilcoxon test (c, Ank2 cKO+BDNF), Tukey's multiple comparisons test (d), and Dunn's multiple comparisons test (e). Detailed statistics are provided in Table S1.

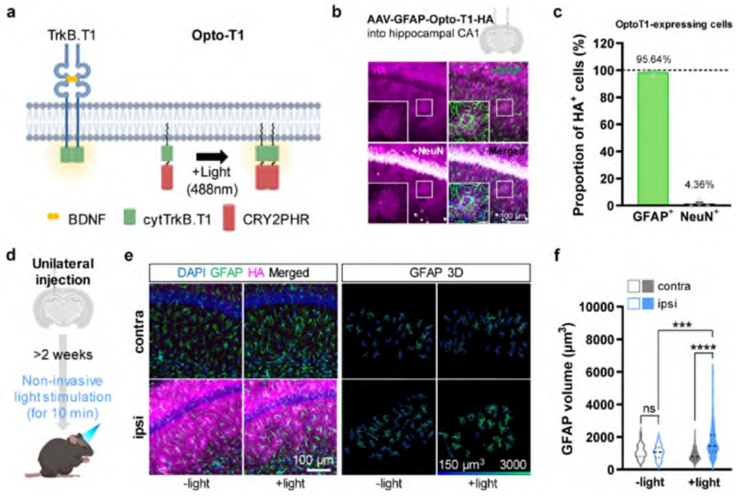

Fig. 8. Astrocyte-specific BDNF signaling sufficiently induces memory persistence. (a) Design of Opto-cytTrkB.T1 (Opto-T1), containing a light-sensitive CRY2PHR^{E281A} domain fused to the cytosolic domain of the TrkB.T1 receptor. (b) Representative image showing astrocyte-specific expression of AAV-GFAP-Opto-T1-HA. (c) Quantification of astrocytic specificity of Opto-T1 expression. Each dot indicates one slice. A total of 311 HA^{+} cells were analyzed from 4 slices across 3 mice. (d) Experimental scheme for unilateral virus injection and non-invasive continuous blue light stimulation (0.5 mW/mm^2 in the home cage). (e) Representative GFAP and HA immunostaining of contralateral (non-injected) and ipsilateral (Opto-T1-injected) hippocampal CA1. (f) Comparison of GFAP volume without (-light, n = 3 mice; Contra, 32 astrocytes; Ipsi, 123 astrocytes) or with (+light, n = 3 mice; Contra, 49 astrocytes; Ipsi, 88 astrocytes) light stimulation

• What does the abbreviation “> 14 DPI” mean in Fig. 8.b and Supplementary Fig. 6.a?

Response: We have used DPI is an acronym for Days Post-Injection. But, to prevent confusion, we have changed the term from “>14 DPI” to “> 2 weeks”.

• The author should describe the laser delivery strategy in the methods part. How long is the laser stimulus? Is that pulsed or continuous stimulation? What is the wavelength used for the blue light?

Response: We have now clarified the laser delivery strategy in the Main text and Methods section.

Revised manuscript

Results [lines 356-357]

For non-invasive activation of Opto-T1, we placed a customized LED lid over the home cage, which delivered continuous blue light stimulation (~0.5 mW/mm^2, 10 min) to freely moving mice.

Results [lines 365-368]

In hippocampal slices from Opto-T1-expressing mice, continuous blue light stimulation (50 $\mu W/mm^2$, 10 min, 1 h post-3×TBS) likewise maintained LTP for at least 3 h (Fig. 8g, h), indicating that astrocytic TrkB.T1 activation is sufficient to sustain LTP maintenance.

Methods [lines 529-531]

Non-invasive blue light stimulation (~0.5 mW/mm^2 for 10 min) was delivered using a custom blue-light LED cage lid (Scitech Korea), with minor modifications from a previously described design[49].

Methods [lines 663-664]

To activate Opto-T1 expressed in astrocytes, blue light (473 nm; 50 $\mu W/mm^2$, MBL-III-473 nm, CNI Laser) was delivered for 10 minutes starting 1 hour after 3×TBS stimulation.

• In Fig. 2, Fig. 3, Fig. 4, Fig. 5, Fig. 6, and Fig. 8.e, the authors need to show the number of mice in the groups and calculate the mean value from each mouse instead show the whole group of data was gathered from slices, astrocytes, soma, or microdomains. It is less convincing if the data came from one or two mice.

Response: We appreciate the reviewer’s comment. We have now included the number of mice for each experiment in addition to the numbers of cells and slices, and this information is provided in Table S1. To improve accessibility for readers, we have also added the number of mice directly in the figure legends, while keeping within the journal’s 350-word limit per legend.

• The authors used similar schematic figures to show the virus injection in Fig. 1.j, Fig. 4.d, Fig. 5.b, Fig. 8.b. The authors need to make the figures clear enough for the reader to understand their experimental design. Please show the unilateral and bilateral injections clearly in the figures.

Response: We have revised the schematic figures to make them clearer and to explicitly indicate whether unilateral or bilateral injections were used. This information has also been clarified in the figure legends and Methods section.

• Please replace Supplementary Figure 3.c with a higher resolution image. (The result of the control #1 looks a bit artificial.)

Response: Supplementary Figure 3e (previously 3c) has been replaced with a higher-resolution image.

Revised manuscript

Supplementary Figures

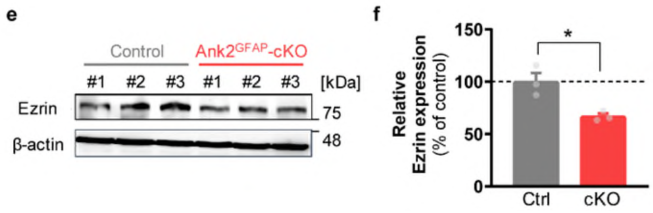

• Please do not compress the data points in Fig. 7.e and Fig. 8.j. The authors need to find a suitable y axis range to show the data clearly in the figures.

Response: In the revised figures, we have introduced a y-axis break in Fig. 7e and Fig. 8j so that lower freezing values are more clearly visible while still allowing direct comparison with the higher values.

• In Supplementary Fig. 7, the authors wrote: "Astrocytic Ank2 plays a distinct role in maintaining IP3R2 expression and function within the TrkB.T1-IP3R2 axis, which is essential for astrocyte morphogenesis via cytoskeletal rearrangements". However, it is not consistent with the figure described as IP3Rs. The authors need to summarize the figure and consistently provide a description.

Response: We have carefully re-checked the description and revised the text to ensure consistency across the figure, legend, and main text.

Finally, we would like to express our sincere gratitude to the reviewers not only for their major comments but also for their careful minor suggestions. We found these comments highly constructive, and they have helped us to improve the clarity, consistency, and overall quality of the manuscript.

Reviewer #2_second round revision (Remarks to the Author):

The authors have done a good revision to address my concerns. Now I don't have any major concerns regarding the current form of the manuscript. The only thing that I still would like to note is that : they stated in the response letter point 5 that "In addition, because we observed reduced IP3R2 expression in Ank2 cKO astrocytes, approaches such as DREADD-mediated activation of Gq, one of the upstream regulators of IP3R2, would not be appropriate." I feel this argument might be too strong. The IP3R2 reduction is minimal in cKO mice as shown in Fig S6. While I am not insisting on performing DREADD-based experiments, I do think the authors should include a brief discussion on rescuing morphology using DREADD approach as a potential future research direction before the publication of the current form.

Response

We thank the reviewer for this thoughtful and constructive comment. Although IP3R2 expression is modestly reduced in Ank2 cKO astrocytes (Supplementary Fig. S6), we acknowledge that chemogenetic activation of upstream Gq signaling could still, in principle, provide a useful avenue for future investigation. In response to this helpful suggestion, we have revised the Discussion to include a brief statement noting DREADD-mediated astrocytic activation as a potential future strategy to test whether restoring astrocytic activity can rescue the morphological and behavioral phenotypes observed in $Ank2^{GFAP}$ cKO mice. We appreciate the reviewer's guidance, which has helped us clarify the scope of the current study and more accurately frame productive directions for future work.

Revised manuscript

DISCUSSION

It should be acknowledged that the present study focused primarily on the BDNF-IP3R2 pathway, yet other mechanisms could also contribute to the observed phenotypes. In this context, chemogenetic astrocytic activation (e.g., DREADDs) may offer a complementary approach to test whether memory persistence can be rescued in $Ank2^{GFAP}$ cKO mice.

Reviewer #3_second round revision (Remarks to the Author):

I appreciate the authors' efforts in addressing the points raised by us. Many points have now been addressed, but we feel that further additions to text, analysis, and additional experimental data are still required to support the claims of the manuscript. Most importantly, the feasibility of non-invasive blue light stimulation of the authors' Opto-T1 construct needs to be convincingly demonstrated.

Response

We sincerely thank the reviewer #3 for the careful evaluation of our revised manuscript. We agree that the points highlighted were valid and constructive. In response, we conducted additional experiments, performed further analyses, and revised the manuscript accordingly to address these issues as thoroughly as possible. We believe that these additions have substantially strengthened the rigor, clarity, and overall robustness of the study. Our detailed responses to each point, along with corresponding revisions to the manuscript, are provided below.

1. The authors should clarify if bilateral or unilateral injections were used for Fig. 1j-n. Fig. 1j suggests bilateral, but I cannot find references to this in methods/results. The authors are mentioning in their response a possible role of Ank2 in ACC or mPFC to explain the relatively weak CA1-specific deletion, but the suggested discussion points evade this topic. The authors should point out in their discussion the relatively lower levels with reference to other brain regions that may be involved.

Response

We have now explicitly stated that the injections in Fig. 1j-n were bilateral and clarified this in the Results, figure legend, and methods. The Discussion section has been revised to more clearly acknowledge the potential contribution of astrocytes in other brain regions, including the ACC and mPFC.

Revised Manuscript

RESULTS

we bilaterally injected an adeno-associated virus (AAV) carrying Cre recombinase under the GFAP promoter (AAV-GfaABC1D-NLS-Cre-mCh-4x6T) into the CA1 region of Ank2$^{fl/fl}$ mice.

Figure 1 legend

(j) Bilateral Hippocampal hippocampal astrocyte-specific Ank2 deletion.

DISCUSSION

While hippocampal CA1-restricted deletion of astrocytic Ank2 was sufficient to impair memory persistence, the behavioral deficit was more modest than that observed following astrocyte-specific Ank2 deletion using GFAP-CreER^{T2}. This difference suggests that astrocytic Ank2 in additional brain regions may also contribute to memory persistence, particularly in cortical areas such as the medial prefrontal cortex and anterior cingulate cortex, which are known to support remote memory storage.

METHODS

Viruses were injected using syringe pump (KD Scientific, Holliston, MA, USA), bilaterally unless otherwise specified, into CA1 of hippocampus with the following coordinates (from bregma).

2. I disagree with the authors' interpretation that their data in Fig. 6c-e demonstrate "impaired BDNF-dependent astrocyte morphogenesis". To claim this, the authors would need to run a 2-way ANOVA that specifically tests for interaction effects of BDNF and cKO. Judging from these panels, I would guess that the ANOVA would reveal a significant main effect for both BDNF as well as the knockout condition, but might fail to reveal interaction effects. This would be interpreted as BDNF increasing GFAP volume and cKO animals showing less volume. Only an interaction effect would demonstrate that BDNF-dependent increases in GFAP volume are impaired in the cKO condition, not the currently displayed two Mann-Whitney tests and a bar plot in (e) without statistics.

Response

We thank the reviewer for this important and constructive suggestion for the interaction effect. As the reviewer recommended, we performed a two-way ANOVA with genotype (control vs. cKO) and treatment (vehicle vs. BDNF) as factors. This analysis revealed a significant interaction between genotype and BDNF treatment (P = 0.0084), demonstrating that the BDNF-dependent increase in GFAP volume is indeed impaired in the Ank2 cKO condition.

**Two-way ANOVA**	Ordinary				
Alpha	0.05				
**Source of Variation**	**% of total variation**	**P value**	**P value summary**	**Significant?**	
Interaction	2.341	0.0084	**	Yes	
Row Factor_BDNF	15.17	<0.0001	****	Yes	
Column Factor_Genotype	22.08	<0.0001	****	Yes	
**ANOVA table**	**SS (Type III)**	**DF**	**MS**	**F (DFn, DFd)**	**P value**
Interaction	11641859	1	11641859	F (1, 182) = 7.095	P=0.0084
Row Factor_BDNF	75467342	1	75467342	F (1, 182) = 45.99	P<0.0001
Column Factor_Genotype	109811349	1	109811349	F (1, 182) = 66.92	P<0.0001
Residual	298633014	182	1640841		

Thus, in contrast to the reviewer's expectation, the effect of BDNF on astrocyte morphogenesis is not preserved in the cKO mice but is significantly attenuated, supporting our interpretation of impaired BDNF-dependent astrocyte morphogenesis. We have updated the manuscript to include this two-way ANOVA and revised the corresponding Results and figure legend accordingly. We are grateful to the reviewer for this suggestion, which allowed us to strengthen the statistical rigor of the analysis and more clearly demonstrate our central hypothesis.

Revised Manuscript

RESULTS

A two-way ANOVA revealed a significant interaction between genotype and BDNF effect (P = 0.0084),

and accordingly, the GFAP volume in Ank2^{GFAP} cKO mice was reduced by 61.3% compared to controls following BDNF treatment (Fig. 6e), demonstrating that Ank2 is critical for BDNF-dependent astrocyte morphogenesis.

Figure 6 legend
(c, d) Quantification of GFAP volume in control and Ank2^{GFAP} cKO mice following treatment with either (c) boiled or (d) active BDNF for 30 minutes, followed by an additional 30-minute incubation in aCSF. Control+boiled BDNF group, n = 65 astrocytes from 2 mice; cKO+boiled BDNF group, n = 34 astrocytes from 2 mice. Control+BDNF group, n = 50 astrocytes from 2 mice; cKO+BDNF group, n = 37 astrocytes from 2 mice (two-way ANOVA, genotype x BDNF effect, P = 0.0084).

3. Again, I strongly disagree with the authors' interpretation of the data and their statement that "we did not include a separate cKO+vehicle group, as this condition was not expected to yield remote memory either". To test whether BDNF-induced memory is significantly affected in the cKO condition, the authors would need to show an interaction effect in a two-way ANOVA with factors ctrl/cKo and Veh/BDNF. To show this, the control of cKO+vehicle is thus critical. The two "Ctrl" conditions demonstrate that BDNF usually leads to an approximate 1.5x increase in memory persistence, but to show that this BDNF-dependent memory persistence increase fails in cKO mice, we need to know the vehicle memory persistence levels. This data is missing. If, as the authors suggest in their response, no memory persistence was to be expected, say a level of perhaps 10%, then the cKO+BDNF value might still represent an increase, perhaps also of 1.5x. Otherwise, it remains simply unknown if cKO animals simply have bad memory persistence per se or whether this memory persistence deficit is BDNF-dependent. Hence, such a condition and test are crucial to the authors' main hypothesis.

Response
We thank the reviewer for this critical and insightful comment. In direct response to this concern, we performed the additional experiment including the previously missing cKO + vehicle group and reanalyzed the data using a two-way ANOVA with genotype (control vs. Ank2 cKO) and treatment (vehicle vs. BDNF) as factors.

**Two-way ANOVA**	Ordinary				
Alpha	0.05				
**Source of Variation**	**% of total variation**	**P value**	**P value summary**	**Significant?**	
Interaction	20.18	0.0036	**	Yes	
Row Factor_BDNF	0.004037	0.9648	ns	No	
Column Factor_Genotype	9.722	0.0365	*	Yes	
**ANOVA table**	**SS (Type III)**	**DF**	**MS**	**F (DFn, DFd)**	**P value**
Interaction	7748	1	7748	F (1, 33) = 9.857	P=0.0036
Row Factor_BDNF	1.550	1	1.550	F (1, 33) = 0.001972	P=0.9648
Column Factor_Genotype	3733	1	3733	F (1, 33) = 4.750	P=0.0365
Residual	25940	33	786.0		

This analysis revealed a significant genotype x BDNF interaction (P = 0.0036), demonstrating that the effect of BDNF on memory persistence differs depending on astrocytic Ank2. While genotype showed a significant main effect (P = 0.0365), the main effect of BDNF was not significant (P = 0.9648), due to the opposing BDNF effects across genotypes. Importantly, memory persistence in the cKO + BDNF group was further reduced compared to the cKO + vehicle group. This result directly addresses the reviewer's concern and indicates that the reduced memory persistence observed in Ank2 cKO mice cannot be attributed simply to a lower baseline performance.

We acknowledge that this outcome was not anticipated based on our initial hypothesis. While the underlying mechanism remains unclear, one possible explanation is that hippocampal BDNF infusion may exert non-cell-type-specific effects, engaging neuronal or other glial signaling pathways that normally require coordinated astrocytic support. In the absence of astrocytic Ank2, such signaling may fail to be properly integrated, resulting in an apparent lack of memory support rather than enhancement.

Together, these results show that BDNF-dependent memory persistence critically depends on astrocytic Ank2, rather than reflecting a nonspecific impairment of memory in cKO animals. The corresponding Results and figure legends have been revised to include the two-way ANOVA and to reflect this interaction effect.

Revised Manuscript

RESULTS

In control mice, BDNF infusion significantly enhanced memory persistence, whereas $Ank2^{GFAP}$ cKO mice failed to show this enhancement and instead exhibited reduced memory persistence despite receiving the same treatment (Fig. 7d; two-way ANOVA, genotype x BDNF effect interaction, P = 0.0036).

Figure 7

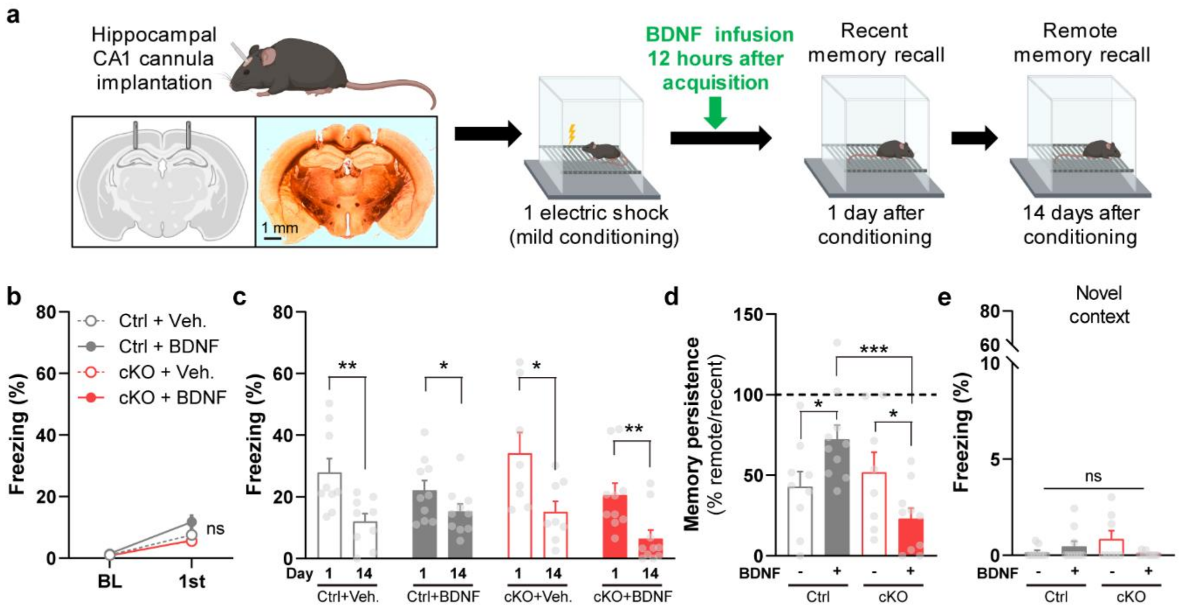

Fig. 7. BDNF-induced memory persistence is reduced in astrocyte-specific Ank2-deleted mice. (a) Schematic diagram of contextual fear conditioning with BDNF infusion into the hippocampal CA1. Mild conditioning (one electric shock instead of

three) was used to assess BDNF-induced memory persistence. (b-e) Freezing behavior measured across different phases: (b) during acquisition, (c) in the recent and remote memory test. (d) Memory persistence in control mice with vehicle (aCSF) or BDNF (100ng/0.4μL/side) infusion, and in Ank2^{GFAP} cKO mice with BDNF infusion into hippocampal CA1 12 hours after memory acquisition (two-way ANOVA, genotype x BDNF effect, $P = 0.0036$). (e) Freezing behavior in the novel context test. Control+vehicle group, n = 9 mice; Control+BDNF group, n = 10 mice; Ank2^{GFAP} cKO+BDNF group, n = 10 mice. Data are mean ± SEM. *$P < 0.05$, **$P < 0.01$, ***$P < 0.001$. ns, not significant. Two-way repeated measures ANOVA (b), paired t-test (c, control+vehecle and control+BDNF), Wilcoxon test (c, Ank2 cKO+BDNF), two-way ANOVA with post hoc multiple comparisons (d), and Dunn's multiple comparisons test (e). Detailed statistics are provided in Table S1.

4. I thank the authors for their clarification and the additional LTP experiments. It remains unfortunate that the final piece of evidence is missing any link to Ank-2, but I agree that further experiments along these lines would likely go beyond the current scope.

Response

We also thank the reviewer for this thoughtful assessment. We agree that establishing a more direct mechanistic link represents an important direction, and we plan to address this in future studies.

5. I thank the authors for clarifying their data but suggest they also offer these explanations to the readers of the manuscript. That is, the freezing data should find its way to the supplementary and the implications, as described in the authors' response in the previous paragraph, should find its way into the discussion.

Response

We thank the reviewer for this helpful suggestion. The corresponding freezing data have now been included in the Supplementary Information, and the relevant explanations and implications have been briefly incorporated into the Discussion.

Revised Manuscript

RESULTS

Although recent memory did not differ between groups (Fig. 1 and supplementary Fig. 5c), astrocyte-eGRASP puncta density was significantly reduced in Ank2^{GFAP} cKO mice (Fig. 4h, i).

DISCUSSION

Further validating astrocyte-eGRASP at remote time points will be important to determine how astrocytic contacts on engram neurons are maintained during long-term memory consolidation.

SUPPLEMENTARY INFORMATION

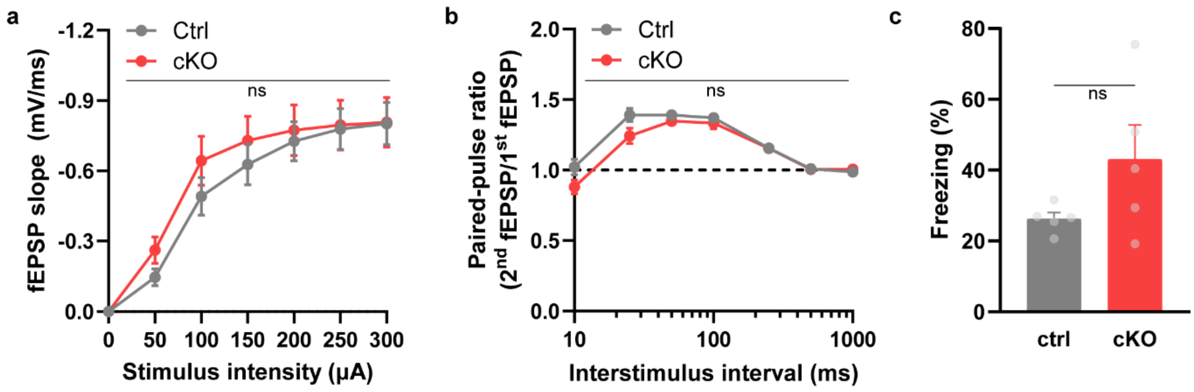

Supplementary Fig. 5. Basal synaptic properties in Ank2^{GFAP} cKO mice and recent memory performance in astrocyte-eGRASP experiments. (a) Input-output curve showing the field excitatory postsynaptic potential (fEPSP) slope from Schaffer collateral pathway in response to increasing stimulus intensities in control and Ank2^{GFAP} cKO mice. (b) Paired-pulse ratio (PPR) measured at inter-stimulus intervals of 10, 25, 50, 100, 250, 500, and 1000 ms in control and Ank2^{GFAP} cKO mice. Control group, n = 12 slices from 3 mice; Ank2^{GFAP} cKO group, n = 12 slices from 3 mice. (c) Freezing behavior during the recent memory test (Day 1) in control and Ank2^{GFAP} cKO mice used for astrocyte-eGRASP analysis in Fig. 4g-i. Astrocyte-eGRASP was assessed at this recent time point because the long-term stability of doxycycline-based eGRASP labeling has not yet been fully established[10]. Control group, n = 5 mice; Ank2^{GFAP} cKO group, n = 5 mice. Data are mean ± SEM. Unpaired t-test (a, b, c). Detailed statistics are provided in Table S1.

6. I appreciate the authors' additional work on establishing the links between Ank2 and IP3R2. As before, however, to show BDNF-dependent deficits on GFAP volume in these knockouts, the authors need to perform two-way ANOVAs that contain main effects for BDNF/vehicle as well as Ctrl/IP3R2 ABD, and test for interaction effects. This is necessary to establish that the BDNF-derived volume increases are not seen in IP3R2 ABD mice, and not just a matter of reduced baseline GFAP volume levels.

Response

We thank the reviewer for this careful and constructive comment. We agree that a two-way ANOVA testing the interaction between treatment (BDNF vs. vehicle) and condition (control vs. IP3R2-ABD) would, in principle, be the most direct approach to assess whether BDNF-dependent increases in GFAP volume are specifically abolished in the IP3R2-ABD condition. We note, however, that baseline GFAP measurements in control and IP3R2-ABD astrocytes (Supplementary Fig. 7a,b) were obtained under conditions without cannula implantation or vehicle infusion, whereas the BDNF-induced morphogenesis experiments (Fig. 5k,l) were performed after cannula implantation followed by BDNF infusion. Because these datasets were generated under distinct experimental conditions, we consider a direct two-way ANOVA across baseline and BDNF-treated groups to be statistically inappropriate.

To avoid overinterpretation, we have therefore revised the text to describe this result more conservatively. In the revised manuscript, we state that astrocytes expressing IP3R2-ABD exhibit reduced GFAP volume under BDNF infusion, without making claims regarding a genotype x BDNF treatment interaction.

Revised Manuscript

RESULTS

One hour after BDNF infusion, astrocytes expressing IP3R2 ABD exhibited significantly lower GFAP volume compared with controls (Fig. 5k, l).

7. I appreciate the authors' discussion points and would like to suggest the authors share these with other readers in the manuscript's discussion section, as originally suggested.

Response

We thank the reviewer for this suggestion. The relevant discussion points have now been incorporated into the Discussion section of revised manuscript.

Revised Manuscript

DISCUSSION

It should be noted that non-invasive blue light stimulation itself may influence baseline freezing levels[56], and therefore comparisons should be made between groups exposed to identical light conditions.

8. I have only now become aware that the authors are indeed using "non-invasive blue light stimulation" through – presumably – scalp, skull, cortex, to reach the Opto-T1 construct in hippocampal area CA1 while animals are freely moving in a home cage with an LED lid. I fail to fathom how the authors can reach sufficient intensities on the level of the hippocampus with such non-invasive stimulation, as I presume 0.5 mW/mm2 refers to the levels of light on the head, but not inside the hippocampus. The authors need to credibly show that sufficient light intensities reach the hippocampus through this method. Otherwise, it is unclear why a non-invasive light delivery was chosen in this case. While there now exist methods for near-infrared non-invasive delivery, blue light is notoriously prone to scattering and absorption of biological tissue. I recommend the authors use direct optic light fiber-mediated light delivery, as is common for blue light stimulation of subcortical brain regions.

Response

We thank the reviewer for raising this important point regarding the feasibility of non-invasive blue light stimulation to activate Opto-T1 in hippocampal CA1. We fully agree that blue light has limited tissue penetration and that direct evidence of in vivo activation is essential. Although prior studies have demonstrated that Cry2PHR-based optogenetic actuators can be effectively activated transcranially at low intensities (e.g., ~50 μW/mm^2) due to their high photosensitivity (Hong & Heo, 2020; Jung et al., 2019; Kim et al., 2020; Hyeon et al., 2025), we recognized that additional empirical verification was necessary to address the reviewer's concern in the context of our specific experimental paradigm.

To validate that our stimulation parameters (0.5 mW/mm^2) are sufficient to activate Opto-T1 through the intact scalp, we performed an additional unilateral transcranial stimulation experiment now included as Supplementary Fig. 8. In this experiment, we expressed Opto-T1 broadly in astrocytes via retro-orbital

AAV-PHP.eB delivery, allowing us to directly read out Opto-T1 activation throughout the brain independent of local viral transduction efficacy. We then delivered the same non-invasive blue light intensity (473 nm, 0.5 mW/mm^2, 10 min) onto the scalp above the right hemisphere under anesthesia. The mouse was sacrificed 1 h later, and GFAP immunohistochemistry revealed a robust increase exclusively in the illuminated hemisphere, compared to the contralateral control hemisphere. This selective astrocytic response demonstrates that the light intensity used in our behavioral experiments is indeed sufficient to penetrate the scalp and skull and activate Opto-T1 in hippocampal CA1.

Importantly, we deliberately chose to avoid optic fiber implantation because previous studies have shown that fiber insertion can induce local astrogliosis and alter endogenous BDNF signaling (Albini et al., 2023; Chun et al., 2018), potentially confounding the interpretation of experiments specifically examining astrocytic structural plasticity and BDNF-dependent processes. Our aim was to use an approach that preserves the native astrocytic environment, especially because astrocyte morphology and BDNF-TrkB.T1 signaling are central to the study.

Together, these new results directly address the reviewer's concern and empirically confirm that non-invasive stimulation at 0.5 mW/mm^2 activates Opto-T1 in hippocampal CA1. We have added Supplementary Fig. 8 and the corresponding methodological details to the revised manuscript.

Please see also below references.

References for transcranial photoactivation with blue LED light home cage lids

- Hong, Jongryul, and Won Do Heo. "Optogenetic modulation of TrkB signaling in the mouse brain." *Journal of molecular biology* 432, no. 4 (2020): 815-827.
- Hyeon, Bobae, Jaehyun Shin, Jae-Hun Lee, Woori Kim, Jea Kwon, Heeyoung Lee, Dae-gun Kim et al. "Integrating artificial intelligence and optogenetics for Parkinson's disease diagnosis and therapeutics in male mice." Nature Communications 16, no. 1 (2025): 7797.
- Jung, Hyunjin, Seong-Wook Kim, Minsoo Kim, Jongryul Hong, Daseuli Yu, Ji Hye Kim, Yunju Lee et al. "Noninvasive optical activation of Flp recombinase for genetic manipulation in deep mouse brain regions." *Nature communications* 10, no. 1 (2019): 314.
- Kim, Sungsoo, Taeyoon Kyung, Jae-Hee Chung, Nury Kim, Sehoon Keum, Jinsu Lee, Hyerim Park et al. "Non-invasive optical control of endogenous Ca2+ channels in awake mice." *Nature communications* 11, no. 1 (2020): 210.

References (optic fiber implantation induces reactive gliosis, which can alter local astrocytic BDNF signaling, including BDNF synthesis and release)

- Albini, M. et al., 2023, Neuroscience Research. DOI:10.1016/j.neures.2023.02.001.
- Chun et al., 2018, Experimental Neurobiology. DOI: 10.5607/en.2018.27.3.155.

Revised Manuscript

SUPPLEMENTARY INFORMATION

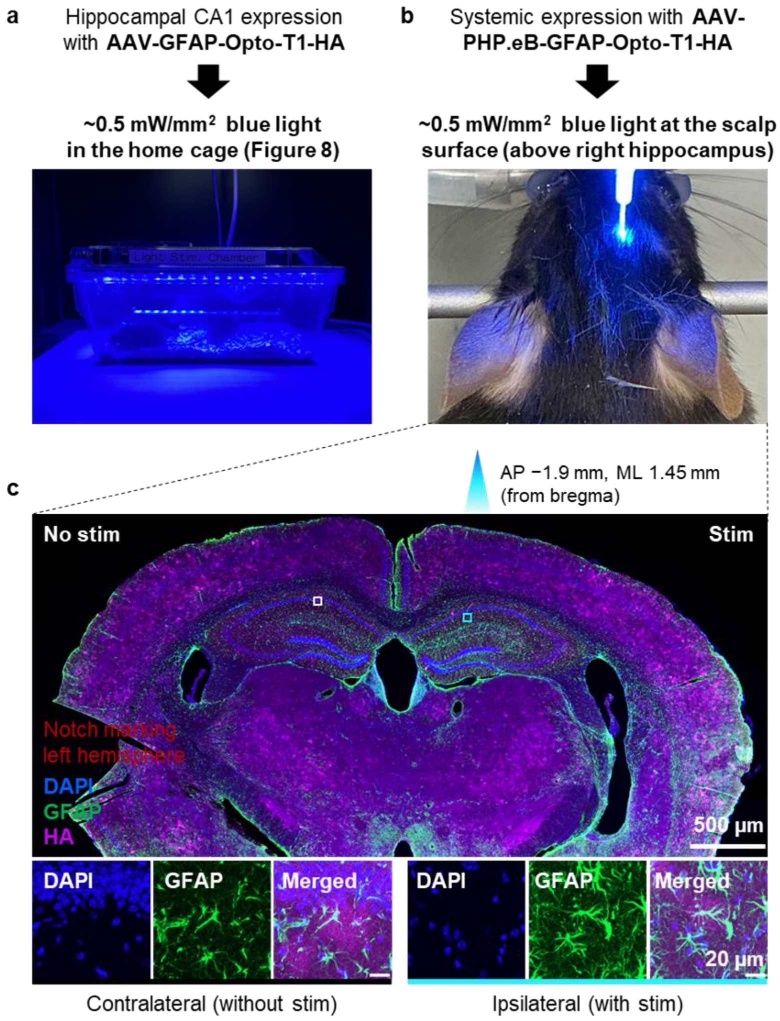

Supplementary Fig. 8. Non-invasive blue light stimulation (0.5 mW/mm^2) is sufficient to activate Opto-T1 through the intact scalp. (a) Non-invasive blue light stimulation setup in the home cage (0.5 mW/mm^2 through a custom LED lid) used in Figure 8. (b) To directly test light penetration, Opto-T1 was broadly expressed via retro-orbital AAV-PHP.eB-GFAP-Opto-T1-HA. Three weeks later, the blue light (473 nm, 0.5 mW/mm^2, 10 min) was applied to the scalp above the right hemisphere in an anesthetized mouse. The mouse was sacrificed 1 h after stimulation (the left hemisphere was notched for identification after fixation). (c) GFAP immunostaining revealed robust activation only in the illuminated hemisphere, demonstrating that 0.5 mW/mm^2 blue light is sufficient to activate Opto-T1 through the intact scalp and reach hippocampal CA1.

METHODS

Unilateral transcranial blue light stimulation for Opto-T1 activation

First, Opto-T1 was systemically expressed via retro-orbital injection of AAV-PHP.eB-GFAP-Opto-T1-HA, resulting in broad astrocytic expression across the brain. Three weeks later, the mouse was anesthetized with isoflurane. To accurately localize the stimulation site, the scalp was briefly incised to

expose the skull surface, stereotaxic coordinates were identified (AP -1.9 mm, ML +1.45 mm from bregma), and the scalp was sutured back prior to stimulation. Continuous blue light (473 nm, 0.5 mW/mm2, 10 min; MBL-III-473, CNI Laser) was then applied transcranially to the right hemisphere. The mouse was sacrificed 1 h after stimulation by transcardial perfusion. Following fixation, brains were extracted, and a notch was made in the left hemisphere to mark the unstimulated side for subsequent sectioning. Coronal sections (40 µm) were processed for immunohistochemistry, as described above.

Reviewer #3 (Remarks to the Author):
I thank the authors for the additional work they performed and presented. However, my concerns have not yet been fully addressed, as described below:

Response
We thank the reviewer for outlining the remaining concerns. We have carefully considered each point and provide detailed responses below.

1. Fig. 7: Panel b seems to be missing the "cKO + Veh." data? Panel c: As the authors state in their response, it is very surprising to find that BDNF application actually significantly reduces memory persistence. The authors should at least discuss this finding in their Discussion section and identify plausible hypotheses for this, based on literature. Otherwise, it may throw into doubt the mechanistic specificity of their cKO construct.

Response: We thank the reviewer for raising this important point. One possible explanation is that BDNF broadly enhances synaptic plasticity across neuronal populations, whereas astrocytic Ank2-dependent morphogenesis and astrocyte-synapse contacts may help confer circuit specificity to this plasticity. In the absence of Ank2, impaired astrocyte morphogenesis may reduce the ability of astrocytes to selectively support engram-related synapses upon BDNF infusion. Under these conditions, elevated BDNF signaling may promote less specific synaptic remodeling through TrkB receptors on overall neurons (not specific to engram neurons), which could ultimately destabilize the circuits supporting remote memory-specific persistence. We have added a discussion of this possibility in the revised manuscript.

In addition, there were cKO + Veh. data as shown below (cKO + Veh. is red circle with white fill and dotted line.). For clarity, we have revised the plot so that the data are not perceived as missing.

Revised manuscript

FIGURE 7

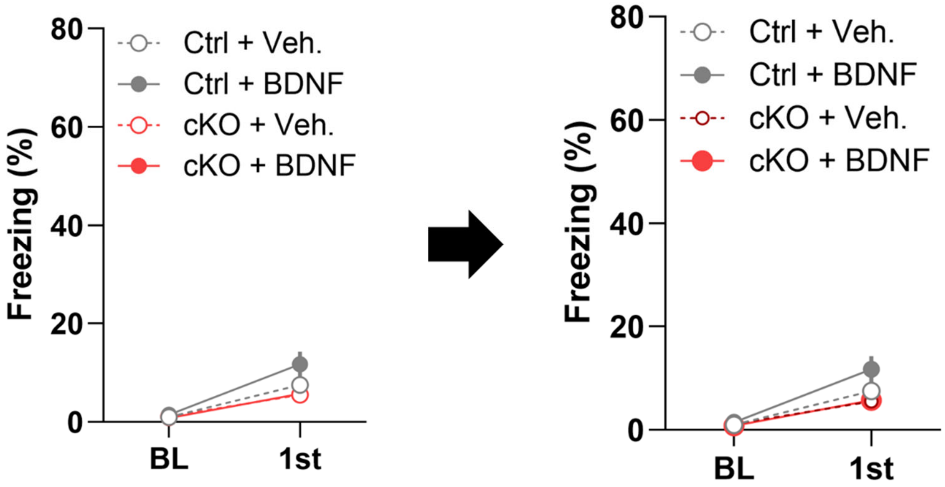

DISCUSSION

Notably, although BDNF infusion enhanced memory persistence in control animals, this effect was reversed in astrocytic Ank2 cKO mice. One possible explanation is that BDNF broadly promotes synaptic

plasticity, whereas astrocytic Ank2-dependent morphogenesis may provide circuit specificity by stabilizing astrocyte-synapse contacts at memory-related synapses. In the absence of Ank2, impaired astrocyte morphogenesis could reduce this selective stabilization, allowing elevated BDNF signaling to drive less specific synaptic remodeling that ultimately destabilizes circuits supporting remote memory persistence.

2. Fig. 5k-l: The authors claim that an ANOVA would be "statistically inappropriate" here if data from Fig. S7a,b were compared to the data presented here. Such a comparison has not been suggested. As I have pointed out, to make claims about "BDNF-dependent" effects, the inclusion of a vehicle control group would be necessary. The authors write that such claims have been toned down but, for example, the figure title still states "Ank2 interacts with IP3R2 and regulates astrocytic Ca2+ signaling and BDNF-dependent morphogenesis."

Response: We thank the reviewer for pointing this out and apologize for the imprecise wording that remained in the previous version. We now have revised the wording in the Fig. 5 title and the description. Specifically, "BDNF-dependent morphogenesis" have been changed to "BDNF-induced morphogenesis" and the Fig. 5 title has been revised to "Ank2 interacts with IP3R2 and regulates astrocytic Ca^{2+} signaling and astrocyte morphogenesis following BDNF infusion."

Revised manuscript

Fig. 5. Ank2 interacts with IP3R2 and regulates astrocytic Ca2+ signaling and morphogenesis following BDNF infusion.

3. Fig. S8: I appreciate the authors' efforts to provide more evidence that their Opto-T1 construct works. The data provided are insufficient to gauge the efficacy of this construct though.

a. First, the data come from a single animal and show a single brain slice. I see no quantification presented, comparing left and right hemisphere. Why are the two ROIs from left and right hemisphere from different hippocampal layers (contralateral includes SP, ipsilateral does not)?

Response: We have now increased the number of animals analyzed to a total of three and performed quantitative analysis comparing the ipsilateral and contralateral hemispheres. In addition, we have included representative images from both two hippocampal layers, one near the stratum pyramidale (SP) and another from the stratum radiatum (SR).

b. Secondly, I don't see why the construct is expressed throughout the brain (as evidenced by HA staining) but GFAP seems mostly visible in hippocampus, but not the overlaying cortex that should receive stronger light levels.

Response: The widespread HA staining reflects the use of the AAV-PHP.eB virus to achieve systemic expression (Supplementary Figure 8B). We chose this approach to ensure broad Opto-T1 expression throughout the brain, allowing us to test whether localized light stimulation is sufficient to induce astrocyte morphogenesis. Therefore, although Opto-T1 is expressed broadly, the morphological changes occur specifically in the hemisphere receiving light stimulation, demonstrating that both viral expression and light stimulation are required.

Regarding the relatively weak GFAP signal in the cortex, compared with the hippocampus, cortical astrocytes generally exhibit lower basal GFAP expression (*Jurga, Agnieszka M., et al. "Beyond*

the GFAP-astrocyte protein markers in the brain." Biomolecules 11.9 (2021): 1361.). Because the astrocyte morphogenesis is associated with GFAP (and other cytoskeletons) remodeling even in the absence of protein synthesis (as shown in *Figure 6*), the structural changes may occur more slowly in cortical regions with lower baseline GFAP levels. It is possible that different stimulation parameters (e.g., stronger or longer light stimulation) could eventually reveal cortical GFAP changes over a longer time scale. However, in the present study we focused primarily on the hippocampus, where GFAP expression and remodeling are more readily detectable.

c. What are the effective light levels inside the hippocampus with this method? This could be measured in a cadaver, for example. Rough estimates would be around 10-6, resulting in hippocampal irradiance of ~ 0.0005 µW/mm2.

Response: We are grateful for this helpful suggestion. We were also interested in estimating the degree of light attenuation reaching the hippocampus. To address this, we measured light transmission through mouse tissues *ex vivo*, inspired by the reviewer. Specifically, we isolated the scalp, skull, and cortical tissue (~1 mm) from mice and measured the transmitted light using the photodetector.

From these measurements, we observed an attenuation of approximately three orders of magnitude (~10^3 reduction). Although this estimate is about 1000-fold higher than the reviewer's rough estimate, it nevertheless confirms that a substantial fraction of the incident light is attenuated before reaching the hippocampus. For transparency, we have included these measurements in the Supplementary Figure 8c.

Despite this substantial attenuation, our functional data indicate that sufficient light likely reaches the hippocampus to activate Opto-T1. Astrocyte morphogenesis was observed only when both Opto-T1 expression and non-invasive blue light stimulation were present (Figure 8). Moreover, when the virus was expressed systemically but light stimulation was applied to only one hemisphere, astrocyte morphogenesis occurred exclusively on the illuminated hippocampus. These results suggest that, although the transmitted light is greatly reduced, the remaining light reaching the hippocampus under our stimulation conditions is sufficient to induce Opto-T1-dependent astrocyte morphogenesis.

d. Hong & Heo (2020) still used double the irradiance and three times the duration presented here.

Response: We thank the reviewer for the careful evaluation and for raising concerns regarding blue light condition. Although the actuator is different, another study has also demonstrated that blue light can reach and act within the hippocampus (see below). In that work, even a brief exposure of blue light at 1 mW/mm^2 for only 30 seconds was sufficient to activate FLP recombinase and induce FRT-STOP-FRT GFP expression in whole hippocampus, indicating that blue light is capable of reaching the hippocampus in mice.

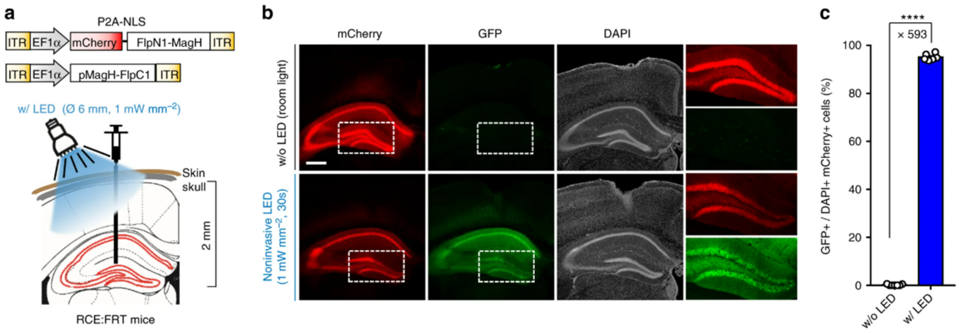

Jung, H., Kim, SW., Kim, M. et al. Noninvasive optical activation of Flp recombinase for genetic manipulation in deep mouse brain regions. Nat Commun 10, 314 (2019). https://doi.org/10.1038/s41467-018-08282-8

In addition, we believe that the optimal duration of light stimulation should depend on the specific brain region and cell type being targeted. In our study, we show that 10 minutes of stimulation in the hippocampus is sufficient to induce astrocyte morphogenesis, similar to BDNF treatment. Furthermore, in our synaptic plasticity experiments, 10 minutes of stimulation was sufficient to maintain LTP, mimicking BDNF application, and we also show that 10 minutes of stimulation delivered 12 hours after learning is sufficient to enhance memory persistence, as BDNF infusion did. Together, these results indicate that 10 minutes of stimulation is adequate to produce the observed effects.

As the reviewer also noted in the following question (e), longer stimulation could introduce potential confounding factors. For this reason, we are cautious about extending the stimulation duration beyond what is necessary to achieve the observed effects.

e. Is vision becoming impaired from such high levels of constant blue light exposure? Can mice still perform visual discrimination, for example? Zhang et al. (2024, https://doi.org/10.1016/j.exer.2024.109946) describe significant retinal damage in mice when exposed for 2h to 2.55 mW/cm2 (0.0255 mW/mm2) 450 nm light. This is one twentieth of the light levels used in this study.

Response: Thank you for raising this important point. We agree that strong blue light exposure can potentially cause unexpected/unwanted damage. In our experiments, mice were exposed to blue light for 10 minutes rather than the 2-hour exposure reported in Zhang et al. (2024), which may reduce the likelihood or severity of retinal damage. Furthermore, in Zhang et al. (2024), light was delivered directly to the eyes, whereas in our experiments the animals were not necessarily keeping their eyes open during illumination.

Importantly, mice in our experiments did not show impairments in recognizing a novel context, suggesting that gross behavioral performance was not affected under our conditions. In addition, both control and experimental groups were exposed to the same light stimulation protocol. Therefore, any potential visual impairment would be expected to affect both groups equally and is unlikely to account for the observed differences.

However, we thank the reviewer for raising this point, and for transparency we have added a note in the Discussion to acknowledge this potential limitation.

Revised manuscript

Supplementary figure 8

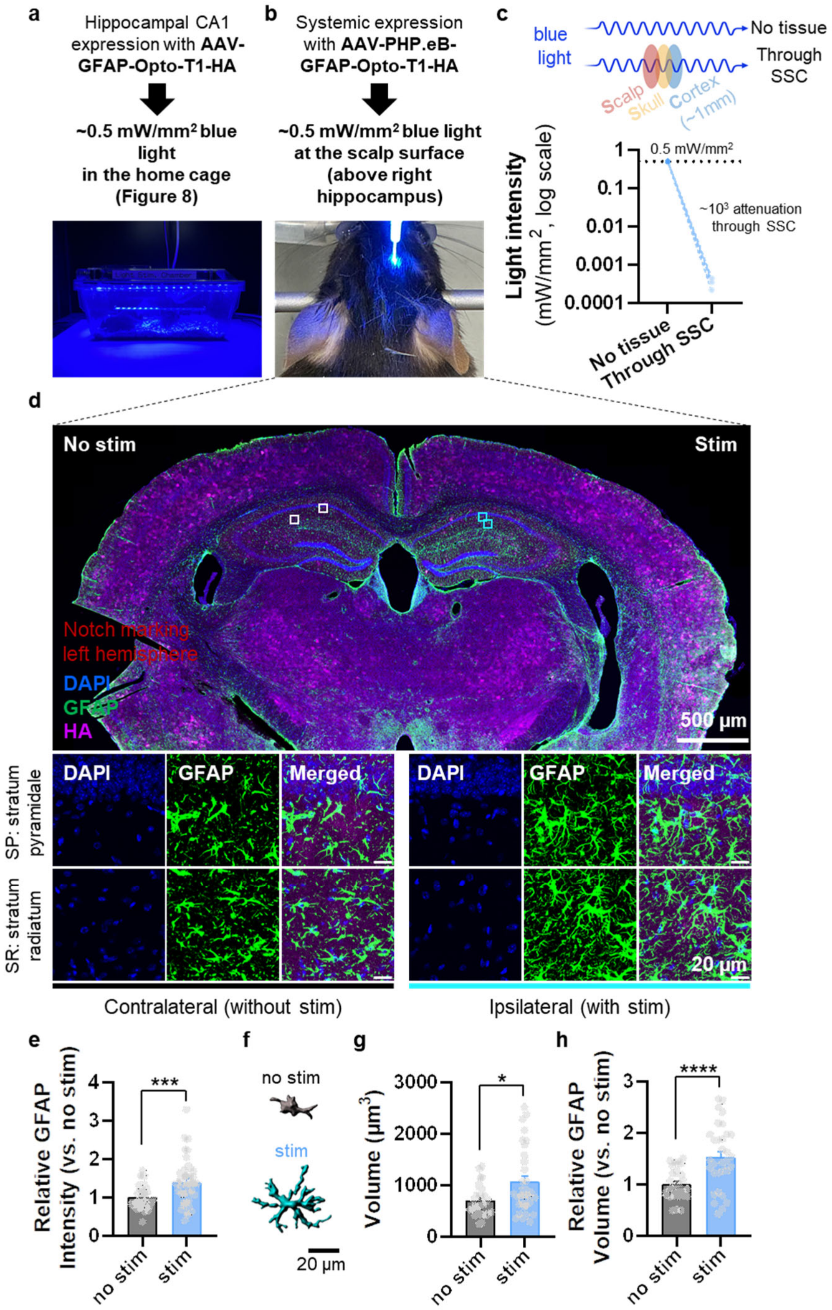

Supplementary Fig. 8. Non-invasive blue light stimulation (0.5 mW/mm²) is sufficient to activate Opto-T1 through the intact scalp, skull, and cortex (SSC). (a) Non-invasive blue light stimulation setup in the home cage (0.5 mW/mm² through a custom LED lid) used in Figure 8. (b-h) To directly test light penetration, Opto-T1 was broadly expressed via retro-orbital AAV-PHP.eB-GFAP-Opto-T1-HA. Three weeks later, the blue light (473 nm, 0.5 mW/mm², 10 min) was applied to the scalp above the right

hemisphere in anesthetized mice. The mice were sacrificed 1 h after stimulation (the left hemisphere was notched for identification after fixation). (c) Blue light transmission through overlying tissues was measured by placing cadaver-derived scalp, skull, and cortex (SSC) above the photodetector. Irradiance decreased by approximately three orders of magnitude (10^3) when light passed through SSC compared to the no-tissue condition. (d) GFAP immunostaining revealed robust activation only in the illuminated hemisphere, demonstrating that 0.5 mW/mm^2 blue light is sufficient to activate Opto-T1 through the intact scalp and reach hippocampal CA1. (e) Relative GFAP intensity normalized to the no stimulation (no stim) condition. n = 35 (no stim) and 44 (stim) cells; 7 slices from 3 mice. (f) Representative GFAP volume rendering using IMARIS. (g) GFAP volume. (h) Relative GFAP volume (normalized to no stim). n = 29 (no stim) and 32 (stim) cells; 7 slices from 3 mice. Data are mean ± SEM. **P < 0.01, ***P < 0.001, ****P < 0.0001. Mann-Whitney test (e, g) and unpaired t-test (h). Detailed statistics are provided in Table S1.

DISCUSSION

It should be noted that strong non-invasive blue light itself may influence baseline freezing levels[56] or **retinal damage[57]**, and therefore comparisons should be made between groups exposed to identical light conditions.

METHODS

Laser attenuation through scalp, skull and cortex (SSC) tissues

Laser power was measured using a PM100D optical power meter with an S121C photodiode sensor (Thorlabs, Newton, NJ, USA), under blue light illumination (0.5 mW/mm2). Cadaver-derived scalp, skull and cortex tissues (SSC) were placed over the detector during illumination to measure attenuation through overlying tissues, with the cortex approximately 1 mm thick.

Reviewer #3 (Remarks to the Author):

While contextual fear memories are known to depend on neurons in the hippocampal formation, much less is known about the role of astrocytes in the formation and maintenance of memories. Hayoung et al. have used a wide range of experimental methods to demonstrate that the cytoskeletal adapter protein Ank2 supports BDNF-mediated structural plasticity in astrocytes that is required and sufficient for remote memory expression.

While their work is technically of a high standard, encompasses a wide range of methods, and convincingly demonstrates links between Ank2 and the structural integrity of astrocytes, the links between Ank2 and BDNF as well as Ank2 and memory persistence currently appear weak and require further evidence to support the authors' claims.

If further evidence can be provided, the identification of a mechanistic link between cytoskeletal proteins in astrocytes supporting memory persistence in the hippocampus would be an important discovery to warrant publication in Nature Communications.

Response: We greatly appreciate the reviewer's thoughtful assessment of our study. We are encouraged by the recognition that our work is technically rigorous and that it highlights an important and underexplored role of astrocytic Ank2 in memory. At the same time, we acknowledge the reviewer's concerns regarding the strength of the evidence linking Ank2-IP3R2 in BDNF signaling and to memory persistence. We have carefully addressed these points through additional analyses and clarifications, which are detailed below in blue, with corresponding revisions to the manuscript indicated in red for clarity.

I appreciate the authors' efforts in addressing the points raised by us. Many points have now been addressed, but we feel that further additions to text, analysis, and additional experimental data are still required to support the claims of the manuscript. Most importantly, the feasibility of non-invasive blue light stimulation of the authors' Opto-T1 construct needs to be convincingly demonstrated.

Major:

1. The authors' main argument rests on their finding of impaired remote memory performance after conditional Ank2 deletion in CA1 astrocytes (Fig. 1m). Where is Fig. 1n on memory persistence that is referred to in the legend? Is persistence affected? If not, why not? Were these injections performed bilaterally or unilaterally? – the figure legend/methods remain unclear on this. Why were only 9/8 mice tested here? The statistically differences are significant but barely so. They are definitively much weaker than those shown in Fig. 1f, which begs the question if there are other astrocytic populations that play a more important role? The authors should strengthen their claims by either consolidating their results with additional animals or identify other targets outside CA1 that could explain the clear behavioural phenotype seen in Fig. 1f.

Response: We thank the reviewer for this valuable comment and for pointing out the issues in Fig. 1. In the revised manuscript, we have clarified that Fig. 1n represents memory persistence following bilateral injections of Cre virus into the CA1 of Ank2 floxed mice. The figure legend and Methods section have been corrected to clearly describe the injection strategy. We increased the number of animals included in this analysis and obtained consistent results, which strengthen and support our original conclusion.

We also acknowledge that the behavioral effect size observed in CA1-targeted deletion is smaller than that seen with broader astrocytic manipulations, suggesting that additional astrocytic populations outside CA1 may contribute to memory persistence. The most likely candidates are astrocytes in regions such as the medial prefrontal cortex (mPFC) or anterior cingulate cortex (ACC), which have been highly implicated in the storage of remote memories. While a systematic dissection of these regions is beyond the scope of the present study, we agree this represents an important avenue for future investigation, and we have noted this possibility in the revised Discussion.

Revised manuscript

Results

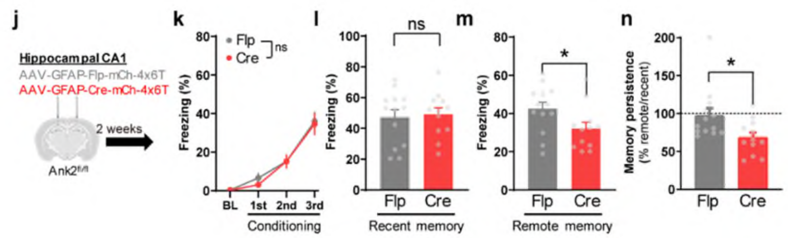

Fig. 1. Astrocytic Ank2 deletion impairs memory persistence. (j) Hippocampal astrocyte-specific Ank2 deletion. (k-n) Freezing behavior during acquisition (k), recent memory test (l), and remote memory test (m). (n) Memory persistence in mice with hippocampal astrocytic Ank2 deletion. Flp group, n = 13 mice; Cre group, n = 12 mice. Data are mean ± SEM. *P < 0.05, ***P < 0.001, ****P < 0.0001; ns, not significant. Two-way repeated measures ANOVA (d, h, k), unpaired t-test (e, f, l, m), Mann-Whitney test (g, n), and t-tests for (i): paired within groups, unpaired between groups. Detailed statistics are provided in Table S1.

Discussion [lines 469-479]

Beyond its role in memory persistence, Ank2 may have broader functions in astrocyte biology, including the regulation of astrocyte maturation. While GFAP-CreER^{T2} was selected in this study to reduce peripheral recombination, ALDH1l1-CreER^{T2} exhibits pan-astrocytic expression and targets a broader range of astrocyte subtypes, which could reveal additional roles of Ank2 in astrocyte populations not efficiently targeted by GFAP-based approaches. Notably, astrocyte-specific ARL13B deletion using ALDH1l1-CreER^{T2}, which disrupts primary cilia, leads to reduced Ank2 expression and a decrease in astrocytic surface area[60]. Given the link between astrocytic structural integrity and neurodevelopmental disorders such as ASD, further investigation is warranted to determine how Ank2 contributes to astrocyte maturation and whether it influences brain development. Whether Ank2 primarily regulates astrocytic morphology and signaling or also contributes to broader neurodevelopmental processes remains an open question, with potential implications for ASD and intellectual disability.

The authors should clarify if bilateral or unilateral injections were used for Fig. 1j-n. Fig. 1j suggests bilateral, but I cannot find references to this in methods/results. The authors are mentioning in their response a possible role of Ank2 in ACC or mPFC to explain the relatively weak CA1-specific deletion, but the suggested discussion points evade this topic. The authors should point out in their discussion the relatively lower levels with reference to other brain regions that may be involved.

2. The relationship of BDNF with Ank2 appears rather weak and requires further data. For example, while the authors suggest an impaired "volume change (Δ Volume)" in cKO animals after BDNF treatment (Fig. 6d-e), it is unclear why the authors would quantify absolute differences and not proportional differences. Indeed, comparing Fig. 6c and 6d, it appears that booth ctrl and cKO volumes approximately double in volume after BDNF treatment, which suggests that cKO astrocytes are still responding the same way to BDNF as controls, despite their lower baseline. Furthermore, in Fig. 7, the authors claim that "BDNF-induced memory persistence is reduced in astrocyte-specific Ank2-deleted mice", but the authors only compare control+vehicle, control+BDNF, and cKO+BDNF, but not the criticial cKO+vehicle. Hence, it remains unclear what the baseline cKO level is that any BDNF effects would need to be compared to.

Response: We thank the reviewer for this insightful comment. We agree that both control and Ank2 cKO astrocytes show approximately a two-fold increase in volume following BDNF treatment. However, we chose to quantify absolute volume rather than proportional change because the ability of astrocytes to contact and support surrounding synapses is ultimately constrained by their absolute size. Thus, although the relative increase appeared similar, Ank2 cKO astrocytes began from a lower baseline and remained significantly smaller even after BDNF treatment, which likely limits their functional capacity to support synaptic interactions.

I disagree with the authors' interpretation that their data in Fig. 6c-e demonstrate "impaired BDNF-dependent astrocyte morphogenesis". To claim this, the authors would need to run a 2-way ANOVA that specifically tests for interaction effects of BDNF and cKO. Judging from these panels, I would guess that the ANOVA would reveal a significant main effect for both BDNF as well as the knockout condition, but might fail to reveal interaction effects. This would be interpreted as BDNF increasing GFAP volume and cKO animals showing less volume. Only an interaction effect would demonstrate that BDNF-dependent increases in GFAP volume are impaired in the cKO condition, not the currently displayed two Mann-Whitney tests and a bar plot in (e) without statistics.

Regarding the cKO+vehicle condition, our behavioral experiments were specifically designed to test whether exogenous BDNF supports memory persistence and whether this effect requires astrocytic Ank2. Mild contextual fear conditioning does not induce remote memory persistence, as previously reported (DOI: 10.1007/s12035-018-1463-4). Consistent with this, and given that we had already observed that Ank2 cKO mice fail to form remote memory persistence even under stronger training conditions, we did not include a separate cKO+vehicle group, as this condition was not expected to yield remote memory either. Instead, we focused on comparing the effect of BDNF infusion across genotypes. First, in control mice, hippocampal BDNF infusion delivered 12 hours after training significantly enhanced memory persistence, indicating that exogenous BDNF is sufficient to induce memory persistence. Next, we asked whether this BDNF-dependent effect is preserved in Ank2 cKO mice. We found that, despite receiving identical BDNF treatment (with genotype being the only difference), Ank2 cKO mice exhibited markedly reduced memory persistence. This divergence indicates that astrocytic Ank2 is required for the BDNF-dependent memory persistence.

Again, I strongly disagree with the authors' interpretation of the data and their statement that "we did not include a separate cKO+vehicle group, as this condition was not expected to yield remote memory either". To test whether BDNF-induced memory is significantly affected in the cKO condition, the authors would need to show an interaction effect in a two-way ANOVA with factors ctrl/cKo and Veh/BDNF. To show this, the control of cKO+vehicle is thus critical. The two "Ctrl" conditions demonstrate that BDNF usually leads to an approximate 1.5x increase in memory persistence, but to show

that this BDNF-dependent memory persistence increase fails in cKO mice, we need to know the vehicle memory persistence levels. This data is missing. If, as the authors suggest in their response, no memory persistence was to be expected, say a level of perhaps 10%, then the cKO+BDNF value might still represent an increase, perhaps also of 1.5x. Otherwise, it remains simply unknown if cKO animals simply have bad memory persistence per se or whether this memory persistence deficit is BDNF-dependent. Hence, such a condition and test are crucial to the authors' main hypothesis.

3. I remain unconvinced of the efficacy of the Opto-T1 construct. Since the authors newly developed this construct and show only a single quantitative panel featuring an unknown number of mice, further (in vitro) data is required to establish this tool as functional. I am furthermore surprised to see this construct improving memory persistence. Why is the memory persistence of controls in Fig. 1g >90%, while it is ~60% in Fig. 8i? Is this due to different shock protocols? If yes, the authors should also show the data with congruent shock protocols (3 shocks in Fig. 8; 1 shock in Fig. 1). Furthermore, to demonstrate the currently missing link to Ank2, the authors should aim to rescue the memory phenotype produced by Ank2-GFAP-cKO with the activation of opto-T1 in the hippocampus.

Response: We thank the reviewer for raising these important points. To strengthen the validity of our Opto-T1 construct, we performed additional experiments showing that its activation produces effects similar to BDNF treatment in sustaining long-term potentiation in the hippocampus, supporting the conclusion that Opto-T1 in astrocytes is functional. We also note that, since the seminal pioneering work of Rose et al. (2003, Nature), relatively little progress has been made in delineating the downstream signaling pathways of TrkB.T1 in astrocytes. We therefore view Opto-T1 as a useful new tool to pursue such mechanistic questions in future studies, and we are planning more detailed investigations of this tool and the downstream mechanisms of TrkB.T1.

In the present study, we included the Opto-T1 experiments for a specific reason. The Ank2 coding sequence is exceptionally large (~6 kbp), which made it technically unfeasible to restore Ank2 expression in an astrocyte-specific manner using our AAV virus system. As an alternative strategy to assess astrocytic sufficiency, we modified the well-established Opto-cytTrkB construct (Woo et al., 2019, Cell Chemical Biology, Hong and Heo, 2020, Journal of Molecular Biology) by replacing the full-length TrkB domain with TrkB.T1. Aside from this domain difference, the construct is identical to the original tool, and indeed it mimicked BDNF-dependent astrocyte morphogenesis. This approach allowed us to test directly whether selective activation of astrocytic signaling is sufficient to support memory persistence, thereby complementing our loss-of-function experiments and underscoring the contribution of astrocytes.

A notable aspect of this tool is that it also increased GFAP expression, yet without inducing the detrimental features of reactive astrocytes that are typically associated with impaired LTP and memory. Instead, similar to BDNF treatment, astrocytic activation of Opto-T1 enhanced LTP maintenance and remote memory persistence, indicating that GFAP elevation in this context reflects functional astrocytic engagement rather than pathological reactivity. We believe this unique feature adds further significance to the Opto-T1 findings in the context of astrocytic roles in memory persistence.

Regarding the difference in memory persistence levels between Fig. 1 and Fig. 8, this was intentional. We used a weaker conditioning paradigm that results in minimal induction of BDNF expression around 12 hours after training and lower memory persistence, making this time window particularly suitable for testing whether exogenous BDNF (Fig. 7) or Opto-T1 activation (Fig. 8) can enhance memory persistence. Based on the existing literature and previous studies, and with approval

from IACUC, we designed the experiments to specifically address three questions: (1) whether Ank2 is required to maintain memory persistence under strong conditioning, (2) whether exogenous BDNF can sustain memory persistence under mild conditioning where it normally fails, and (3) whether Ank2 is necessary for BDNF-induced memory persistence. Therefore, we focused experiments (Fig 7 and 8) on conditions where endogenous BDNF induction is minimal, rather than on strong conditioning in which BDNF is already robustly induced.

Finally, we sincerely thank the reviewer for suggesting the possibility of a rescue experiment in Ank2 cKO mice using Opto-T1, which we found truly inspiring. However, because the expression and functions of IP3R2 are already reduced in Ank2 cKO astrocytes, activating upstream signaling through Opto-T1 would likely be ineffective in resolving the deficits observed. However, the reviewer's comment has inspired us to consider exploring IP3R2 downstream signaling and to investigate whether these pathways can be modulated by optogenetic approaches. Such experiments, particularly those that drive astrocytic morphogenesis, would represent a powerful and informative direction, and we are very grateful for this suggestion. Given the scope of the current study, we felt it most appropriate to conclude our experiments here, while highlighting this as an exciting avenue for future work.

I thank the authors for their clarification and the additional LTP experiments. It remains unfortunate that the final piece of evidence is missing any link to Ank-2, but I agree that further experiments along these lines would likely go beyond the current scope.

4. The authors need to explain the link between the visible decline of Ank2GFAP-cKO in L-LTP starting at 2 hours after LTP induction (Fig. 4b) and their behavioural results indicating no memory deficits 24 hours after conditioning (Fig. 1e). How do the authors reconcile the fact that LTP seems impaired already after 2 hours, while behavioural phenotypes remain intact for at least 24 hours? The authors need to discuss this point or produce further evidence to reconcile these results.

Response: We thank the reviewer for this important comment. We acknowledge that our *ex vivo* LTP recordings and the behavioral results occur on different timescales. While LTP has long been considered a cellular correlate of memory storage, actual learning and memory formation involve multiple layers of consolidation, including synaptic, circuit, and systems-level processes, which take hours to days. Thus, the impairment in L-LTP maintenance observed within a 3-hour recording window should not be directly equated to a strict time limit for memory retention *in vivo*, particularly in the context of *ex vivo* slices. Although we confirmed in additional experiments that hippocampal slices can remain healthy for more than 3 hours (Fig. 4), *in vivo* conditions involve systemic consolidation processes across multiple brain regions and the action of various neuromodulators, which are not fully captured in slice preparations. Therefore, the LTP impairment we observed likely reflects only part of the overall process, which may explain the apparent temporal discrepancy. We have clarified this point in the revised manuscript.

Revised manuscript

Discussion [lines 459-465]

Finally, while ex vivo recordings revealed impaired LTP maintenance in Ank2 cKO mice, this finding should be interpreted with caution. Although LTP has long been regarded as a cellular correlate of memory storage, actual learning and memory formation involve multiple levels of consolidation, from

synaptic to systems-level processes. Therefore, impairment of L-LTP over 3 hours cannot be directly equated to a 3-hour limit in memory retention. Nevertheless, the data indicate a reduced ability to maintain synaptic potentiation, and it is possible that diminished astrocytic metabolic support in Ank2 cKO mice limits the capacity to sustain potentiated synapses.

5. Why was eGRASP used in mice that underwent conditioning and memory tests if no behavioural results are shown (Fig. 4c-g)? The authors should show if memory expression levels are correlated with eGRASP density, as would be expected. If not, the authors need to explain this or adjust their model.

Response: We thank the reviewer for this thoughtful comment. As clearly described in the figure legend, main text, and Methods, the memory test was performed one day after training, and brains were immediately fixed thereafter for astrocyte-eGRASP analysis. We have now included the corresponding behavioral results below.

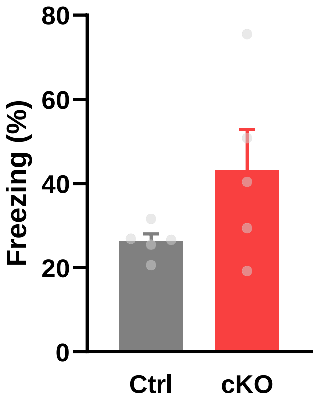

Although this experiment required additional manipulations (habituation to the anesthesia chamber, inhalation anesthesia, and doxycycline injection prior to learning), the results were consistent with those shown in Fig. 1. Ank2 cKO mice did not exhibit a deficit in recent memory. The cKO group even showed slightly higher freezing, which was largely driven by one mouse with high freezing levels. Importantly, despite this behavioral outcome, astrocyte-eGRASP density was clearly reduced in Ank2 cKO mice, indicating that synaptic contact reduction can be detected independently of behavioral performance at recent time points.

We couldn't test the astrocyte-eGRASP in the remote timepoint because astrocyte-eGRASP is a novel tool (Kim et al., 2023), and its long-term stability has not yet been fully established. In particular, because our design used a doxycycline-based labeling strategy, both the stability of eGRASP reconstitution and the persistence of engram neuron labeling decline over time. For this reason, we focused our analysis on Day 1 after conditioning, when labeling is still reliable.

Importantly, this limitation also led to an unexpected finding. Even though behavioral recall on Day 1 did not differ between groups (Fig. 1 and above), astrocyte-eGRASP revealed a clear reduction in contacts onto engram neurons in Ank2 cKO mice at this recent time point. This indicates that astrocytic contacts are already altered one day after learning, before behavioral differences emerge (i.e., remote time point). Notably, this occurred on the day after the critical 12-hour window in which BDNF signaling is required for memory persistence, highlighting the significance of this observation. Further studies, for

example using longitudinal *in vivo* imaging of astrocyte morphology and astrocytic contacts on engram neurons, will be needed to determine how astrocytic structure dynamically changes after learning (recent versus remote time points). Our results suggest that these early structural alterations may provide a mechanistic basis for later differences in spine stability and memory persistence.

I thank the authors for clarifying their data but suggest they also offer these explanations to the readers of the manuscript. That is, the freezing data should find its way to the supplementary and the implications, as described in the authors' response in the previous paragraph, should find its way into the discussion.

6. The authors claim "Ank2 as a key regulator of IP3R2 function" (l. 269-270), referring only to one panel of co-immunoprecipitation (Fig. 5a) and a single article published in 2022 in Molecular Psychiatry. The data in Fig. 5b-h shows an impairment in calcium dynamics that could be the result of other cellular processes. The authors should either tone down this statement or find further evidence for this.

Response: We thank the reviewer for this valuable comment. We acknowledge that our initial wording "Ank2 as a key regulator of IP3R2 function" was overstated given the limited evidence originally presented. To strengthen this point, we performed additional analyses and experiments. First, STRING analysis of Ank2 molecular functions revealed the strongest enrichment for inositol 1,4,5-triphosphate-sensitive calcium-release channel activity, suggesting a functional link to IP3Rs. Second, to directly test whether the Ank2-IP3R2 interaction is required for astrocytic morphogenesis, we expressed a dominant-negative construct containing the ankyrin-binding domain (ABD) of IP3R2 in astrocytes. While this manipulation had little effect under basal conditions, it significantly impaired BDNF-dependent astrocytic morphogenesis, indicating that Ank2-IP3R2 binding contributes to astrocytic BDNF signaling.

Based on these results, we have revised the manuscript to more cautiously state that Ank2 supports IP3R2 stability and function, rather than serving as a "key regulator." We have also noted in the Discussion that other cellular processes may contribute to the impaired calcium dynamics in Ank2 cKO.

Revised manuscript

Results [lines 228-233]

Given that IP3R2 is the predominant isoform in astrocytes, we hypothesized that Ank2 supports IP3R2 function in mediating BDNF signaling. STRING analysis of Ank2 molecular function revealed strong enrichment for inositol 1,4,5-triphosphate-sensitive calcium-release channel activity and high association scores with cytoskeletal proteins (Fig. 5a), suggesting a functional link to IP3Rs. Co-immunoprecipitation from brain lysates confirmed Ank2 binding to IP3R1, as previously reported[43], and additionally to IP3R2, with minimal binding to IP3R3 (Fig. 5b). IP3R2 protein levels were significantly reduced in hippocampal astrocytes of Ank2 cKO mice, indicating that Ank2 contributes to the expression of IP3R2 (Supplementary Fig. 6a, b).

Results [lines 248-258]

To specifically interrogate the functional relevance of the Ank2-IP3R2 interaction, we targeted the ankyrin-binding domain (ABD) located in residues 955-991, which is conserved among IP3Rs[46]. We expressed the IP3R2 ABD as a dominant-negative construct (AAV-GFAP-eGFP-P2A-IP3R2 ABD) in astrocytes (Fig. 5i-l, Supplementary Fig. 7a, b). Expression of IP3R2 ABD alone did not alter GFAP

intensity (Supplementary Fig. 7a, b). We next asked whether expression of IP3R2 ABD alters BDNF-dependent astrocyte morphogenesis *in vivo*. Hippocampal infusion of BDNF (100 ng/0.4 μL) induced astrocyte morphogenesis (Supplementary Fig. 7c-e). Astrocytes expressing IP3R2 ABD exhibited significantly lower GFAP volume after BDNF infusion compared with controls (Fig. 5k, l).

Together, these results demonstrate that Ank2 supports IP3R2 function to mediate BDNF-dependent astrocyte morphogenesis. Given the established role of IP3R2 in L-LTP and remote memory[2], Ank2 emerges as a key regulator of astrocytic structural plasticity that underlies memory persistence.

Discussion [lines 448-451]

It should be acknowledged that the present study focused primarily on the BDNF-IP3R2 pathway, yet other mechanisms could also contribute to the observed phenotypes. Exploring these alternative possibilities will be necessary to fully understand the roles of Ank2 in astrocytic Ca^{2+} signaling and neuron-astrocyte interactions.

I appreciate the authors' additional work on establishing the links between Ank2 and IP3R2. As before, however, to show BDNF-dependent deficits on GFAP volume in these knockouts, the authors need to perform two-way ANOVAs that contain main effects for BDNF/vehicle as well as Ctrl/IP3R2 ABD, and test for interaction effects. This is necessary to establish that the BDNF-derived volume increases are not seen in IP3R2 ABD mice, and not just a matter of reduced baseline GFAP volume levels.

Minor points:

• The author should consider changing the title, which is more specific to the fear memory persistence. For example, memory can be classified as aversive and pleasant memories, explicit and implicit memories, or long-term and short-term memories. If the authors give the paper a title, "Astrocytic Ankyrin2 Enables Memory Persistence", the experiments must be designed to investigate various types of memories; alternatively, the authors may want to be more specific in their title.

Response: We thank the reviewer for this important comment regarding the title. We also considered whether astrocytic Ank2 might be specific to fear-related memory or extend to other forms of memory. While it is not feasible to examine every type of memory within this study, we performed an additional object location memory test in Ank2 cKO mice. Although object location memory is typically retained for only ~2 days and thus is not generally categorized as remote memory, it is regarded as a hippocampus-dependent form of long-term memory. We found that Ank2 cKO mice failed to discriminate the changed object location, unlike controls, indicating that the role of astrocytic Ank2 is not restricted to fear memory but extends to other hippocampus-dependent long-term memories. Based on these findings, and in order to specify the scope of our work more precisely, we have revised the title to highlight the hippocampal context of our study.

Revised manuscript

Title: Astrocytic Ankyrin2 Enables Memory Persistence in the Hippocampus

• In Fig. 7e, the authors show context specificity for the memory tested. The authors should show such specificity also for the data presented in Fig. 1 to rule out indiscriminate anxiety.

Response: In Figures 7 and 8, we specifically tested context specificity in order to evaluate whether BDNF- or astrocyte-mediated enhancement of memory persistence was context-dependent. In contrast, the experiment in Figure 1 was designed simply to assess whether memory for the shock-associated context itself was reduced, and therefore an alternative context was not included in this paradigm. Although we did not perform a context-specificity test in Figure 1, we and others have repeatedly demonstrated context-specific learning in contextual fear conditioning using naïve C57BL/6 mice, and we therefore expect that freezing levels in a novel context would have remained low, as no associative memory would have been formed.

• The Cre-LoxP system is a widely used tool for genetic modification in mouse models. However, the specificity of the virus needs to be quantified and shown in the paper. For example, the percentage of colocalization between astrocytes and the viral marker. Will hippocampus-specific deletion of astrocytic Ank2 decrease the number or volume of astrocytes? The images in Fig. 1.j are not enough to show the specificity. Please give the calculation results and add a higher magnification of the colocalized examples.

Response: We thank the reviewer for raising this important point. Although the Cre virus we used has been validated in a previous study (DOI: 10.1038/s41467-023-42746-w), we re-confirmed its specificity in our experiments using immunohistochemistry and added these results to Supplementary Figure 3. We also realized that we had not previously noted the presence of a nuclear localization signal (NLS) in this construct and have now clarified this in the manuscript. Because Cre contains the NLS, we performed colocalization analysis with Sox9 as an astrocyte marker and NeuN as a neuronal marker. The results confirmed astrocyte-specific expression of Cre. As suggested, we have updated the manuscript to include these findings, with higher-magnification images as well.

Revised manuscript

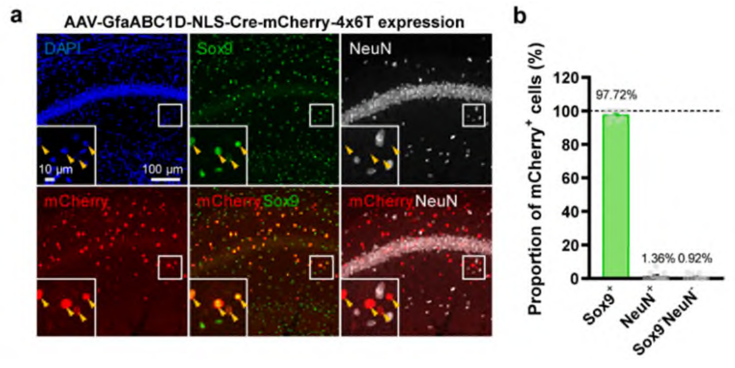

Supplementary Fig. 3. Specificity of AAV-GfaABC1D-NLS-Cre-mCherry-4x6T virus and quantification of Ank2 and Ezrin proteins as well as astrocyte number in the hippocampus. (a) Representative images of AAV-GfaABC1D-Cre-mCh-4x6T expression. Nuclei are stained with DAPI (blue), Cre-mCherry (red), astrocytes with Sox9 (green), and neurons with NeuN

(gray). Yellow arrowheads indicate mCherry localization in Sox9-positive ($Sox9^+$) cells. (b) Quantification of $mCherry^+$ cells from 11 images across 4 mice. Dots indicate the proportion of $mCherry^+$ cells (%) in each image. Of 630 $mCherry^+$ cells, 618 were $Sox9^+$, 6 were $NeuN^+$, and 6 were negative for both Sox9 and NeuN.

• The authors did not describe the experimental steps for contextual fear conditioning in the hippocampal BDNF diffusion groups.

Response: We have now clarified this in Main text as well as the Methods section.

Revised manuscript

Results [lines 75-82]

Next, we evaluated learning and memory using contextual fear conditioning (Fig. 1c-g). During the acquisition phase, both control and $Ank2^{GFAP}$ cKO mice exhibited similar responses to three electric shocks (Fig. 1d), indicating normal shock perception and immediate response. One day post-conditioning, memory recall (recent memory) showed no significant difference between groups (Fig. 1e). However, when remote memory recall was assessed 14 days post-conditioning, $Ank2^{GFAP}$ cKO mice exhibited significantly reduced freezing behavior compared to controls (Fig. 1f), suggesting impaired remote memory. Memory persistence, calculated as the ratio of freezing on day 14 to freezing on day 1, was also significantly reduced in $Ank2^{GFAP}$ cKO mice (Fig. 1g).

Methods [lines 508-534]

For both the recent and remote memory tests, a hexahedral conditioning chamber (18 cm wide × 18 cm long × 30 cm height; H10-11M-TC; Coulbourn Instruments) was used. The chamber consisted of a metal grid floor, aluminum sidewalls, and a clear plexiglass front door and back wall. In the conditioning protocol, after an acclimation period of 208 seconds, mice were exposed to a strong unconditioned stimulus consisting of three foot-shocks (0.75 mA, 2 s each) delivered at 30-second intervals. Following the final foot-shock, the mice remained in the conditioning chamber for an additional 30 seconds before being returned to their home cages. For the recent memory test, conducted 24 hours post-conditioning, the mice were placed in the conditioned context chamber for 5 minutes, during which freezing behavior was measured. For the remote memory test, conducted 14 days post-conditioning, the same procedure was repeated. Freezing behavior was analyzed using Freezeframe software, and memory persistence was assessed by calculating the ratio of freezing time on day 14 to day 1.

For the extinction test, the protocol consisted of 4 days. On the first day, after an acclimation period of 208 seconds, mice were exposed to three foot-shocks (0.75 mA, 2 s each) delivered at 30-second intervals. On the following day, mice were placed back into the conditioned context chamber for 30 minutes. Freezing behavior was measured every 5 minutes, resulting in 6 blocks. The same procedure was repeated on the third day. On the final day, freezing behavior was measured for 5 minutes and compared to the first block on the second day to calculate memory extinction.

For remote memory enhancement with BDNF infusion or Opto-T1, we used a mild conditioning protocol (single foot-shock, 0.75 mA, 2 s) following a 208-s acclimation period, as previously described[48]. Mice remained in the chamber for 30 s after the shock before returning to their home cages. Twelve hours after

acquisition, either BDNF (100 ng in 0.4 µl per side, delivered bilaterally at 0.2 µl/min) or optogenetic stimulation was applied. Detailed surgical procedures for BDNF infusion are described in Cannula implantation and infusion of BDNF in the hippocampus. Non-invasive blue light stimulation (~0.5 mW/mm^2 for 10 min) was delivered using a custom blue-light LED cage lid (Scitech Korea), with minor modifications from a previously described design[49]. Recent and remote memory were assessed 24 h and 14 days after conditioning, respectively, by measuring freezing for 5 min in the conditioned context. To test context specificity, mice were exposed to a novel cylindrical arena (20 cm diameter, 25 cm height) with bedding on the floor for 5 min one day after the remote memory test.

• In Fig.7 and Fig. 8.f-j, the authors used a similar protocol to generate fear memory in mice. However, the freezing time for the control groups is different (BDNF control group: ~27%, optic control group: ~50%) in the 1 day after conditioning contextual tests. The authors should discuss the potential reason in the discussion part or explain it with references.

Response: We appreciate the reviewer 3's insightful comment. As noted, the baseline freezing levels in the control groups differed between Figure 7 (BDNF experiment) and Figure 8 (Opto-T1 experiment). The major procedural difference is that, 12 hours after learning, BDNF was infused in Figure 7, whereas in Figure 8 mice in the home cage received non-invasive blue light stimulation. A potential reason is that blue light can be perceived by the mouse, and previous study has reported that blue light exposure may induce mild anxiety-like responses (10.1016/j.neuroimage.2025.1212). Such effects could contribute to the elevated baseline freezing in the optic experimental groups in Figure 8. Importantly, however, both the control and Opto-T1 groups in Figure 8 were exposed to identical blue light conditions, and no differences were observed at day 1. The divergence appeared only at day 14, strongly supporting our main conclusion that astrocyte-specific Opto-T1 activation influences memory persistence, despite the apparent difference in basal responses compared with the BDNF infusion experiments.

I appreciate the authors' discussion points and would like to suggest the authors share these with other readers in the manuscript's discussion section, as originally suggested.

• The authors need to list all statistical results for every figure. For example, the author should apply a suitable statistical analysis between groups (e.g., two-way ANOVA) for Fig. 1.d, h, and k, Fig. 2.d, and Supple. 5a.

Response: We thank the reviewer for this comment. All statistical results, including the appropriate analyses between groups, were provided in Table S1. We also recognize that readers may not always refer to supplementary tables. To improve clarity, we have now included the relevant statistical details directly in the figure legends, within the journal's 350-word limit per legend.

• The authors should provide an overview image of injection and implantation sites in the mouse brain for each mouse.

Response: We have now added additional images of the injection and implantation sites where they were missing, together with the detailed descriptions of the virus injection and implantation protocols.

Revised manuscript

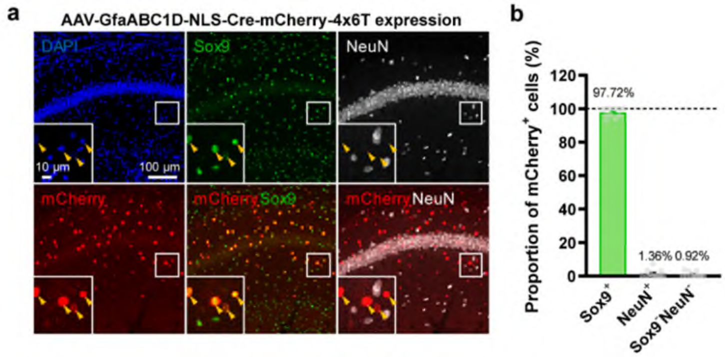

Supplementary Fig. 3. Specificity of AAV-GfaABC1D-NLS-Cre-mCherry-4x6T virus and quantification of Ank2 and Ezrin proteins as well as astrocyte number in the hippocampus. (a) Representative images of AAV-GfaABC1D-Cre-mCh-4x6T expression. Nuclei are stained with DAPI (blue), Cre-mCherry (red), astrocytes with Sox9 (green), and neurons with NeuN (gray). Yellow arrowheads indicate mCherry localization in Sox9-positive ($Sox9^+$) cells. (b) Quantification of $mCherry^+$ cells from 11 images across 4 mice. Dots indicate the proportion of $mCherry^+$ cells (%) in each image. Of 630 $mCherry^+$ cells, 618 were $Sox9^+$, 6 were $NeuN^+$, and 6 were negative for both Sox9 and NeuN.

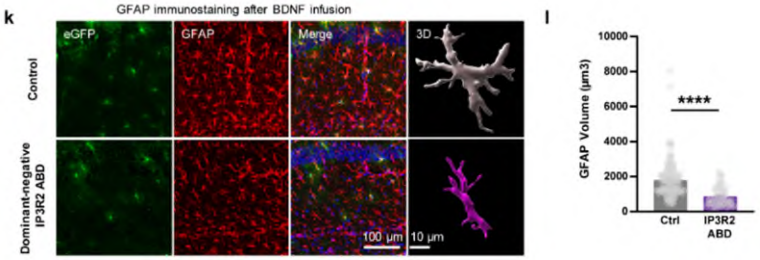

Fig. 5. Ank2 interacts with IP3R2 and regulates astrocytic Ca2+ signaling and BDNF-dependent morphogenesis. (k) Representative images of GFAP immunostaining and eGFP signals in hippocampal tissue following BDNF infusion (100 ng/0.4 μL). (l) Quantification of GFAP volume in astrocytes expressing control or IP3R2 ABD constructs after BDNF infusion. Control group, n = 167 astrocytes from 3 mice; IP3R2 ABD group, n = 75 astrocytes from 3 mice. Data are mean ± SEM. *$P < 0.05$, **$P < 0.01$, ****$P < 0.0001$; ns, not significant. Mann-Whitney test (e-h) and Welch's t-test (l). Detailed statistics are provided in Table S1.

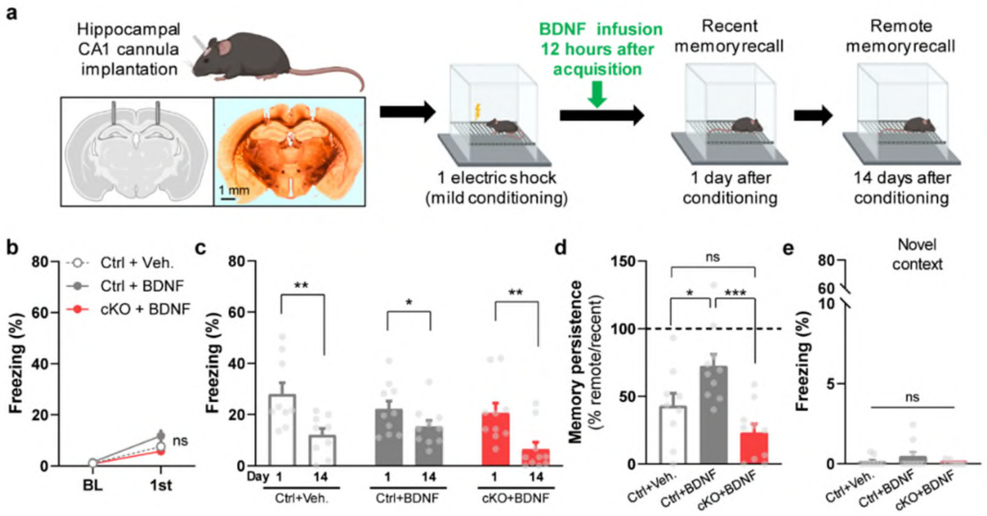

Fig. 7. BDNF-induced memory persistence is reduced in astrocyte-specific Ank2-deleted mice. (a) Schematic diagram of contextual fear conditioning with BDNF infusion into the hippocampal CA1. Mild conditioning (one electric shock instead of three) was used to assess BDNF-induced memory persistence. (b-e) Freezing behavior measured across different phases: (b) during acquisition, (c) in the recent and remote memory test. (d) Memory persistence in control mice with vehicle (aCSF) or BDNF (100ng/0.4μL/side) infusion, and in Ank2^{GFAP} cKO mice with BDNF infusion into hippocampal CA1 12 hours after memory acquisition. (e) Freezing behavior in the novel context test. Control+vehicle group, n = 9 mice; Control+BDNF group, n = 10 mice; Ank2^{GFAP} cKO+BDNF group, n = 10 mice. Data are mean ± SEM. *P < 0.05, **P < 0.01, ***P < 0.001. ns, not significant. Two-way repeated measures ANOVA (b), paired t-test (c, control+vehecle and control+BDNF), Wilcoxon test (c, Ank2 cKO+BDNF), Tukey's multiple comparisons test (d), and Dunn's multiple comparisons test (e). Detailed statistics are provided in Table S1.

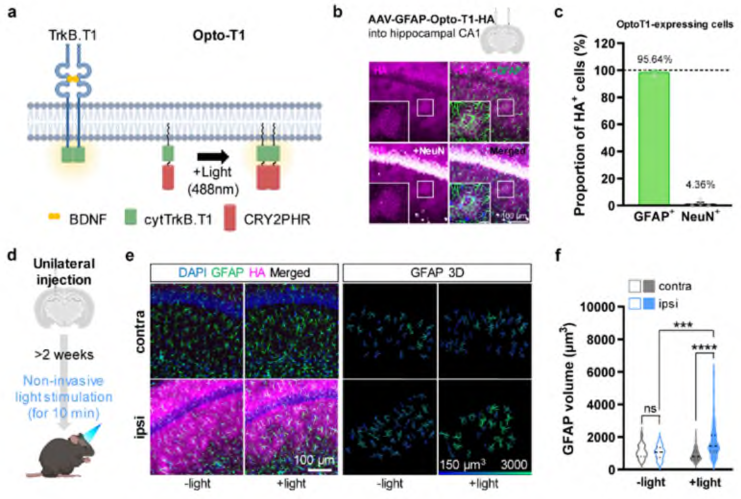

Fig. 8. Astrocyte-specific BDNF signaling sufficiently induces memory persistence. (a) Design of Opto-cytTrkB.T1 (Opto-T1), containing a light-sensitive CRY2PHR^{E281A} domain fused to the cytosolic domain of the TrkB.T1 receptor. (b) Representative image showing astrocyte-specific expression of AAV-GFAP-Opto-T1-HA. (c) Quantification of astrocytic specificity of Opto-T1 expression. Each dot indicates one slice. A total of 311 HA^{+} cells were analyzed from 4 slices across 3 mice. (d) Experimental scheme for unilateral virus injection and non-invasive continuous blue light stimulation (0.5 mW/mm^{2} in the home cage). (e) Representative GFAP and HA immunostaining of contralateral (non-injected) and ipsilateral (Opto-T1-injected) hippocampal CA1. (f) Comparison of GFAP volume without (-light, n = 3 mice; Contra, 32 astrocytes; Ipsi, 123 astrocytes) or with (+light, n = 3 mice; Contra, 49 astrocytes; Ipsi, 88 astrocytes) light stimulation

• What does the abbreviation “> 14 DPI” mean in Fig. 8.b and Supplementary Fig. 6.a?

Response: We have used DPI is an acronym for Days Post-Injection. But, to prevent confusion, we have changed the term from “>14 DPI” to “> 2 weeks”.

• The author should describe the laser delivery strategy in the methods part. How long is the laser stimulus? Is that pulsed or continuous stimulation? What is the wavelength used for the blue light?

Response: We have now clarified the laser delivery strategy in the Main text and Methods section.

Revised manuscript

Results [lines 356-357]

For non-invasive activation of Opto-T1, we placed a customized LED lid over the home cage, which delivered continuous blue light stimulation (~0.5 mW/mm^2, 10 min) to freely moving mice.

Results [lines 365-368]

In hippocampal slices from Opto-T1-expressing mice, continuous blue light stimulation (50 μW/mm^2, 10 min, 1 h post-3×TBS) likewise maintained LTP for at least 3 h (Fig. 8g, h), indicating that astrocytic TrkB.T1 activation is sufficient to sustain LTP maintenance.

Methods [lines 529-531]

Non-invasive blue light stimulation (~0.5 mW/mm^2 for 10 min) was delivered using a custom blue-light LED cage lid (Scitech Korea), with minor modifications from a previously described design[49].

Methods [lines 663-664]

To activate Opto-T1 expressed in astrocytes, blue light (473 nm; 50 μW/mm^2, MBL-III-473 nm, CNI Laser) was delivered for 10 minutes starting 1 hour after 3×TBS stimulation.

I have only now become aware that the authors are indeed using “non-invasive blue light stimulation” through – presumably – scalp, skull, cortex, to reach the Opto-T1 construct in hippocampal area CA1 while animals are freely moving in a home cage with an LED lid. I fail to fathom how the authors can reach sufficient intensities on the level of the hippocampus with such non-invasive stimulation, as I presume 0.5 mW/mm2 refers to the levels of light on the head, but not inside the hippocampus. The authors need to credibly show that sufficient light intensities reach the hippocampus through this method. Otherwise, it is unclear why a non-invasive light delivery was chosen in this case. While there now exist methods for near-infrared non-invasive delivery, blue light is notoriously prone to scattering and absorption of biological tissue. I recommend the authors use direct optic light fiber-mediated light delivery, as is common for blue light stimulation of subcortical brain regions.

• In Fig. 2, Fig. 3, Fig. 4, Fig. 5, Fig. 6, and Fig. 8.e, the authors need to show the number of mice in the groups and calculate the mean value from each mouse instead show the whole group of data was gathered from slices, astrocytes, soma, or microdomains. It is less convincing if the data came from one or two mice.

Response: We appreciate the reviewer's comment. We have now included the number of mice for each experiment in addition to the numbers of cells and slices, and this information is provided in Table S1. To improve accessibility for readers, we have also added the number of mice directly in the figure legends, while keeping within the journal's 350-word limit per legend.

• The authors used similar schematic figures to show the virus injection in Fig. 1.j, Fig. 4.d, Fig. 5.b, Fig. 8.b. The authors need to make the figures clear enough for the reader to understand their experimental design. Please show the unilateral and bilateral injections clearly in the figures.

Response: We have revised the schematic figures to make them clearer and to explicitly indicate whether unilateral or bilateral injections were used. This information has also been clarified in the figure legends and Methods section.

• Please replace Supplementary Figure 3.c with a higher resolution image. (The result of the control #1 looks a bit artificial.)

Response: Supplementary Figure 3e (previously 3c) has been replaced with a higher-resolution image.

Revised manuscript

Supplementary Figures

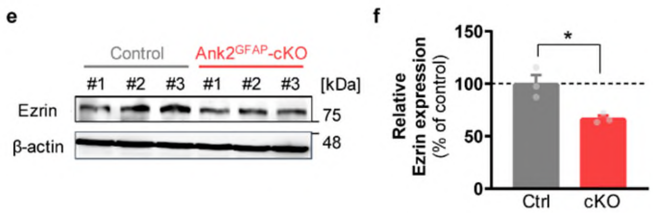

• Please do not compress the data points in Fig. 7.e and Fig. 8.j. The authors need to find a suitable y axis range to show the data clearly in the figures.

Response: In the revised figures, we have introduced a y-axis break in Fig. 7e and Fig. 8j so that lower freezing values are more clearly visible while still allowing direct comparison with the higher values.

• In Supplementary Fig. 7, the authors wrote: "Astrocytic Ank2 plays a distinct role in maintaining IP3R2 expression and function within the TrkB.T1-IP3R2 axis, which is essential for astrocyte morphogenesis via cytoskeletal rearrangements". However, it is not consistent with the figure described as IP3Rs. The authors need to summarize the figure and consistently provide a description.

Response: We have carefully re-checked the description and revised the text to ensure consistency across the figure, legend, and main text.

Finally, we would like to express our sincere gratitude to the reviewers not only for their major comments but also for their careful minor suggestions. We found these comments highly constructive, and they have helped us to improve the clarity, consistency, and overall quality of the manuscript.
